# A New Sustainable Approach to Antibiotic Resistance: Development of bis‐Quaternary Ammonium Salts From 2,4,6‐Trichloro‐1,3,5‐Triazine

**DOI:** 10.1002/cmdc.70458

**Published:** 2026-08-27

**Authors:** Rachele Rampazzo, Arianna De Luca, Manuela Facchin, Patrizio Raffa, Andrea Gattolin, Valentina Beghetto

**Affiliations:** ^1^ Department of Molecular Sciences and Nanosystems Ca’ Foscari University Venice Venice Italy; ^2^ Department of Architecture and Industrial Design University of Campania “Luigi Vanvitelli” Aversa Italy; ^3^ Crossing S.r.l. Treviso Italy; ^4^ Department of Chemical Engineering – Product Technology University of Groningen – FSE – ENTEG Groningen Netherlands; ^5^ Chimicambiente Srl Vicenza Italy; ^6^ Consorzio Interuniversitario per le Reattività Chimiche e la Catalisi (CIRCC) Bari Italy

**Keywords:** antimicrobial agents, low toxicity antimicrobials, QAS, quaternary ammonium salts, triazine

## Abstract

The development of highly effective antibiotics and antimicrobials over the centuries has greatly improved human life. However, the irresponsible use of these substances and their release into the environment have led to the emergence of resistant microorganisms. Quaternary ammonium salts (QAS), due to their high efficacy as antibacterial agents and their wide spectrum of activity as disinfectants and antiseptics, represent a possibility to avoid antibiotic resistance. In this work, new bis‐cationic QAS, having as common precursor 2,4,6‐trichloro‐1,3,5‐triazine, were synthetized showing high antimicrobial properties (bis‐TQAS). The compounds obtained were characterized by ^1^H NMR, ^13^C NMR, COSY, HSQC, HMQC, ESI‐MS, elemental analysis, and Log P determination. Their antibacterial activity was evaluated against *Klebsiella pneumoniae*, *Escherichia coli*, *Staphylococcus aureus*, and *Listeria monocytogenes*. Significant growth inhibition was achieved for all tested strains. Specifically, for the bis‐TQAS with C_10_ alkyl chain, a Log reduction of >5.70 against *S. aureus* and *L. monocytogenes* was observed, while the antibacterial Log reduction exceeded 3.60 Log for *K. pneumoniae* and *E. coli* strains. In contrast, the performance of the C_12_ alkyl chain derivative varied; nevertheless, it still demonstrated strong efficacy, with Log reductions exceeding 3.63 for the Gram‐positive strains and 4.00 for the Gram‐negative strains.

## Graphical abstract

Antibiotics improved human life, but bacterial resistance makes surfaces and packaging disinfection critical. Quaternary ammonium salts (QAS) represent a possible alternative. In this work, new bis‐cationic QAS from 2,4,6‐trichloro‐1,3,5‐triazine (bis‐TQAS) were synthesized, showing excellent inhibition against *Klebsiella pneumoniae*, *Escherichia coli*, *Staphylococcus aureus*, and *Listeria monocytogenes*.

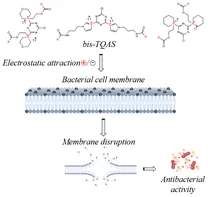

## 1 Introduction

Since the middle of the 20th century, antibiotics have revolutionized medicine by enabling effective treatment and prevention of infections once considered fatal. However, this breakthrough has brought significant challenges. Excessive and inappropriate use of antibiotics has accelerated the rise and global dissemination of antimicrobial resistance (AMR), a threat recognized by the World Health Organization as one of the top ten global health risks [1]. AMR develops through various mechanisms, including genetic mutations, enzymatic inactivation, efflux pump activation, and horizontal gene transfer, all contributing to decreased antibiotic effectiveness and increased treatment failures [2, 3, 4, 5]. AMR endangers healthcare systems worldwide and threatens to dismantle decades of medical advancements [6]. The spread of multidrug‐resistant pathogens compromises standard therapies for diseases such as tuberculosis, pneumonia, sepsis, and surgical infections [7]. Within this panorama, the development of innovative antimicrobials are of high interest for academia and the industry.

Quaternary ammonium salts (QAS) are a class of efficient antimicrobial agents with a broad‐spectrum of applications for the preparation of biomedical compounds [8, 9], cleaning and health care products [9, 10], food preservatives products [10, 11, 12], surfactants [13, 14, 15], and other applications [16, 17, 18, 19, 20], forecasted to reach 2.02 billion$ by 2030, growing at a CAGR of 5.2% between 2023 and 2030 [21].

**FIGURE 1:**
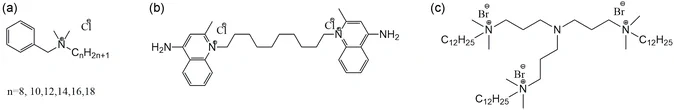
Chemical structure of (a) BAC, (b) DQ, and (c) *N*,*N*′,*N*″‐(nitrilotris(propane‐3,1‐diyl))tris(*N*,*N*‐dimethyldodecan‐1‐aminium) bromide.

QAS are divided in mono‐, bis‐, and multi‐QAS depending on the number of positive charges present [22, 23]. One of the most employed mono‐QAS is benzalkonium chloride (BAC) (Figure 1a), which, nevertheless, raises concerns due to irritancy, environmental persistence, and toxicity [10, 24]. Dequalinium chloride (DQ), a bis‐QAS (Figure 1b), contains two cationic centers and functions as an anti‐infective agent, primarily used for the treatment of localized infections [25, 26]. Conversely, multi‐QAS, such as *N*,*N*′,*N*″‐(nitrilotris(propane‐3,1‐diyl))tris(*N*,*N*‐dimethyldodecan‐1‐aminium) bromide (Figure 1c), despite higher charge density, often show reduced antimicrobial efficacy and selectivity, limiting their practical use [22, 27, 28, 29]. The antimicrobial activity of QAS strongly depends on the alkyl chain length, typically reaching higher efficiency with C_12_ and C_16_ alkyl chains [17, 30].

Despite their efficacy, the increased use of mono‐QAS, especially during the COVID‐19 pandemic, has raised concerns on multidrug‐resistant phenomena and environmental impacts, particularly in wastewater and soil [31, 32, 33]. Further, toxicological issues, such as respiratory irritation, skin sensitization, and possible endocrine disruption, associated with mono‐QAS exposure [34], are driving the development of safer alternative antimicrobials combining efficacy with reduced cytotoxicity and environmental impact. Recently, the synthesis of advanced Gemini QAS (dual‐head surfactants) [29, 35] and QAS‐functionalized materials [36], such as metal–organic frameworks and polymer composites, offers synergistic antibacterial effects and enhanced stability compared to conventional formulations [37], alongside improved biodegradability and lower toxicity, positioning them as promising candidates for next‐generation biocides. In this context, bis‐QAS have emerged as an efficient and environmentally sustainable alternative specifically to mono‐QAS, as they generally possess superior antimicrobial efficiency and a lower propensity to induce bacterial resistance compared to their monocationic counterparts [11, 16, 24, 26, 27, 28, 38, 39, 40, 41, 42, 43, 44, 45, 46, 47, 48, 49, 50, 51, 52]. From a synthetic point of view, it should be mentioned that conventionally, mono‐, bis‐, and multi‐QAS are synthesized via nucleophilic substitution reaction of tertiary amines and alkyl halides, employing harsh reaction conditions and often toxic chemicals, followed by complex purification steps [53]. In this concern, recently our research group has reported the first example of triazine‐based bis‐QAS, which can be synthesized at room temperature and without the use of toxic alkyl bromides, with a simple, easily tunable, atom‐efficient methodology [38, 39]. Triazine bis‐QAS (bis‐TQAS) were prepared based on the general rule that antimicrobials should have a long alkyl chain within their structure, to achieve high efficiency and lower bacterial resistance when compared to conventional mono‐QAS [38, 39, 54].

**FIGURE 2:**
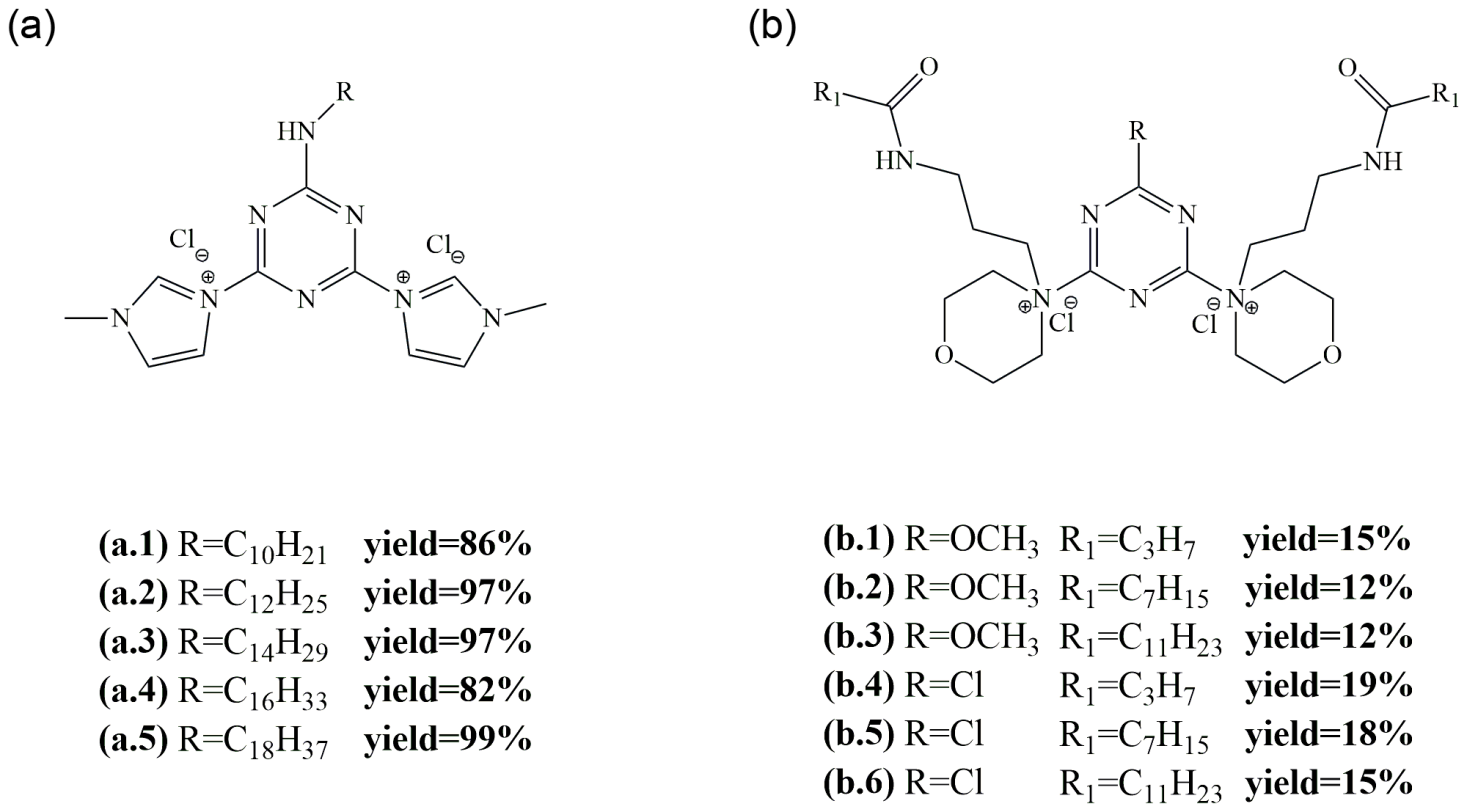
Antimicrobial bis‐TQAS previously reported in the literature [39].

Different 3,3′‐(6‐(alkylamino)‐1,3,5‐triazine‐2,4‐diyl)bis(1‐methyl‐1*H*‐imidazol‐3‐ium)quaternary ammonium salts were synthesized starting from 2,4,6‐trichloro‐1,3,5‐triazine and amidoamines (Figure 2). The selection of the *N*‐alkylamine is strategic to achieve a stable and antimicrobial bis‐TQAS, as previously reported in the literature [38, 39]. Triazines are highly reactive chemical compounds extensively used in the synthesis of a broad range of fine chemicals, dyes, herbicides, and polymers [55, 56, 57, 58, 59, 60]. Since 1998, following the synthesis of the first triazine‐derived QAS, 4‐(4,6‐dimethoxy‐1,3,5‐triazin‐2‐yl)‐4‐methylmorpholinium chloride (DMTMM), by Kunishima and Kaminsky, triazines have emerged as important activators in dehydro‐condensation reactions for the formation of amides and esters [61, 62, 63]. Despite being a QAS, the potential use of DMTMM or triazine‐based QAS as antimicrobial agents has never been systematically explored. In fact, DMTMM is a highly reactive reagent with poor stability in solution and is therefore inadequate as an antimicrobial.

According to the literature, the stability and reactivity of triazine QAS like DMTMM can be modulated by changing the structure of the tertiary amine used [30, 64, 65]. For instance, when the amine is a tertiary *N*‐alkylmorpholine bearing an alkyl chain with at least three carbon atoms or *N*‐methylimidazole, two classes of bis‐TQAS can been prepared, showing high antimicrobial activity, low toxicity, and stability in time (Figure 2) [38, 39].

Since no morpholine with *N*‐alkyl chains having more than three carbon atoms is commercially available, an alternative way to easily prepare a set of tertiary *N*‐alkylmorpholine with variable carbon length was devised starting from commercially available 4‐(3‐aminopropyl)morpholine and different aliphatic carboxylic acids (**b.1**)–(**b.2**) (Figure 2) [38, 39].

## 2 Results and Discussion

**SCHEME 1:**
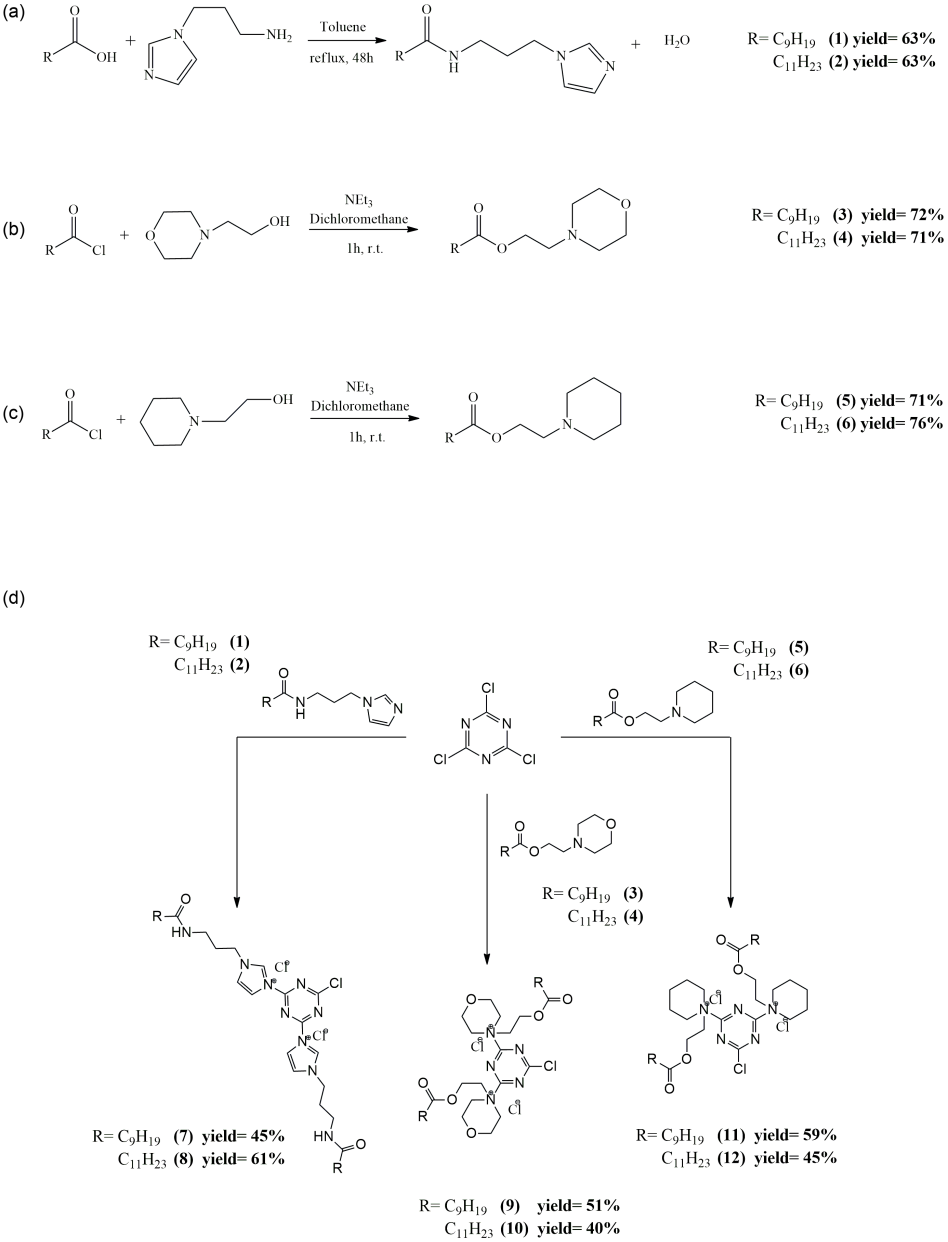
Synthesis of (a) 1‐(3‐propyl‐imidazole)decanamide (1) and *N*‐(3‐(1*H*‐imidazol‐1‐yl)propyl)dodecanamide (2), (b) *N*‐(2‐ethylmorpholine)decanoate (3) and *N*‐(2‐ethylmorpholine)dodecanoate (4), (c) ethyl piperidine decanoate (5) and ethyl piperidine laurate (6), and (d) bis‐TQAS (7)‐(12).

This work intends to widen the range of bis‐TQAS available and verify their efficiency toward different bacterial species. Six new bis‐TQAS were prepared by reaction of 2,4,6‐tricholoro‐1,3,5‐triazine with different amides (**1**)–(**2**) and esters (**3**)–(**6**) specifically prepared to have C_10_–C_12_ alkyl chains on the bis‐TQAS (**7**)–(**12**) (Scheme 1). Amides and esters were prepared by condensation of commercially available 4‐(2‐hydroxyethyl morpholine), 1‐(3‐aminopropyl)imidazole, or 1‐(2‐hydroxyethyl) piperidine with carboxylic acids or chlorides with C_10_ or C_12_ alkyl chains at reflux temperature for 48 h in good yield (>60%). Then, bis‐TQAS were synthesized (**7**)–(**12**) by reaction of 2,4,6‐trichloro‐1,3,5‐triazine and (**1**)–(**6**), at room temperature by 1 h (Scheme 1d). All compounds were fully characterized by ^1^H NMR, ^13^C NMR, COSY, HSQC, HMQC, elemental, and ESI‐MS analysis confirming that only [M]^2+^ compounds are formed (>75% total intensity) (see Supporting Information) [38, 39, 64, 65, 66].

The antibacterial activity of the prepared bis‐TQAS derivatives was evaluated using the ASTM E2149 [67] standard method. This dynamic contact protocol was intentionally selected over other methods more commonly cited in the literature [38, 39, 68, 69, 70, 71, 72] to ensure methodological compliance with the ECHA Guidance set by the Biocidal Products Regulation (BPR)—Volume II: Efficacy (Parts B + C, Version 5.0, Appendix 6) [73] and the European Biocidal Products Regulation (Regulation (EU) No 528/2012, BPR) [74].

While the guidelines recommend specific tests for solid materials (example ISO 22196), the BPR grants flexibility to utilize alternative available standards when required by sample characteristics. Given the water insolubility, limited dispersibility, and specific physical constraints of the solid specimens under investigation, alternative protocols demanding defined test specimen geometries were inapplicable.

Therefore, ASTM E2149 has proven to be the most appropriate standard, as it is specifically designed to evaluate the antibacterial efficacy of insoluble and nonleaching materials (such as polymer films, coatings, and packages) under conditions that simulate real‐world applications [75, 76]. The method involves incubating the treated samples in a bacterial suspension of a known concentration under constant mixing, thereby ensuring continuous dynamic contact and uniform exposure between the active sample surface and the microorganisms. Following a defined contact period of 24 h, selected for all experiments, the viable bacterial population was quantified using the plate count method on an appropriate growth medium, and based on the counts obtained, the percentage and logarithmic reductions in viable bacteria were calculated and compared with an untreated control subjected to identical conditions. Moreover, selecting the ASTM standard is highly relevant considering the prospective utilization of these formulations in packaging materials, an industry where ASTM guidelines constitute the established technical reference for material validation.

Consequently, the assay was performed against four representative bacterial strains: *Klebsiella pneumoniae* (*ATCC 4352*) and *Escherichia coli* (*ATCC 25922*) as Gram‐negative models, and *Staphylococcus aureus* (*ATCC 6538*) and *Listeria monocytogenes* (*ATCC 35152*) as Gram‐positive models. Finally, the data were compared with the activity of benzalkonium chloride (BAC), which was tested under equivalent conditions as a positive benchmark control.

**FIGURE 3:**
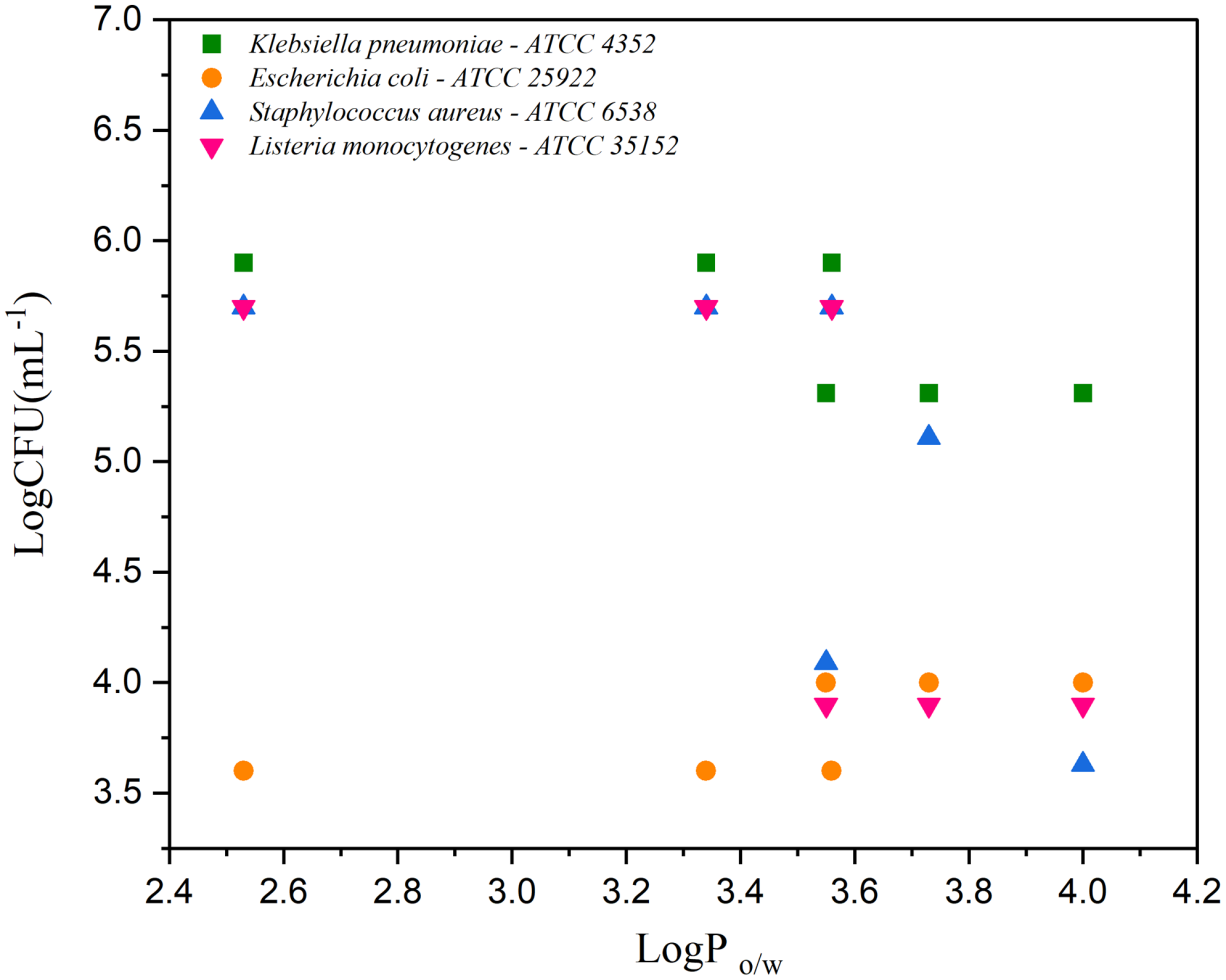
Representation of Log *P*_O/W_ versus Log CFU (mL^−1^) of bis‐TQAS.

Antibacterial efficacy was expressed both as logarithmic (Log) reduction (Table 1 and Figure 3) and as percentage reduction (Table S2), reflecting the extent of microbial inactivation attributable to the antimicrobial treatment. These values were calculated as the difference in viable bacterial counts between the treated samples and the untreated controls. The 24‐h dynamic contact assays confirmed the strong antibacterial activity of the bis‐TQAS prepared, all of which achieved a bacterial reduction >99.9%, demonstrating comparable efficacy to BAC benchmark control (Table S1).

**TABLE 1:** Logarithmic bacterial reduction under dynamic contact conditions, and Log *P*_O/W_ (*n*‐octanol/water).

Compound	Log CFU, mL^−1^ a				Log *P* b
	*Klebsiella pneumoniae*	*Escherichia coli*	*Staphylococcus aureus*	*Listeria monocytogenes*	
BAC	>5.00	>4.00	>4.20	>3.90	2.90
(7)	>5.90	>3.60	>5.70	>5.70	2.53
(8)	>5.31	>4.00	4.09	>3.90	3.55
(9)	>5.90	>3.60	>5.70	>5.70	3.34
(10)	>5.31	>4.00	>5.11	>3.90	3.73
(11)	>5.90	>3.60	>5.70	>5.70	3.56
(12)	>5.31	>4.00	3.63	>3.90	4.00

a Logarithmic bacterial reductions under dynamic contact conditions.

b Log *P*_O/W_ calculated using online platform ALOGPS 2.1 [77, 78, 79].

The analysis of bacterial data identified the C_10_ alkyl chain bis‐TQAS derivatives as the most effective QAS against *K. pneumoniae*, *S. aureus*, and *L. monocytogenes*, with Log reductions exceeding 5.90, 5.70, and 5.70, respectively (Table 1). When evaluated against BAC, these C_10_ analogs exhibited superior antibacterial performance across almost all strains. The sole exception was *E. coli*, where the C_10_ and C_12_ derivatives showed equivalent efficacy to each other and to the control, displaying log reductions exceeding 3.60 and 4.00, respectively, suggesting that the outer membrane of this Gram‐negative strain is more susceptible to a more lipophilic chain. Meanwhile, the C_12_ derivatives displayed a performance that remained comparable with BAC across all tested microorganisms.

Overall, these data are in line with or even superior to most efficient antimicrobial materials reported in the literature, such as polymers, biopolymers, quaternary ammonium compound, and silicone catheter tubing, which typically show logarithmic reduction values between 1 and 7.5 [69, 71, 80, 81]. For example, Cheong et al. reported the synthesis of quaternary ammonium compounds with varying alkyl chain lengths, such as alkyl (C12–16) dimethyl benzyl ammonium chloride, which exhibited a Log >3.0 after 24 h against *S. aureus and E. coli*. This performance was lower than the bis‐TQAS compounds synthetized in this work with a C_10_ and C_12_ alkyl chain [82]. Similarly, Hermosilla et al. described polymers characterized by the presence of mono‐QAS in their chains that achieved bacterial reduction up to 99% and Log reduction >3 [71].

Additionally, Log *P*_O/W_ of bis‐TQAS tested were calculated in order to highlight possible correlations between the different lengths of the alkyl chain, hydrophobicity or lipophobicity, and antimicrobial activity. Plotting Log *P*_O/W_ versus Log CFU of bis‐TQAS (Figure 3) evidences a correlation between the length of the alkyl chain, Log *P*, and antibacterial activity. Previous studies have shown that moderate hydrophobicity, corresponding to Log *P*_O/W_ between 1 and 3, promotes antimicrobial activity [83, 84, 85, 86]. This trend is reflected in the obtained values, which range from 2.53 to 4.00 (Table 1 and Figure 3).

Specifically, the C_10_ alkyl chain derivatives (**7**), (**9**), and (**11**) showed the highest antibacterial efficacy with Log *P* values of 2.53, 3.34, and 3.56, respectively, achieving complete bacterial reduction within 24 h against three of the four strains. In contrast, a longer chain with the compounds (**8**), (**10**), and (**12**) systematically increased the lipophilicity, pushing the Log *P* values up to 3.55, 3.73, and 4.00, respectively. Moreover, experimental hydrophobicity measurements (CHI Log *P*) of quaternary heteronium salts reported in the literature show positive Log *P* values (≈0.17–4.93), in line with the values of bis‐TQAS reported in this work [86, 87].

These findings underscore the critical requirement of a finely tuned hydrophilic–lipophilic balance (HLB) to maximize antimicrobial performance. While compounds with a Log *P*_O/W_ > 3 possess pronounced lipophilic traits that theoretically enhance cell membrane permeation, excessive hydrophobicity conversely compromises aqueous solubility, thereby impairing bioavailability [84, 85]. Consequently, the interplay between alkyl chain length and bioactivity is governed by a multiparametric physicochemical matrix, which accounts for the observed deviations from conventional structure–activity relationship (SAR) models in QAS [27, 38, 39, 88, 89, 90]. Further mechanistic investigations are currently underway to fully elucidate this behavior.

## 3 Conclusion

This work focuses on the synthesis and antimicrobial activity of novel triazine‐based bis‐QAS as efficient and reliable alternatives against antibiotic resistance. Bis‐TQAS were prepared with an easy and atom‐efficient strategy starting from 2,4,6‐trichloro‐1,3,5‐triazine as a common precursor, a compound known for its ability to rapidly form ammonium salts at room temperature. The synthesis involves two main steps: the preparation of the precursors (1–6) and its subsequent reaction with 2,4,6‐trichloro‐1,3,5‐triazine. The compounds obtained exhibited excellent antibacterial performance against both Gram‐positive and Gram‐negative bacterial strains. Notably, all tested bis‐TQAS achieved a bacterial reduction >99.9% within 24 h. Among the synthesized compounds, those with a C_10_ alkyl chain showed superior antibacterial activity, achieving a Log reduction of  >5.9 against *K. pneumoniae* (*ATCC 4352*) and greater than >5.7 against *S. aureus* (*ATCC 6538*) *and L. monocytogenes* (*ATCC 35152*). Despite the limited number of compounds, the study establishes clear proof of concept and provides a foundation for future expansion of this chemical family as additional precursors become available or are specifically synthesized. Moving forward, it would be valuable to explore the effects of varying the number of cationic charges and alkyl chain lengths on antibacterial performance. Future studies will investigate the application of these compounds in antimicrobial formulations for surface coatings, such as plastic films, paper, and metals for packaging application.

## Funding

This study was supported by the National Recovery and Resilience Plan (PNRR), for the 39th PhD cycle “National Interest PhD Program in Design for Made in Italy: Identity, Innovation, and Sustainability”, administrative headquarter University of Campania “Luigi Vanvitelli”, Caserta (Italy).

## Conflicts of Interest

The authors declare no conflicts of interest.

## Supporting information

Supplementary Material

## Data Availability

The data that support the findings of this study are available from the corresponding author upon reasonable request.
