# Supplementary material for: A New Sustainable Approach to Antibiotic Resistance: Development of bis‐Quaternary Ammonium Salts From 2,4,6‐Trichloro‐1,3,5‐Triazine
Source: ChemMedChem. 2026 Aug 27;21(17):e70458. doi: 10.1002/cmdc.70458 (PMC13521741; doi:10.1002/cmdc.70458)

Supporting Information

A New Sustainable Approach to Antibiotic-Resistance: Development of Bis-Quaternary Ammonium Salts (QAS) from 2,4,6-Trichloro-1,3,5-Triazine

Rachele Rampazzo, Arianna De Luca, Manuela Facchin, Patrizio Raffa, Andrea Gattolin, Valentina Beghetto*

Table of Contents

I. General Information

II. . Synthesis of Compounds 1-14

III. Elemental Analysis

IV. Biological Assays

IV. ^{1}H, ^{13}C NMR, COSY, HMQC, HSQC

I. General information

Reagents and solvents were purchased from Sigma-Aldrich, without further purification. All reactions were carried out under ambient atmosphere with reagent grade solvents. The NMR spectra were recorded using a Bruker UltraShield 400 spectrometer. The deuterate solvents used to make the NMR spectra were $CDCl_3$ and DMSO, with an internal reference of 7.26 ppm and 2.50 ppm for ^1H NMR; and 77.16 ppm and 39.52 ppm for ^{13}C NMR respectively. High-Resolution mass spectrometry data were obtained with an Agilent technologies Infinity LC-ESI/MS. Elemental analysis for carbon, hydrogen, nitrogen and sulfur (CHNS) were performed using UNICUBE organic elemental analyzer (Elementar).

I.1 Synthesis of 1-(3-propyl imidazole)decanamide (1)

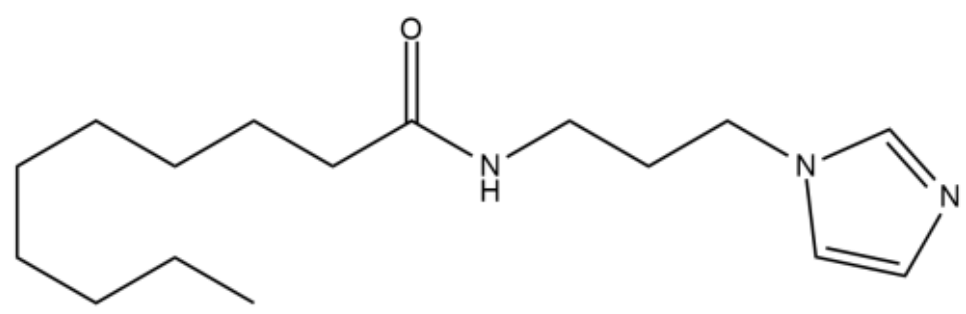

1-(3-propyl imidazole)decanamide
Chemical Formula: $C_{16}H_{29}N_3O$
Molecular Weight: 279,42

In a 50 mL single neck flask equipped with a Dean-Stark apparatus, 1g of decanoic acid (5.81mmol) and 20.30 ml of Toluene were added and stirred until complete dissolution. Then 1.02 mL of 1-(3-aminopropyl) imidazole (8.12 mmol) was added and heated at reflux for 48h under magnetic stirring. After 48h, the solvent was removed by evaporation. Three extractions were made with a saturated sodium bicarbonate water solution and dichloromethane. The organic phase was collected, dried over $MgSO_4$ and the solvent was removed to give a colourless viscous oil (yield 63%), which solidifies in cold conditions. ^1H NMR, ^{13}C NMR were recorded and are reported in section IV.

I.2 Synthesis of *N*-(3-(1H-imidazol-1-yl)propyl)dodecanamide (2)

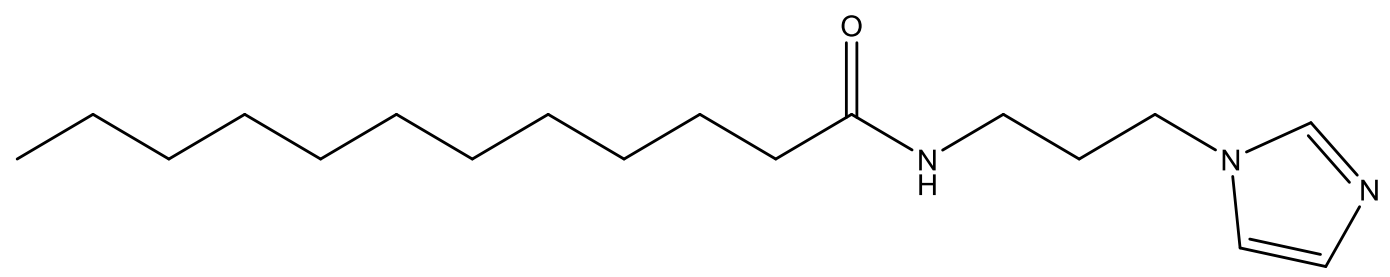

N-(3-(1H-imidazol-1-yl)propyl)dodecanamide
Chemical Formula: $C_{18}H_{33}N_3O$
Molecular Weight: 307,48

In a 50 mL single neck flask equipped with a Dean-Stark apparatus, 1g of lauric acid (5 mmol) and 20 ml of toluene were added and stirred until complete dissolution. Then 0.83mL of 1-(3-aminopropyl)imidazole (6.95 mmol) were added and heated at reflux for 48h under magnetic stirring. After 48h, the solvent was removed by evaporation. Then 10 mL of a NaOH water solution

(30% w/w) were added to neutralize the unreacted acid and the white solid formed was recovered by filtration. The resulting *N*-(3-(1H-imidazol-1-yl)propyl)dodecanamide (2) was a white solid, (isolated yield 63%). ^{1}H NMR, ^{13}C NMR were recorded and are reported in section IV.

I.3 Synthesis of Decanoyl chloride (a)

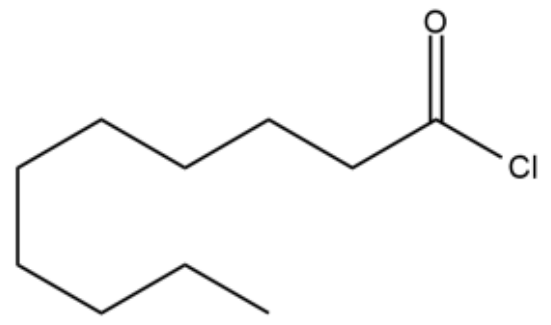

Decanoyl chloride
Chemical Formula: $C_{10}H_{19}ClO$
Molecular Weight: 190,71

In a 100ml single neck flask, were placed 10 g of decanoic acid (0.06 mol) and 7.12 ml of thionyl chloride (0.1 mol). The reaction mixture was stirred for 1h at room temperature and after for 3h at 65°C. The resulting product was a dark liquid (isolated yield 60%). ^{1}H NMR, ^{13}C NMR spectra were recorded and are reported in section IV.

I.4 Synthesis of *N*-(2-Ethylmorpholine) decanoate (3)

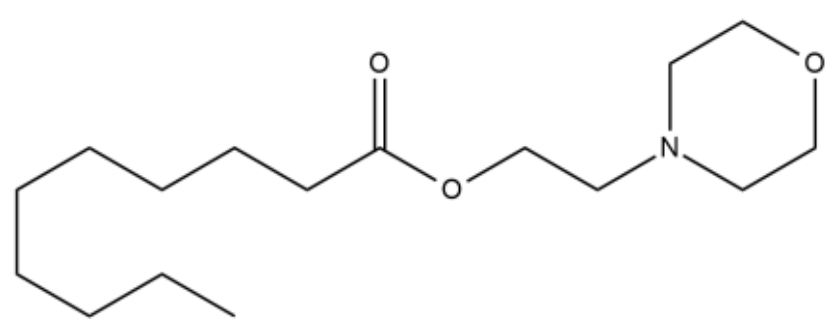

N-(2-Ethylmorpholine)decanoate
Chemical Formula: $C_{16}H_{31}NO_3$
Molecular Weight: 285,42

In a 100ml single neck flask, were placed 1.47 ml of 4-(2-hyrossi ethyl morpholine) (11.2 mmol), 2.21 ml of triethylamine (16.00 mmol) and 50 ml of dichloromethane. Subsequently, 3.00 ml of decanoyl chloride (16.00 mmol) previously dissolved in 10 ml of dichloromethane was added by dripping. The solution was cooled with an ice bath, under stirring for 30 minutes, and afterwards kept at room temperature for 1 h. Then 10 ml of distilled water were added, and the solution was transferred to a separating funnel, and the dichloromethane recovered, dried over magnesium sulphate ($MgSO_4$) and solvent removed under vacuum. The viscous liquid obtained was extracted with 50 ml of diethyl ether and 10 ml of a saturated sodium bicarbonate water solution. The organic phase was isolated through a separating funnel, dried over $MgSO_4$ and the solvent removed under vacuum. The product obtained was a dark orange oil (isolated yield 72%). ^{1}H NMR, ^{13}C NMR were recorded and are reported in section IV.

I.5 Synthesis of Lauroyl chloride (b)

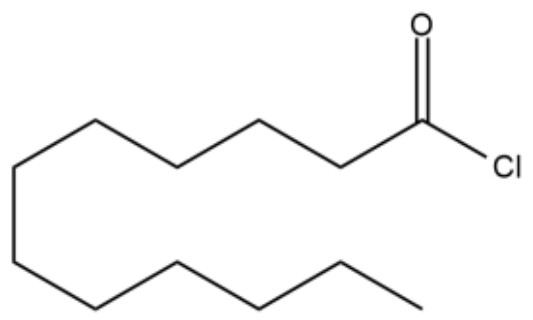

Lauroyl chloride
Chemical Formula: $C_{12}H_{23}ClO$
Molecular Weight: 218,76

In a 100ml single neck flask, were placed 10 g of lauric acid (0.05 mol) and 7.12 ml of thionyl chloride (0.1 mol), and the mixture was kept under stirring for 1h at room temperature and for further 3h at 65°C. The resulting product was a dark liquid (isolated yield 60%). ^{1}H NMR, ^{13}C NMR were recorded and are reported in section IV.

I.6 Synthesis of *N*-(2-Ethylmorpholine)dodecanoate (4)

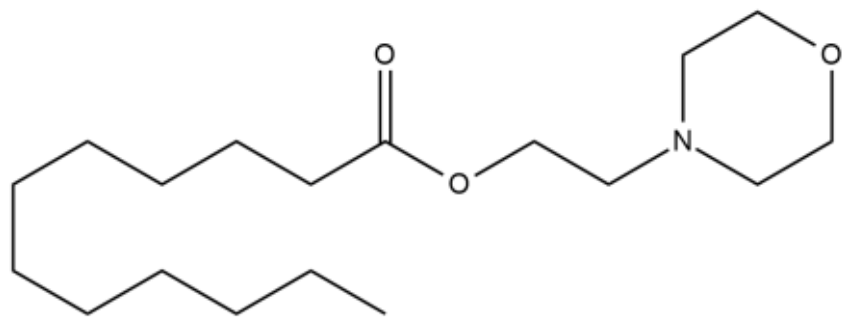

N-(2-Ethylmorpholine)dodecanoate
Chemical Formula: $C_{18}H_{35}NO_3$
Molecular Weight: 313,48

In a 100ml single neck flask, were placed 0.83 ml of 4-(2-hydroxyethyl morpholine) (6.9 mmol), 1.43ml of triethylamine (10.3 mmol) and 50 ml of dichloromethane. Subsequently, 2.30 ml of lauryl chloride (10.0 mmol) previously dissolved in 10 ml of dichloromethane were added by dripping. The solution was cooled with an ice bath, under stirring for 30 minutes, and further kept at room temperature for 1h. Then 10 ml of distilled water were added, and the solution transferred to a separating funnel. The organic fraction was separated, dried over $MgSO_4$ anhydrous and the solvent removed under vacuum. In the viscous liquid obtained, an extraction was carried out with 50 ml of diethyl ether and 10 ml of a saturated sodium bicarbonate water solution. The organic phase was isolated through a separating funnel, dried over $MgSO_4$ anhydrous and the solvent removed under vacuum. The product obtained is an orange oil (isolated yield 71%). ^{1}H NMR, ^{13}C NMR were recorded and are reported in section IV.

I.7 Synthesis of Ethyl piperidine decanoate (5)

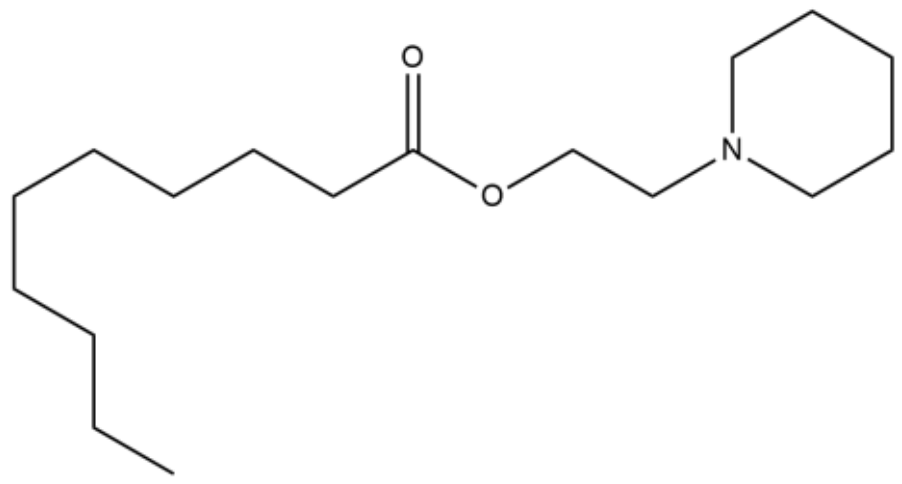

Ethyl piperidine decanoate
Chemical Formula: $C_{17}H_{33}NO_2$
Molecular Weight: 283,45

In a 100ml single neck flask, were placed 0.96 ml of 1-(2-hydroxyethyl) piperidine (7.42 mmol), 1.40 ml of triethylamine (10.00 mmol) and 50 ml of dichloromethane. Subsequently, 2.00 ml of decanoyl chloride (11.00 mmol) previously dissolved in 10 ml of dichloromethane was added by dripping. The solution was cooled with an ice bath, under stirring for 30 minutes, and further kept at room temperature for 1h. Then 10 ml of distilled water were added, and the solution was transferred to a separating funnel. The organic fraction was separated, dried over $MgSO_4$ anhydrous and the solvent removed under vacuum. The viscous liquid obtained was extracted with 50 ml of diethyl ether and 10 ml of a saturated sodium bicarbonate water solution. The organic phase was isolated through a separating funnel, dried over $MgSO_4$ and the solvent was removed by high-vacuum pump. The product obtained is an orange oil (isolated yield 72%). ^{1}H NMR, ^{13}C NMR were recorded and are reported in section IV.

I.8 Synthesis of Ethyl piperidine laurate (6)

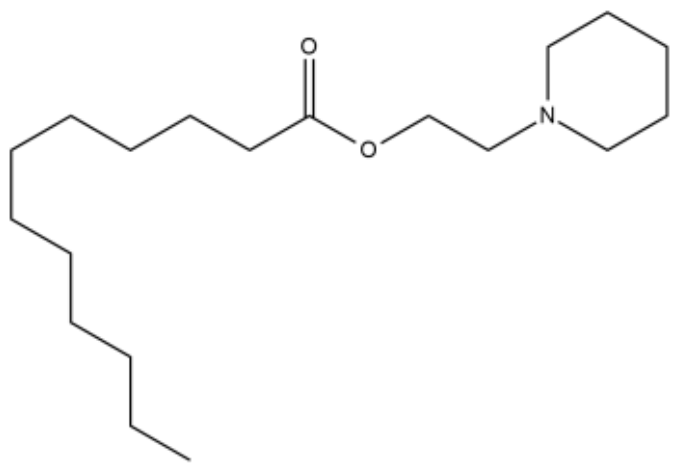

Ethyl piperidine laurate
Chemical Formula: $C_{19}H_{37}NO_2$
Molecular Weight: 311,50

In a 100ml single neck flask, were placed 0.90 ml of 1-(2-hydroxyethyl) piperidine (6.9 mmol), 1.43ml of triethylamine (10.3 mmol) and 50 ml of dichloromethane. Subsequently, 2.30 ml of lauryl chloride (10.0 mmol) previously dissolved in 10 ml of dichloromethane was added by dripping. The solution was cooled with an ice bath, under stirring for 30 minutes, and further kept at room temperature for 1h. Then 10 ml of distilled water were added, and the solution was transferred to a separating funnel. The organic fraction was separated, dried over $MgSO_4$ anhydrous and the solvent

removed under vacuum. The viscous liquid obtained, was extracted with 50 ml of diethyl ether and 10 ml of a a saturated sodium bicarbonate water solution. The organic phase was isolated through a separating funnel, dried over $MgSO_4$ and the solvent was removed by high-vacuum pump. The product obtained is a light-orange oil (isolated yield 76%). ^{1}H NMR, ^{13}C NMR were recorded and are reported in section IV.

I.9 Synthesis of 2-chloro-4,6-(*N*-(3-propyl imidazole)decanamide)-1,3,5-triazine (7)

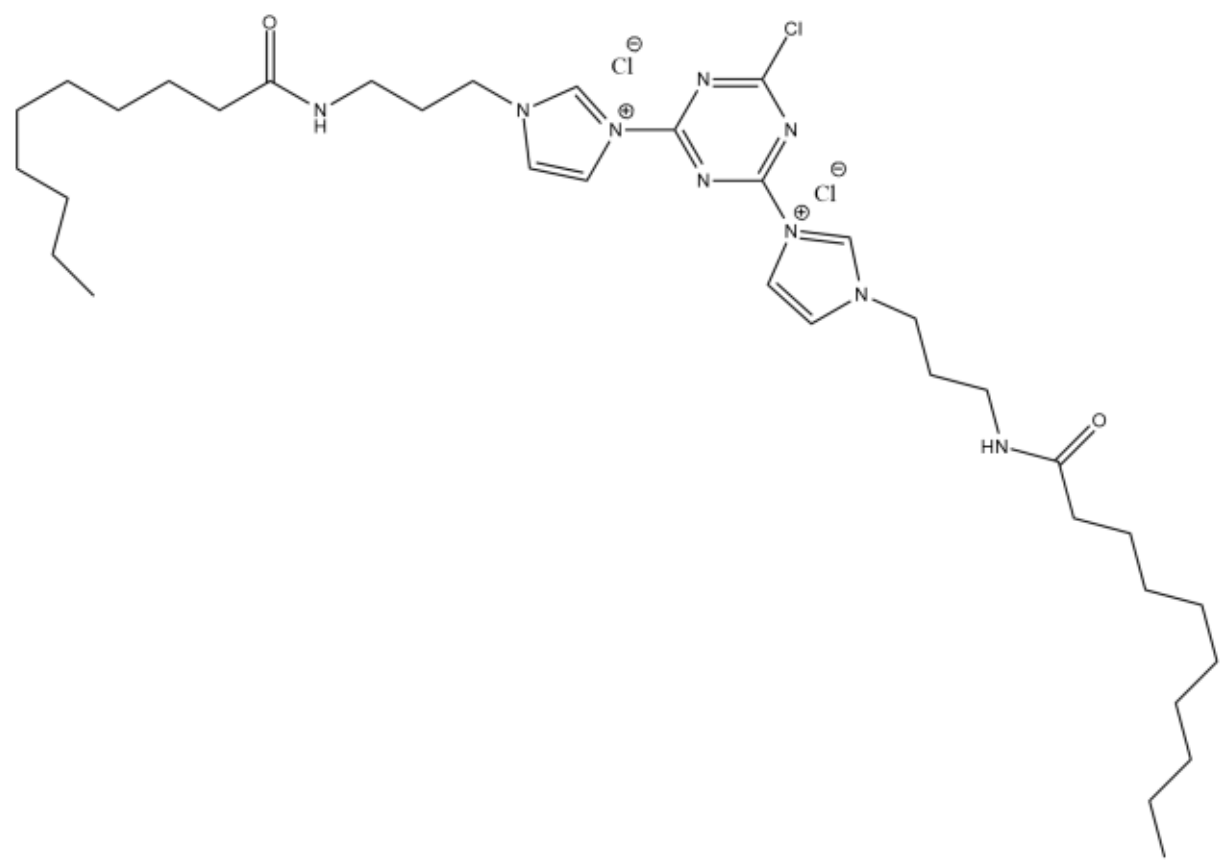

2-chloro-4,6-(*N*-(3-propyl imidazole)decanamide)-1,3,5-triazine
Chemical Formula: $C_{35}H_{58}Cl_3N_9O_2$
Molecular Weight: 743,25

In a 50 mL single neck flask, were placed 0.50 g (1.78 mmol) of **(1)**, 5.70 mL of acetone and 0.35ml (2.09 mmol) of DIPEA. The solution was cooled with an ice bath and then a solution of 0.11 g (0.59 mmol) of CC in 2.00 mL of acetone was added dropwise. It was kept under stirring for 4h. The resulting dark yellow precipitate (isolated yield 45%) was then filtered with a Gooch funnel and washed with cold acetone. ESI-MS (m/z): 396.28$[M]^{2+}$.^{1}H NMR, ^{13}C NMR, COSY, HSQC, HMQC were recorded and are reported in section IV.

ESI-MS

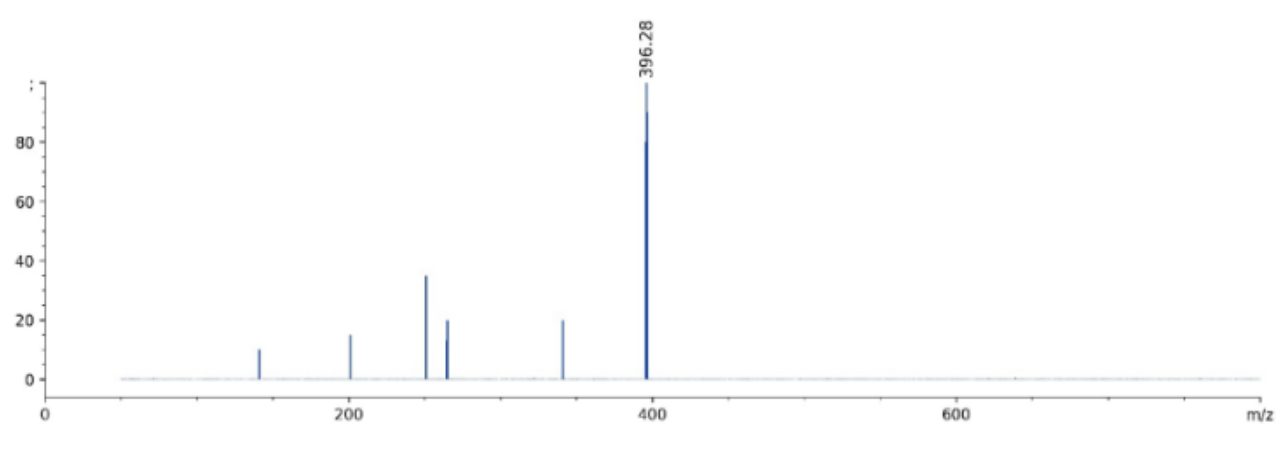

I.10 Synthesis of 2-chloro-4,6-(*N*-(3-propyl imidazole)dodecanamide)-1,3,5-triazine chloride (8)

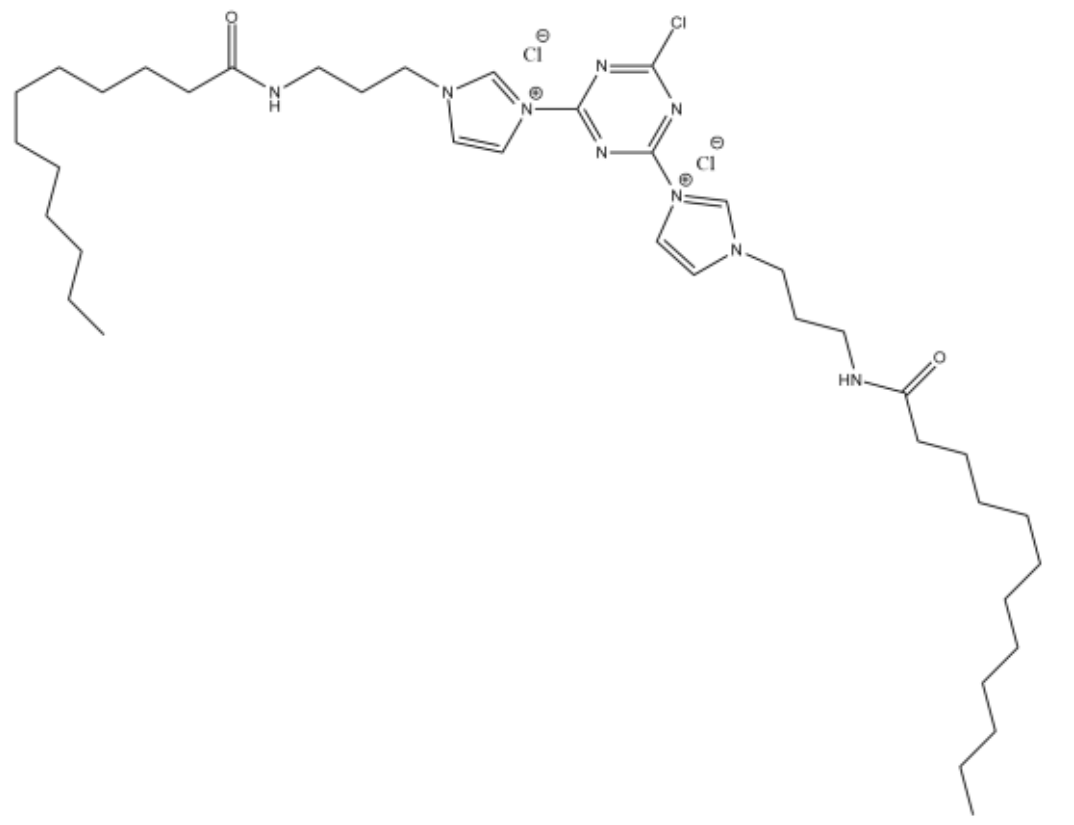

2-chloro-4,6-(N-(3-propyl imidazole)dodecanamide)-1,3,5-triazine chloride
Chemical Formula: $C_{39}H_{67}N_9O_2$
Molecular Weight: 799,36

In a 50 mL single neck flask, were placed 0.50 g (1.62 mmol) of **(2)**, 5.63 mL of acetone and 0.17ml (1.83 mmol) of diisopropylethylamine (DIPEA). The solution was cooled with an ice bath and then a solution of 0.1 g (0.54 mmol) of Cyanuric Chloride (CC) in 1.98 mL of acetone was added dropwise and the mixture kept under stirring for 4h. The resulting white precipitate was then filtered with Gooch funnel and washed with cold acetone. The resulting product was a white powder (isolated yield 61%). ^{1}H NMR, ^{13}C NMR, COSY, HSQC, HMQC were recorded and are reported in section IV.

I.11 Synthesis of 2-chloro-4,6-(*N*-(2-ethylmorpholine)decanoate)-1,3,5-triazine chloride (9)

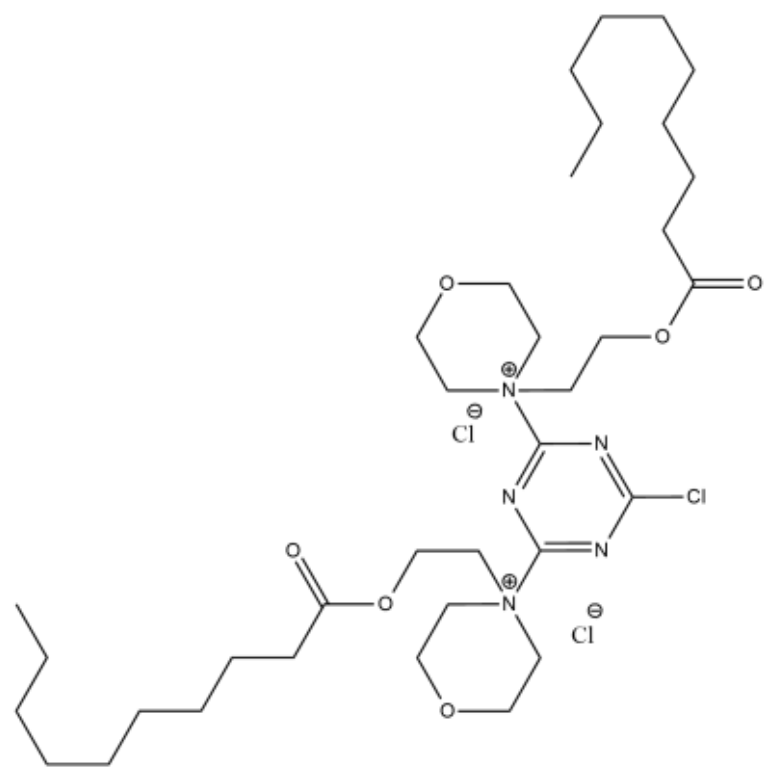

2-chloro-4,6-(*N*-(2-ethylmorpholine)decanoate)-1,3,5-triazine chloride
Chemical Formula: $C_{35}H_{62}Cl_3N_5O_6$
Molecular Weight: 755,26

In a 50 mL single neck flask, were placed 2.00 g (7.21 mmol) of **(3),** 22.5 mL of acetone and 0.96ml (6.81 mmol) of DIPEA. The solution was cooled with an ice bath and then a solution of 0.43 g (2.33 mmol) of CC in 2.00 mL of acetone was added dropwise, and the mixture kept under stirring for 4h. The resulting yellow precipitate (isolated yield 51%) was then filtered with Gooch

funnel and washed with cold acetone. ESI-MS (m/z): 437.33$[M]^{2+}$. ^{1}H NMR, ^{13}C NMR, COSY, HSQC, were recorded and are reported in section IV.

ESI-MS

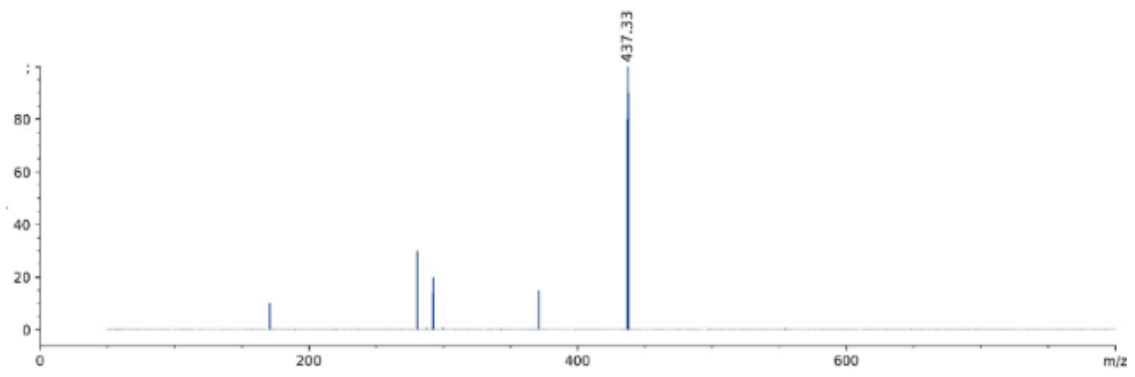

I.12 Synthesis of 2-chloro-4,6-(*N*-(2-ethylmorpholine)dodecanoate)-1,3,5-triazin chloride (10)

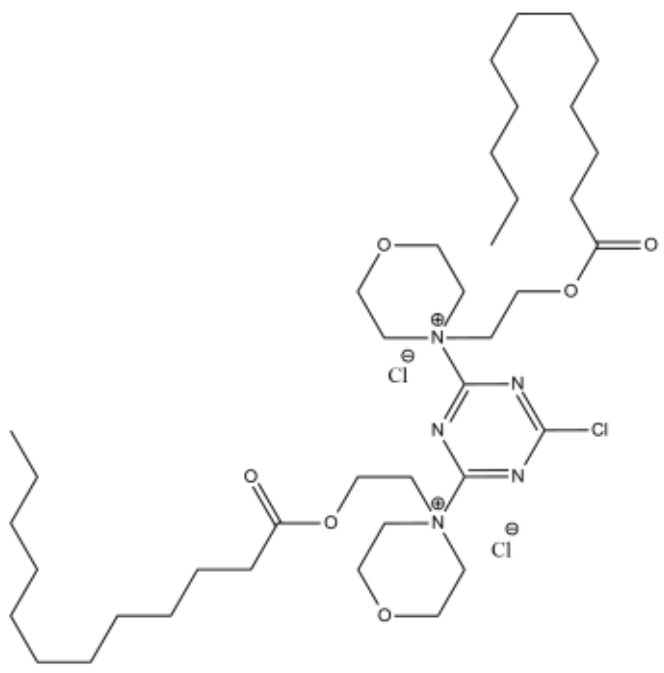

2-chloro-4,6-(*N*-(2-ethylmorpholine)dodecanoate)-1,3,5-triazin chloride
Chemical Formula: $C_{39}H_{70}ClN_5O_6$
Molecular Weight: 811,36

In a 50 mL single neck flask, were placed 1.00 g (3.19 mmol) of (4), 11.2 mL of acetone and 0.61 mL (3.61 mmol) of DIPEA. The solution was cooled with an ice bath and then a solution of 0.19 g (1.06 mmol) of CC in 3.8 mL of acetone was added dropwise and the mixture kept under stirring for 4h. The resulting white precipitate (isolated yield 40%) was then filtered with Gooch funnel and washed with cold acetone. ^{1}H NMR, ^{13}C NMR, COSY, HSQC, HMQC were recorded and are reported in section IV.

I.13 Synthesis of 2-chloro-4,6-(*N*-(ethylpiperidine)decanoate)-1,3,5-triazin chloride (11)

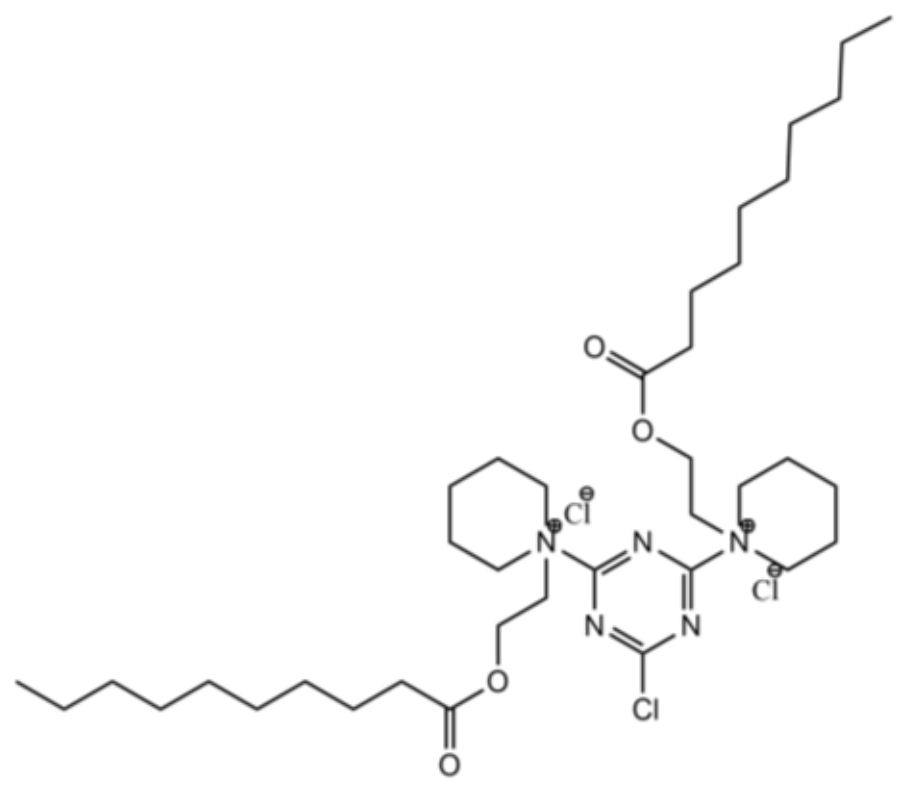

2-chloro-4,6-(*N*-(ethylpiperidine)decanoate)-1,3,5-triazin chloride
Chemical Formula: $C_{37}H_{66}Cl_3N_5O_4$
Molecular Weight: 751,31

In a 50 mL single neck flask, were placed 1.00 g (3.51 mmol) of **(5),** 11.60 mL of acetone and 0.68ml (3.97 mmol) of DIPEA. The solution was cooled with an ice bath and then a solution of 0.22 g (1.17 mmol) of CC in 4.31 mL of acetone was added dropwise and the mixture kept under stirring for 4h. The resulting yellow precipitate (isolated yield 59%) was then filtered with Gooch funnel and washed with cold acetone ESI-MS (m/z): 410.30$[M]^{2+}$. ^{1}H NMR, ^{13}C NMR, COSY, HSQC, HMQC were recorded and are reported in section IV.

ESI-MS

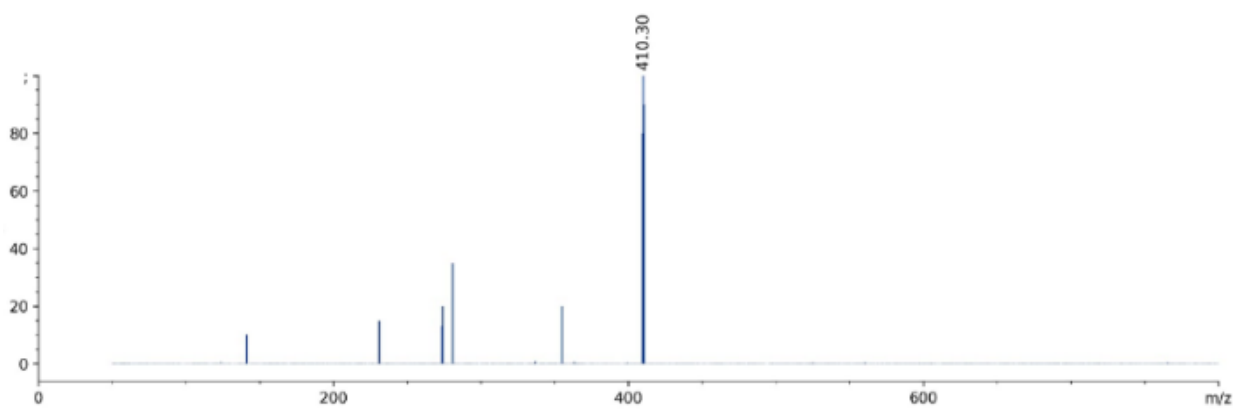

I.14 Synthesis of 2-chloro-4,6-(*N*-(ethylpiperidine)dodecanoate)-1,3,5-triazin chloride (12)

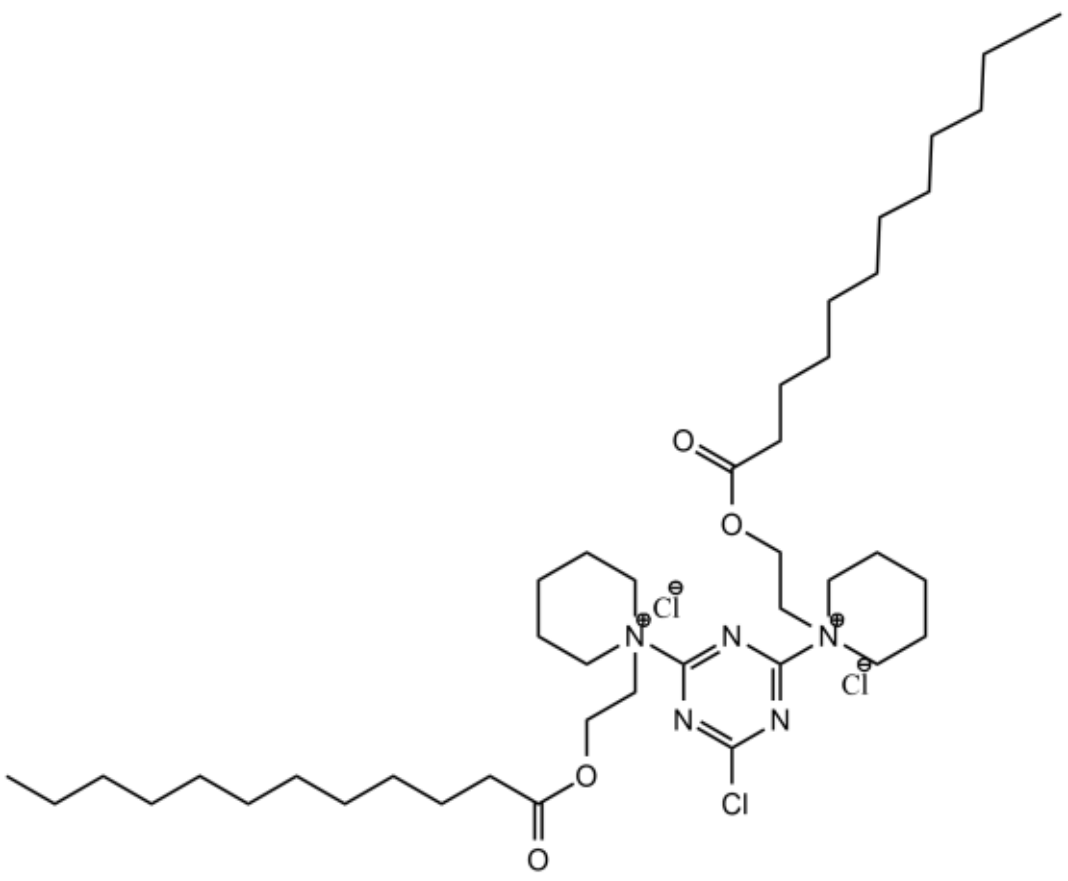

2-chloro-4,6-(*N*-(ethylpiperidine)dodecanoate)-1,3,5-triazin chloride
Chemical Formula: $C_{41}H_{74}Cl_3N_5O_4$
Molecular Weight: 807,42

In a 50 mL single neck flask, were placed 0.6 g (1.92 mmol) of **(6)**, 5.2 mL of acetone and 0.33ml (2.26 mmol) of DIPEA. The solution was cooled with an ice bath and then a solution of 0.11 g (0.64 mmol) of CC in 2.8 mL of acetone was added dropwise and the mixture kept under stirring for 4h. The resulting light-pink precipitate (isolated yield 42%) was then filtered with Gooch funnel and washed with cold acetone. ^{1}H NMR, ^{13}C NMR, COSY, HSQC, HMQC were recorded and are reported in section IV.

II. Biological Assays

The antimicrobial activity of the samples was assessed according to ASTM E 2149. For these tests a contact period of 24 hours was chosen (although the method would provide a contact time of 1h), using bacterial strains *Klebsiella pneumoniae - ATCC 4352, Escherichia coli - ATCC 25922, Staphylococcus aureus - ATCC 6538, Listeria monocytogenes - ATCC 35152* and data compared with the activity of Benzalkonium Chloride (BAC) as control.

The procedure is reported below.

A bacterial suspension was prepared in buffer at a concentration of 1.5–3 × 10^5 UFC/mL. Then 0.2 g of sample was introduced into sterile containers, to which 50 mL of the bacterial suspension was added. At the same time, a control container containing exclusively the bacterial inoculum was set up.

The bacterial concentration at time zero (T_0) was determined by taking aliquots from the control container and subjecting them to serial dilutions, followed by seeding by inclusion on PCA (Plate Count Agar) nutrient medium. All containers, including the control, were subsequently placed on a horizontal stirrer at maximum speed for 1 hour. At the end of the contact time, new serial dilutions were performed from each container, followed by sowing on a plate according to the same methods used for T_0. Plates were incubated at 35 ± 2 °C for 24 hours. At the end of incubation, the bacterial

colonies developed were counted. The percentage of bacterial reduction was calculated by comparing the number of surviving microorganisms in the treated samples with that detected in the control containing the inoculum alone.

Table SI1. Percentage of bacterial reduction under dynamic contact conditions

**Compound**	**R%**[(b)]			
	**Klebsiella pneumoniae**	**Escherichia coli**	**Staphylococcus aureus**	**Listeria monocytogenes**
**BAC**	> 99.9	> 99.9	> 99.9	> 99.9
**(7)**	>99.999	> 99.9992	>99.999	> 99.999
**(8)**	>99.999	> 99.9	>99.992	> 99.9
**(9)**	>99.999	> 99.9992	>99.999	> 99.999
**(10)**	>99.999	> 99.9	>99.999	> 99.9
**(11)**	>99.999	> 99.9992	>99.999	> 99.999
**(12)**	>99.999	> 99.9	>99.977	> 99.9

LogP calculation method

Compounds lipophilicity (LogP) was estimated using ALOGPS 2.1 (https://vcclab.org/lab/alogps/start.html). Input SMILES codes were generated using ChemDraw Ultra (version 12.0, CambridgeSoft Corp., Cambridge, MA, USA).

SMILES codes:

(**7**):
CCCCCCCCCC(NCCCN1C=C[N+](C2=NC([N+]3=CN(CCCNC(CCCCCCCCC)=O)C=C3)=NC(Cl)=N2)=C1)=O.[Cl-].[Cl-]
(**8**):
CCCCCCCCCCCC(NCCCN1C=C[N+](C2=NC([N+]3=CN(CCCNC(CCCCCCCCCCC)=O)C=C3)=NC(Cl)=N2)=C1)=O.[Cl-].[Cl-]
(**9**):
ClC1=NC([N+]2(CCOC(CCCCCCCCC)=O)CCOCC2)=NC([N+]3(CCOC(CCCCCCCCC)=O)CCOCC3)=N1.[Cl-].[Cl-]
(**10**):
ClC1=NC([N+]2(CCOC(CCCCCCCCCCC)=O)CCOCC2)=NC([N+]3(CCOC(CCCCCCCCCCC)=O)CCOCC3)=N1.[Cl-].[Cl-]
(**11**):
ClC1=NC([N+]2(CCOC(CCCCCCCCC)=O)CCCCC2)=NC([N+]3(CCOC(CCCCCCCCC)=O)CCCCC3)=N1.[Cl-].[Cl-]
(**12**):
ClC1=NC([N+]2(CCOC(CCCCCCCCCCC)=O)CCCCC2)=NC([N+]3(CCOC(CCCCCCCCCCC)=O)CCCCC3)=N1.[Cl-].[Cl-]

III. Elemental Analysis

The elemental analysis was carried out on all the bis-QASs synthesized with the purpose of evaluating the purity and chemical composition of the compounds. The theoretical values of the ratio between the elements carbon and nitrogen are reported in Table SI2, considering the salt formation with two positive charges, and the experimental ones obtained by the analysis. The results confirmed the formation of bis-cationic salts.

Table SI2. Theoretical and experimental C/N values obtained by Elemental analyses of the synthesized QAS compounds.

QAS	Theoretical value C/N	Experimental value C/N
**(7)**	3.33	3.11
**(8)**	3.78	3.69
**(9)**	6.00	5.88
**(10)**	6.68	6.62
**(11)**	6.34	6.43
**(12)**	7.21	6.96

IV. ^{1}H and ^{13}C NMR Spectra

IV.1 1-(3-propyl imidazole) (1)

^{1}H NMR

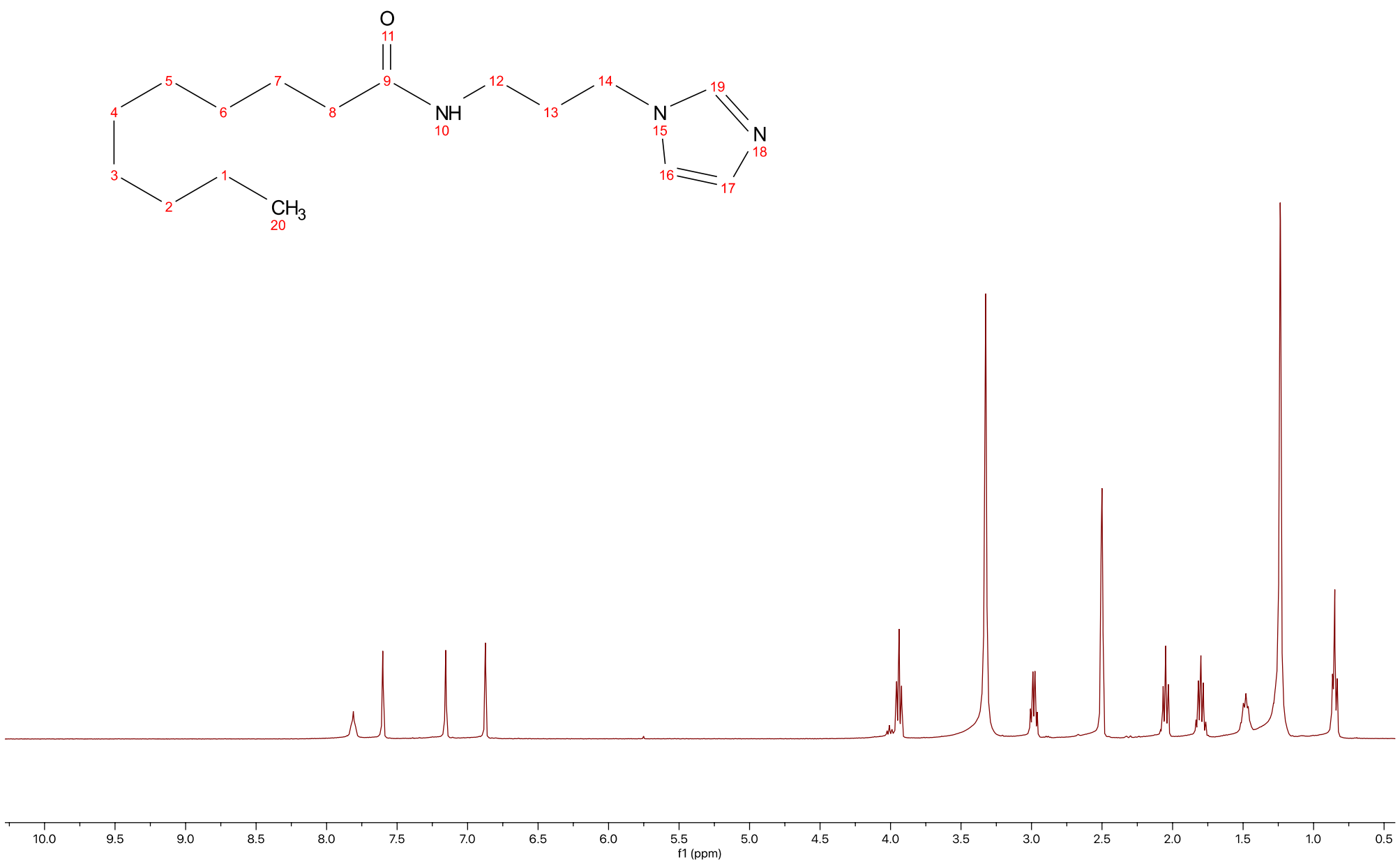

^{1}H NMR (400 MHz, DMSO-d$_6$) δ (ppm): 7.82 (1H, t, H$_{10}$), 7.60 (1H, s, H$_{19}$), 7.15 (1H, s, H$_{16}$), 6.87 (1H, s, H$_{17}$), 3.94 (2H, t, H$_{14}$), 2.98 (2H, q, H$_{12}$), 2.04 (2H, t, H$_8$), 1.80 (2H, q, H$_{13}$), 1.47 (2H, t, H$_7$), 1.23 (16H, s, H$_{2\text{-}6}$), 0.88 (3H, t, H$_1$).

^{13}C NMR

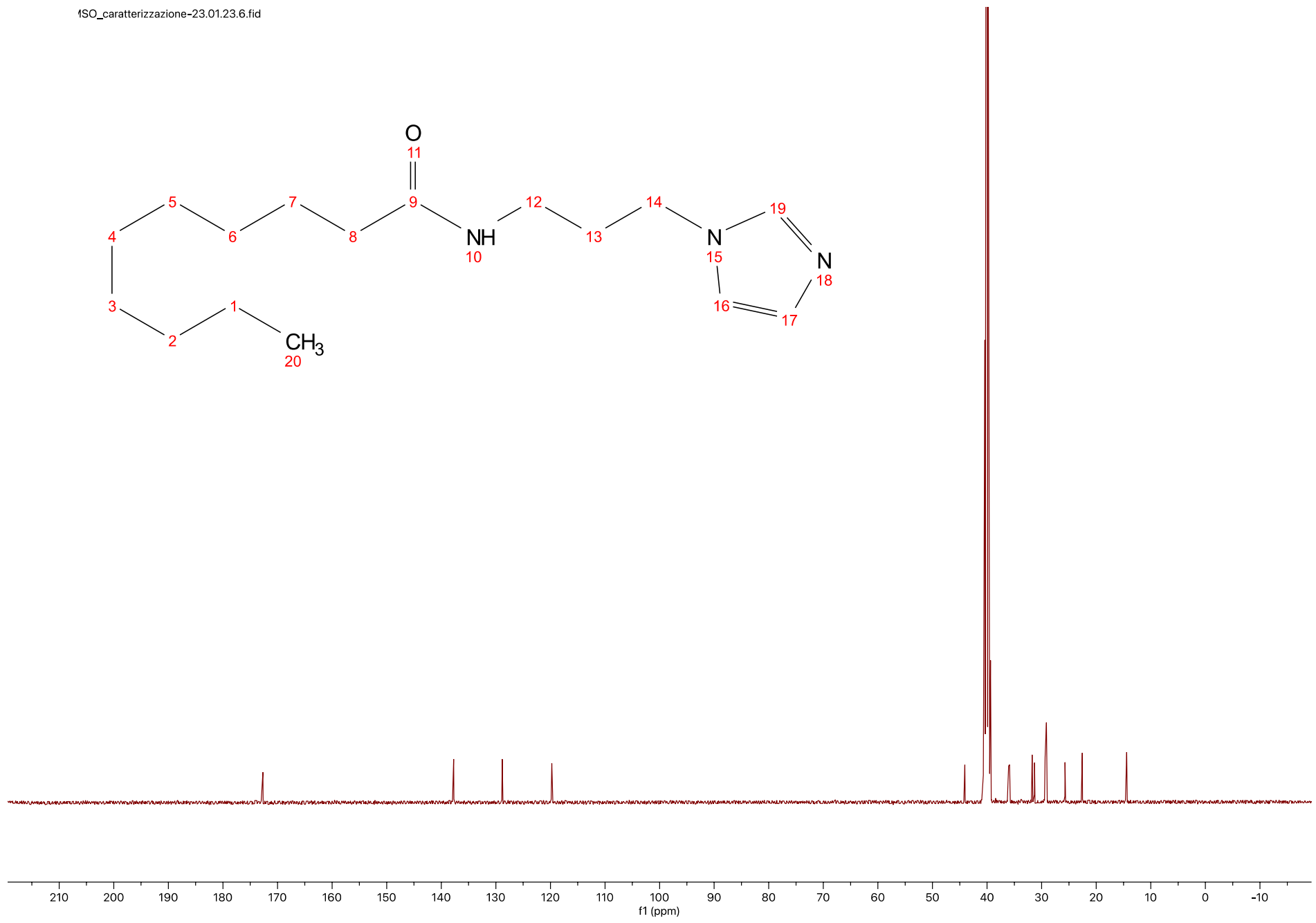

^{13}C NMR (400 MHz, DMSO-d$_6$) δ (ppm): 172.25 (C$_9$), 137.28 (C$_{19}$), 128.26 (C$_{16}$), 119.22 (C$_{17}$), 43.66 (C$_{14}$), 35.64 (C$_{12}$), 35.43 (C$_8$), 30.84 (C$_{13}$), 20.98 (C$_{3\text{-}6}$), 25.29 (C$_7$), 22.12 (C$_2$), 13.95 (C$_1$).

IV.2 *N*-(3-(1H-imidazol-1-yl)propyl)dodecanamide (2)

^{1}H NMR

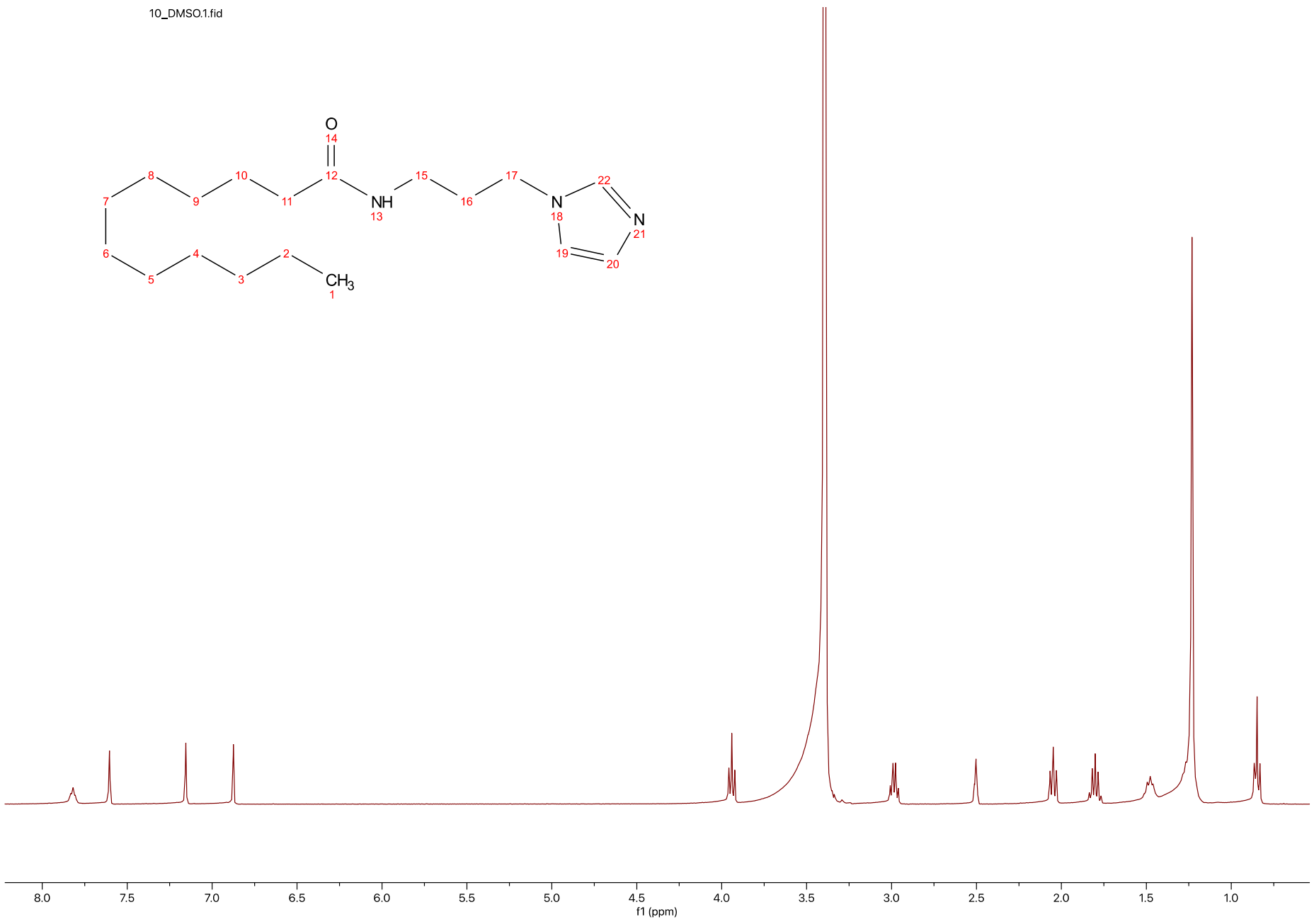

^{1}H NMR (400 MHz, DMSO-d$_6$) δ (ppm): 7.82 (1H, t, H13), 7.60 (1H, s, H22), 7.15 (1H, s, H19), 6.87 (1H, s, H20), 3.94 (2H, t, H17), 2.98 (2H, q, H15), 2.04 (2H, t, H11), 1.80 (2H, q, H16), 1.47 (2H, t, H10), 1.23 (16H, s, H2-9), 0.88 (3H, t, H1).

^{13}C NMR

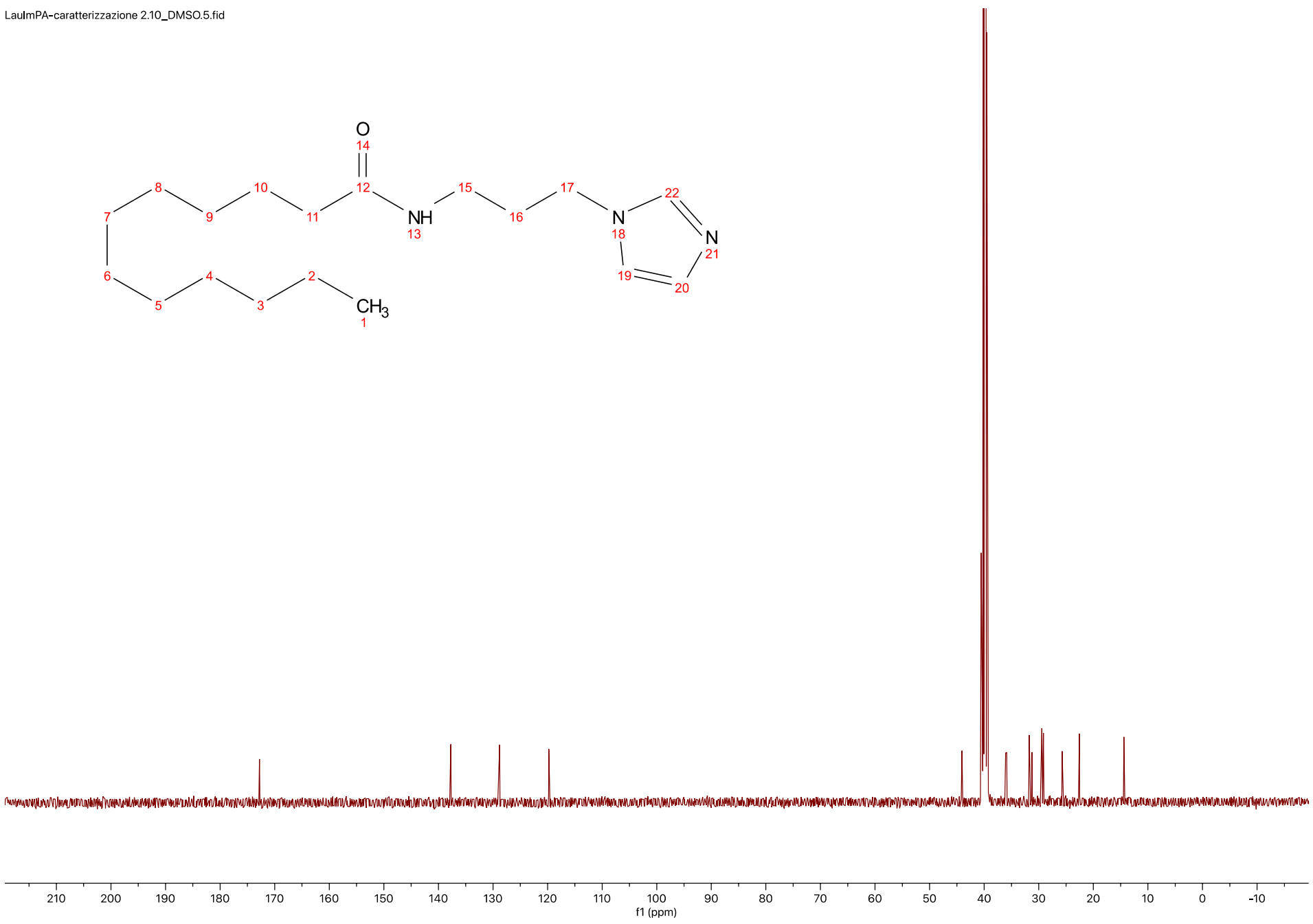

^{13}C NMR (400 MHz, DMSO-d$_6$) δ (ppm): 172.34 (C10), 137.28 (C22), 128.26 (C19), 119.22 (C20), 43.66 (C17), 35.64 (C15), 35.43 (C11), 30.84 (C16), 20.98 (C3-9), 25.29 (C10), 22.12 (C2), 13.95 (C1).

IV.3 Decanoyl chloride (a)

^{1}H NMR

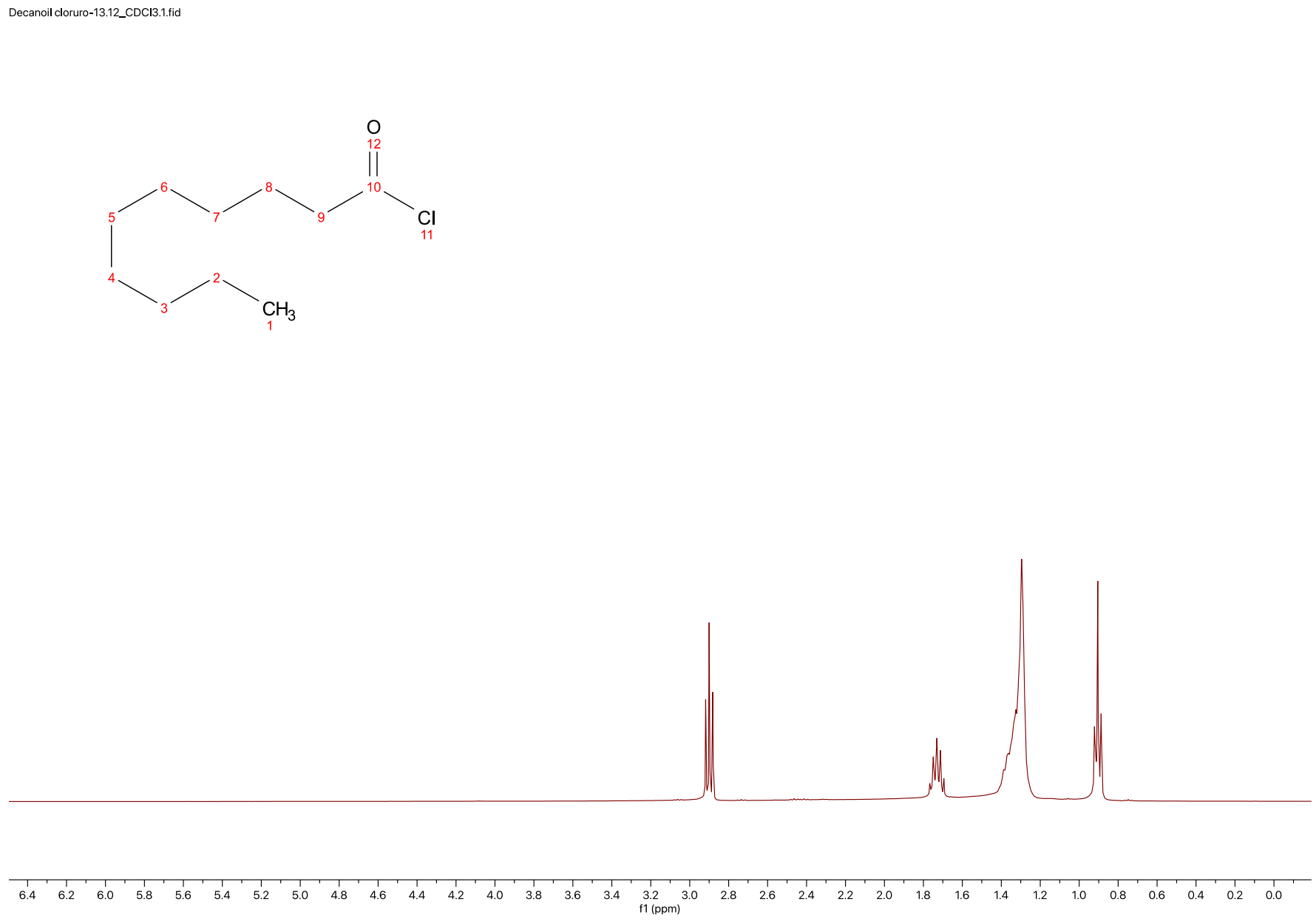

^{1}H NMR (400 MHz, $CDCl_3$) δ (ppm): 2.89 (2H, t, H9), 1,71 (2H, qH8), 1.27 (16H, s, H2-7), 0.88 (3H, t, H1).

^{13}C NMR

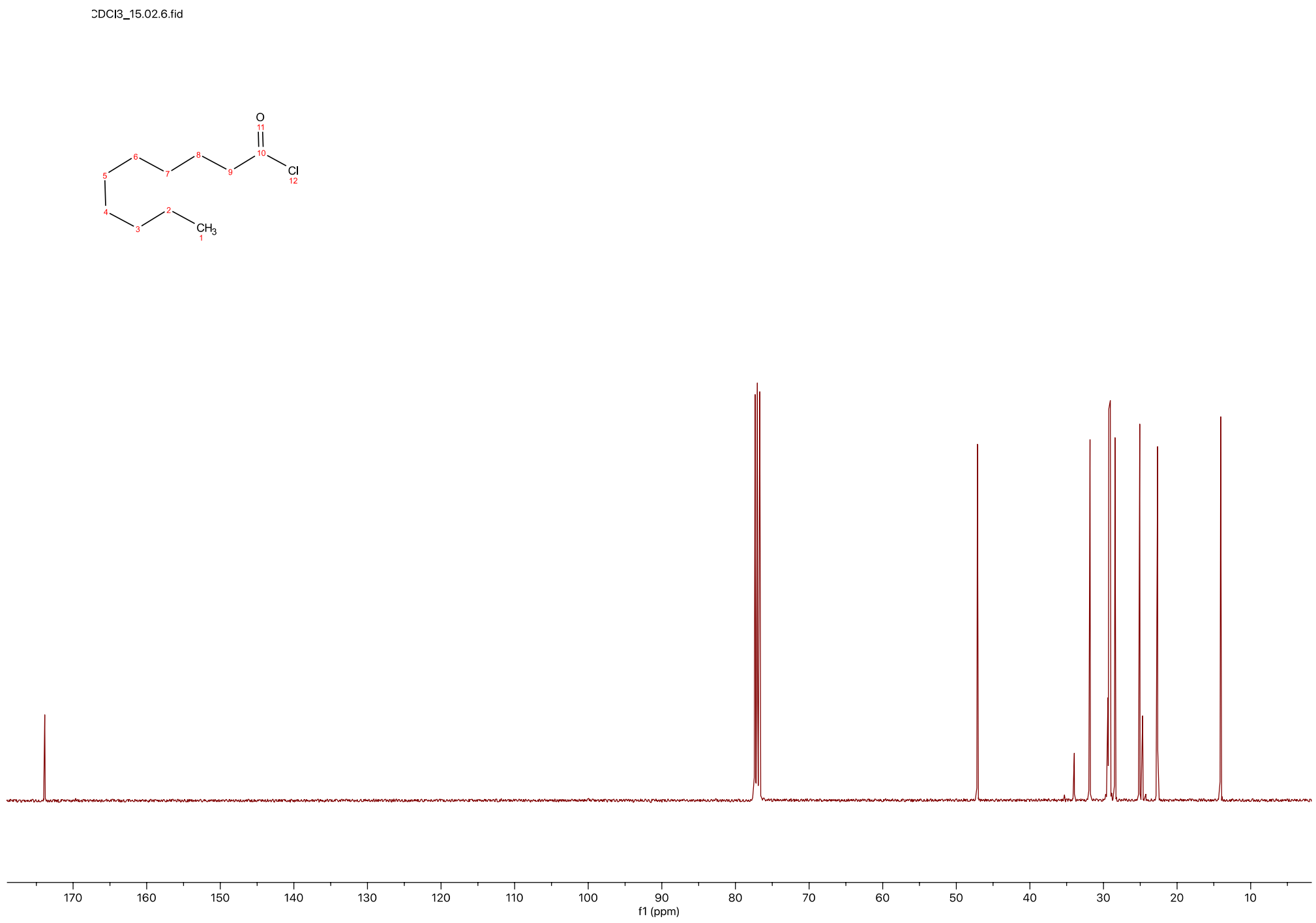

^{13}C NMR (400 MHz, $CDCl_3$) δ (ppm): 173.9 (C10), 47.28 (C9), 32.08 (C3), 25.30 (C10), 22.8 (C2), 14.26 (C1).

IV.4 *N*-(2-Ethylmorpholine) decanoate (3)

^{1}H NMR

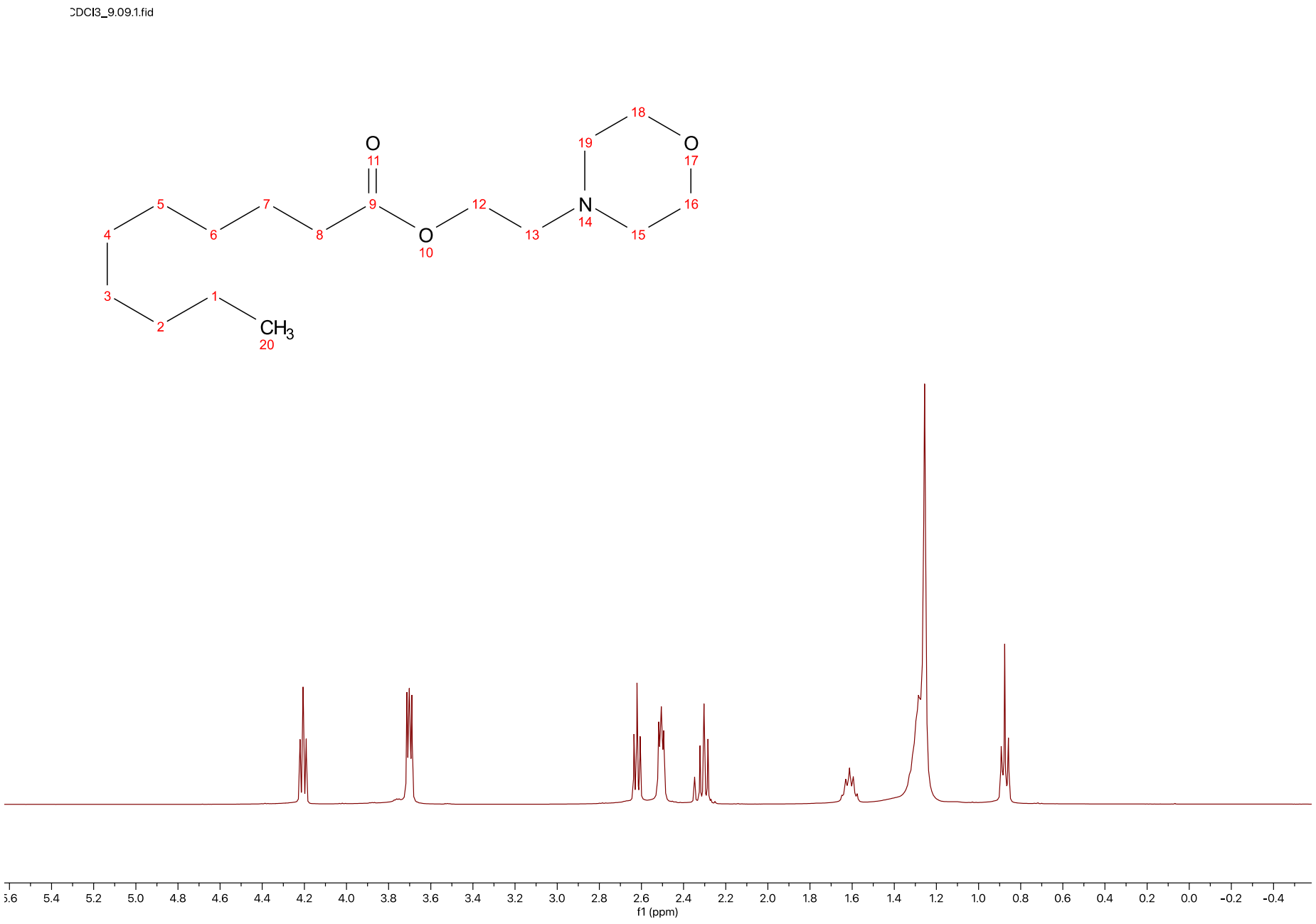

^{1}H NMR (400 MHz, $CDCl_3$) δ (ppm): 4.19 (2H, t, H12), 3.68 (4H, t, H 18-16), 2.60 (2H, t, H13), 2.48 (4H, m, H 19-15), 2.28 (2H, t, H8), 1.60 (2H, m, H7), 1.24 (16H, s, H 1-8), 0.88 (3H, t, H20).

^{13}C NMR

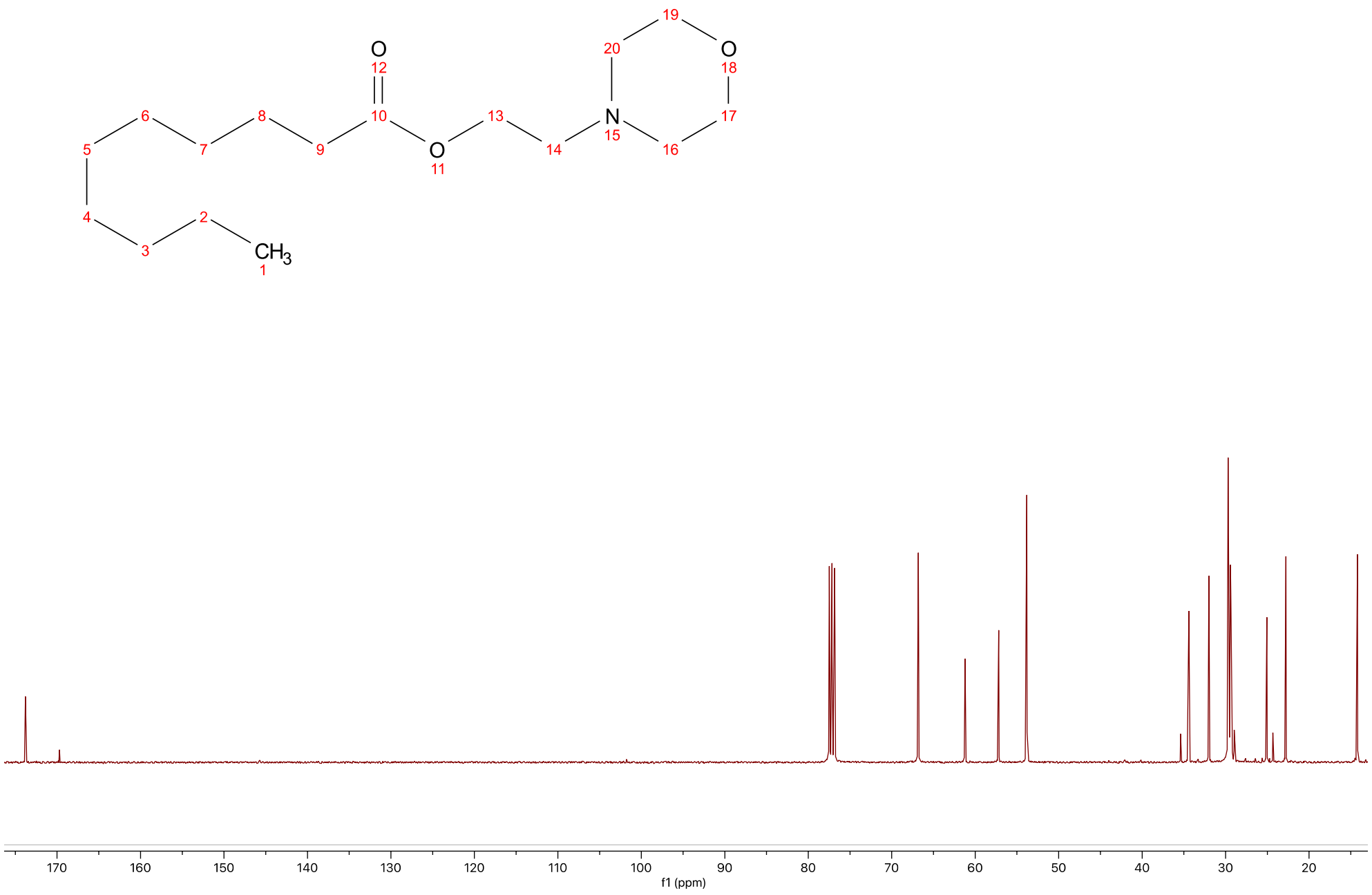

^{13}C NMR (400 MHz, $CDCl_3$) δ (ppm): 173.77 (C10), 66.65 (C19-C17), 61.22 (C13), 57.12 (C14), 53.64 (C20-C16), 34.39 (C9), 32.01 (C7), 29.70 (C3-C6), 25.05 (C8), 22.78 (C2), 14.19 (C1).

IV.5 Lauroyl chloride (b)

^{1}H NMR

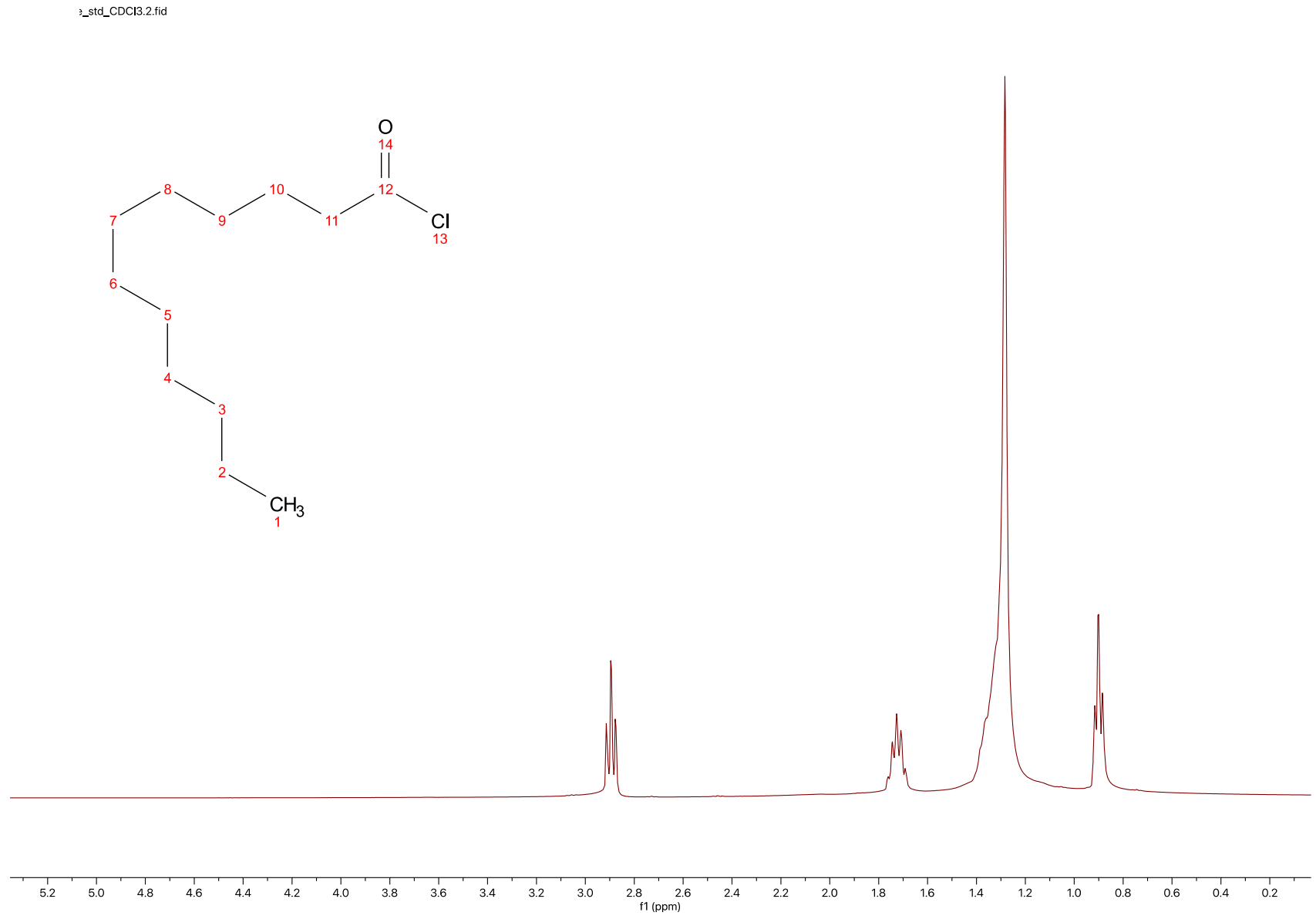

^{1}H NMR (400 MHz, $CDCl_3$) δ (ppm): 2.89 (2H, t, H11), 1,71 (2H, qH10), 1.27 (16H, s, H2-9), 0.88 (3H, t, H1).

^{13}C NMR

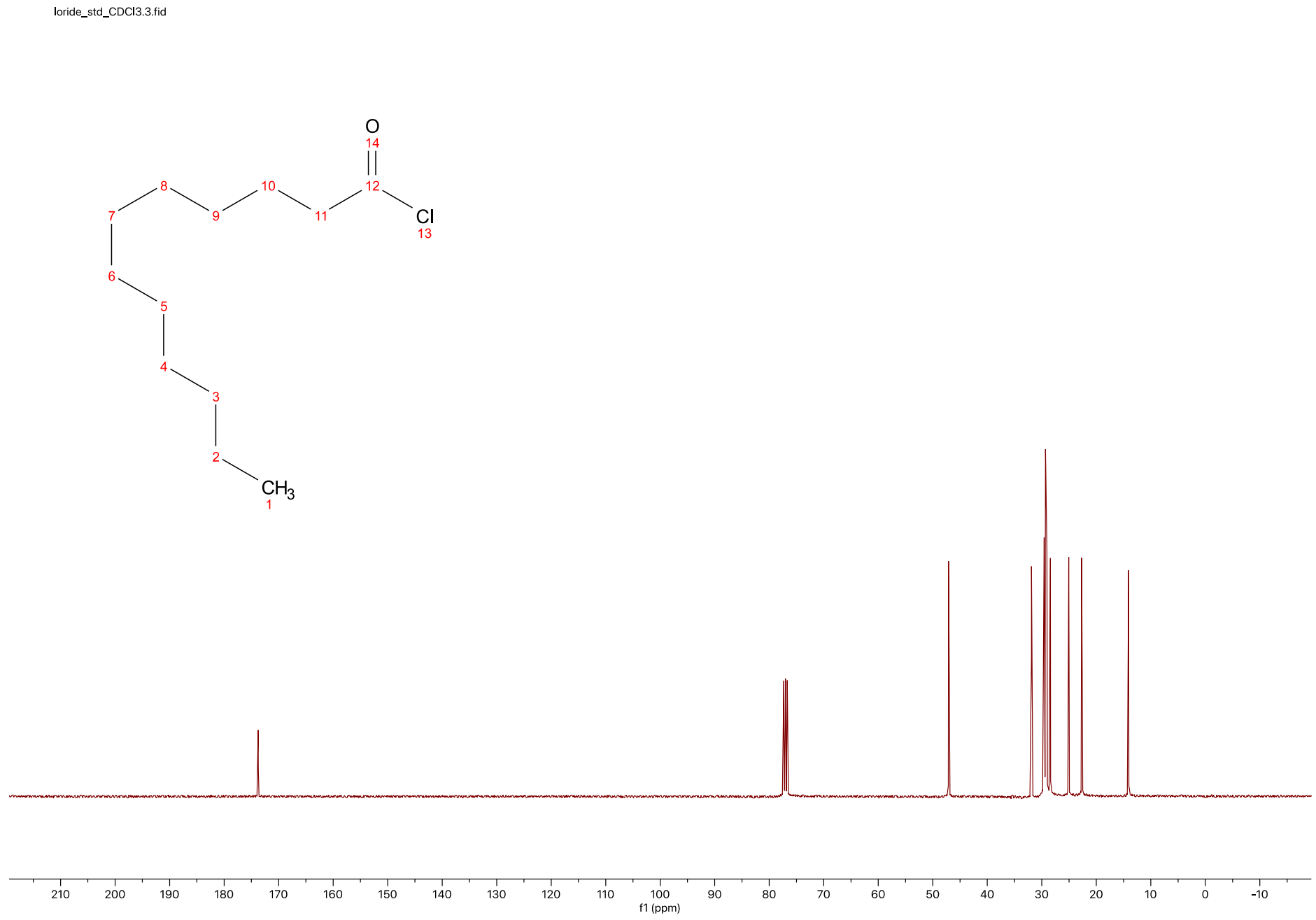

^{13}C NMR (400 MHz, $CDCl_3$) δ (ppm): 173.9 (C12), 47.28 (C11), 32.08 (C3), 25.30 (C10), 22.8 (C2), 14.26 (C1).

IV.6 *N*-(2-Ethylmorpholine)dodecanoate (4)

^{1}H NMR

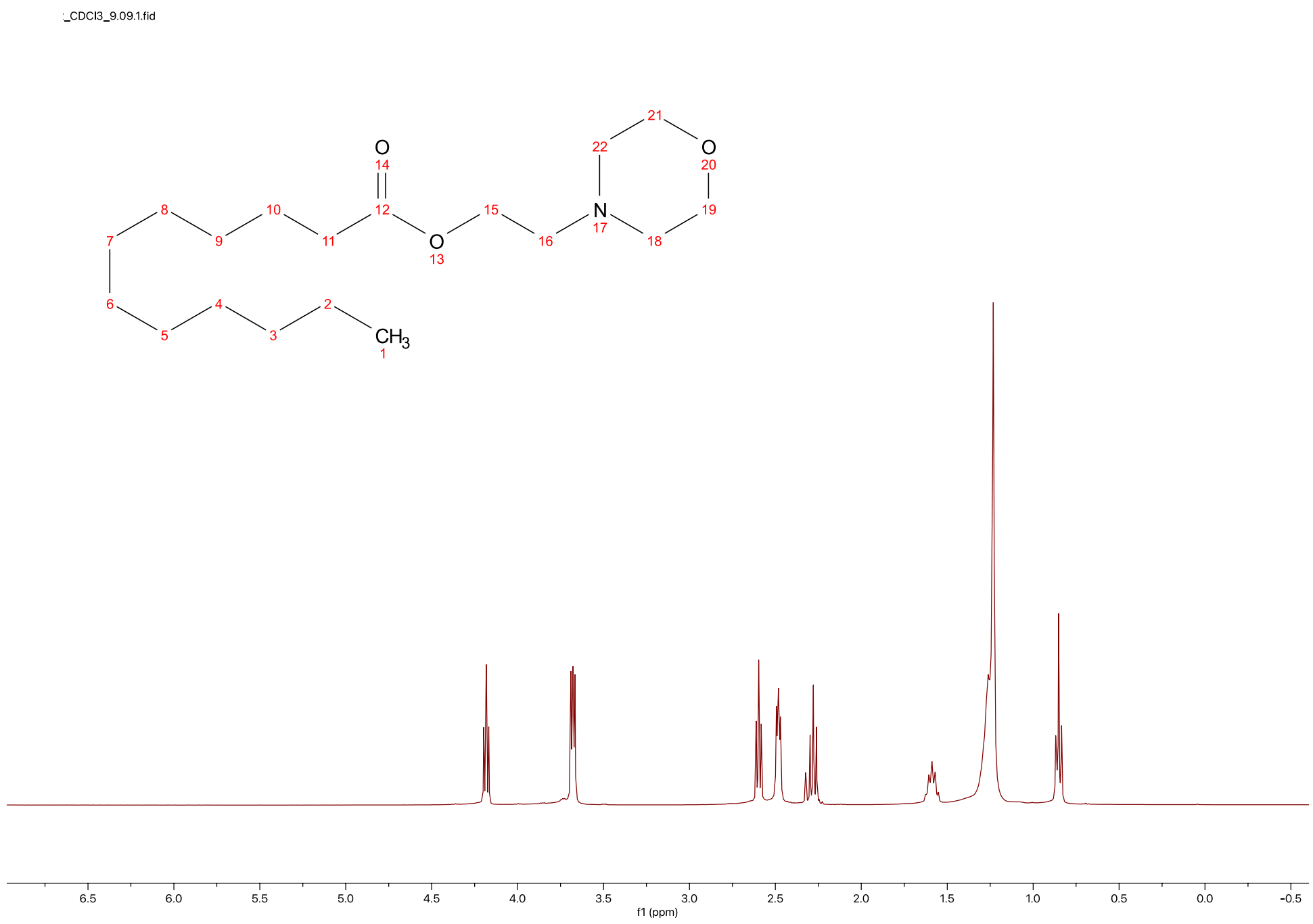

^{1}H NMR (400 MHz, $CDCl_3$) δ (ppm): 4.19 (2H, t, H15), 3.68 (4H, t, H 21-19), 2.60 (2H, t, H16), 2.48 (4H, m, H 22-18), 2.28 (2H, t, H11), 1.60 (2H, m, H10), 1.24 (16H, s, H 2-9), 0.88 (3H, t, H1).

^{13}C NMR

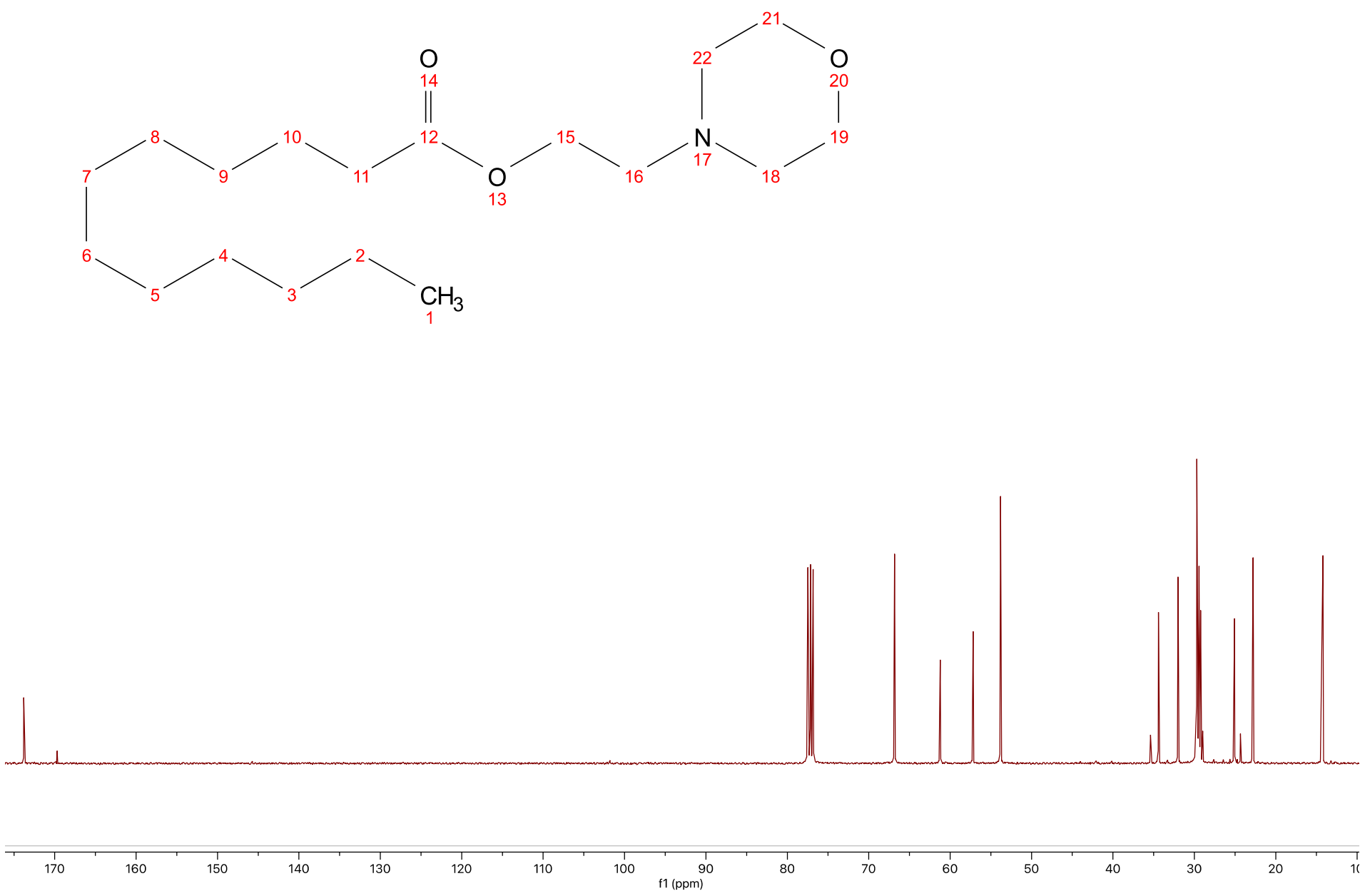

^{13}C NMR (400 MHz, $CDCl_3$) δ (ppm): 173.77 (C12), 66.65 (C21-C19), 61.22 (C15), 57.12 (C16), 53.64 (C22-C18), 34.39 (C11), 32.01 (C9), 29.70 (C3-C8), 25.05 (C10), 22.78 (C2), 14.19 (C1).

IV.7 Ethyl piperidine decanoate (5)

^{1}H NMR

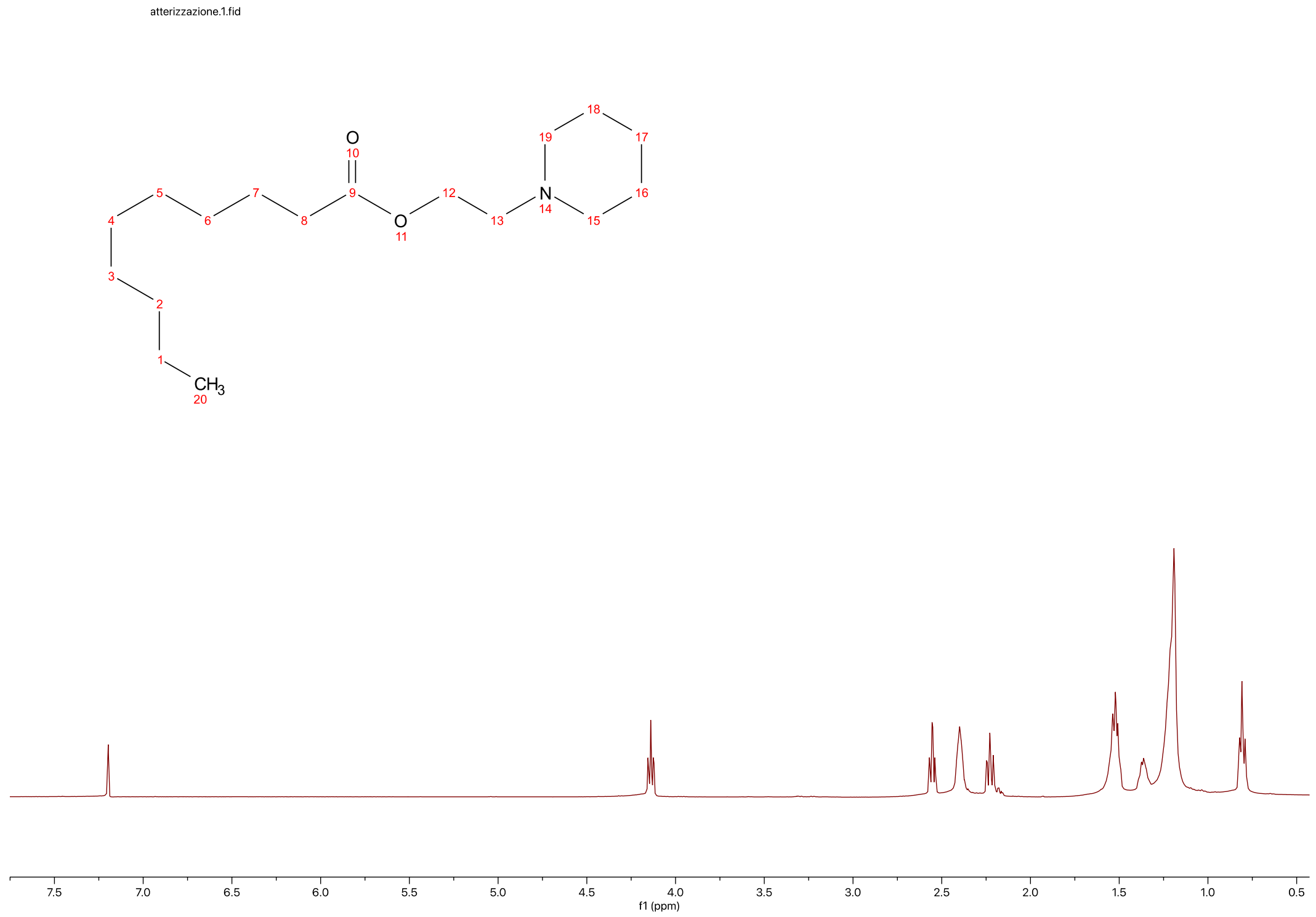

^{1}H NMR (400 MHz, $CDCl_3$) δ (ppm): 4.21 (2H, t, H12), 2.63 (2H, t, H13), 2.48 (4H, H 15-19), 2.29 (2H, t, H8), 1.60 (6H, m, H16-17-18), 1.43 (2H, t, H7), 1.25 (16H, s, H1-6), 0.87 (3H, t, H20).

^{13}C NMR

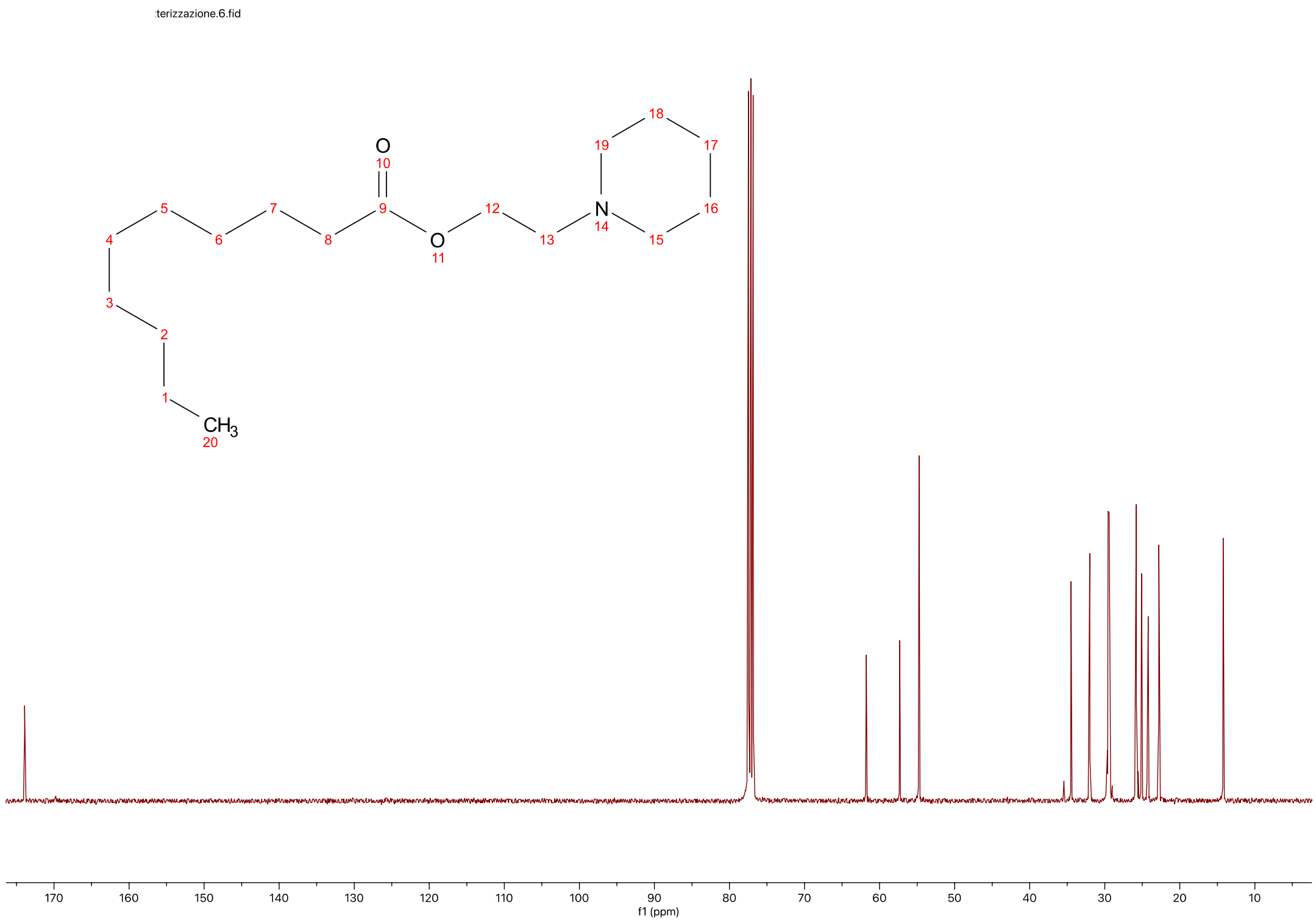

^{13}C NMR (400 MHz, $CDCl_3$) δ (ppm): 173.91 (C9), 61.76 (C12), 57.34 (C13), 54.76 (C19-15), 34.52 (C8), 32.06 (C2), 29.52 (C3-7) 25.31 (C 16-17-18), 24.30 (C7), 22.8 (C1), 14.28 (C20).

IV.8 Ethyl piperidine laurate (6)

^{1}H NMR

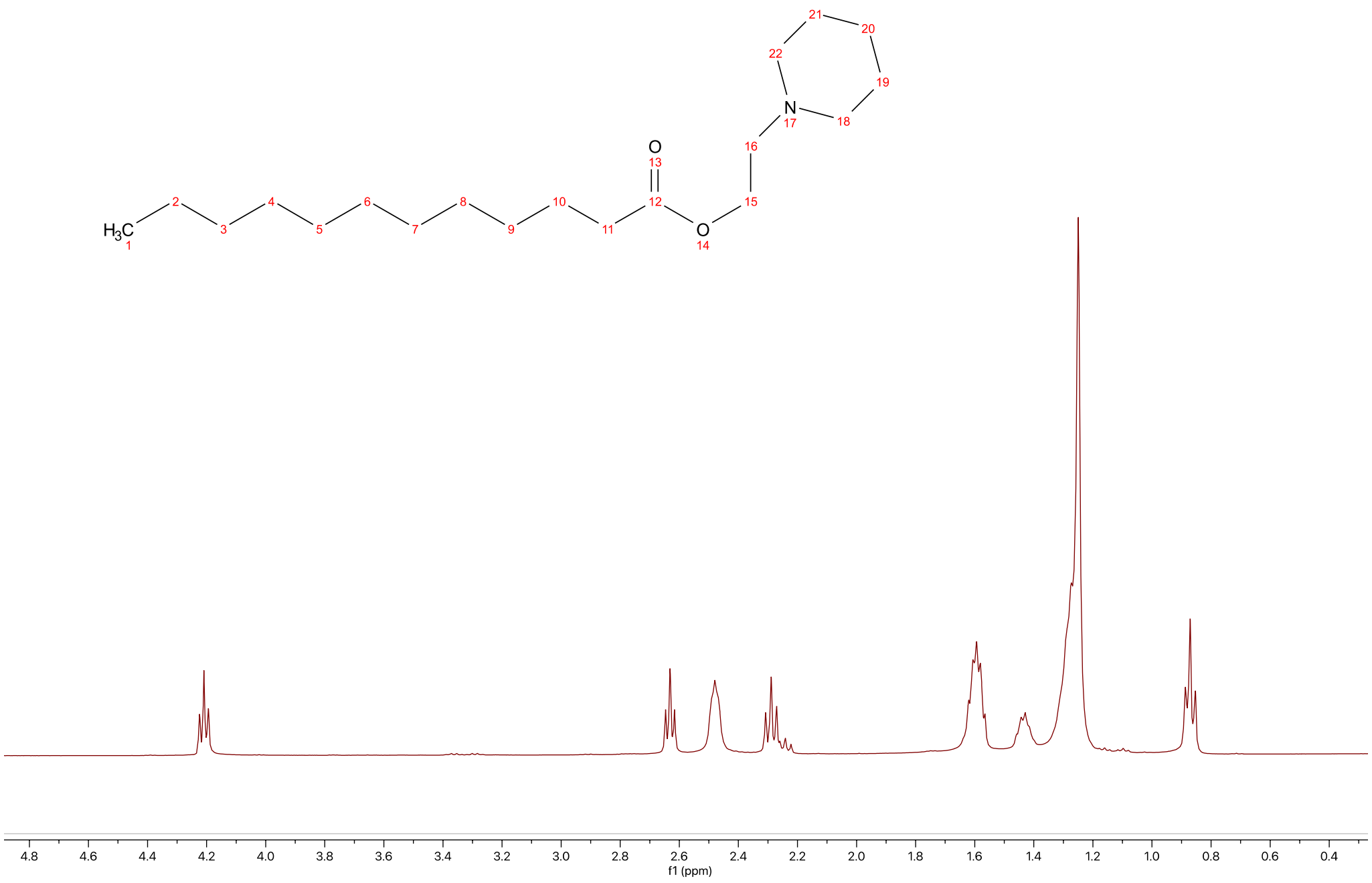

^{1}H NMR (400 MHz, $CDCl_3$) δ (ppm): 4.21 (2H, t, H15), 2.63 (2H, t, H16), 2.48 (4H, H 18-22), 2.29 (2H, t, H11), 1.60 (6H, m, H19-20-21), 1.43 (2H, t, H10), 1.25 (16H, s, H2-9), 0.87 (3H, t, H1).

^{13}C NMR

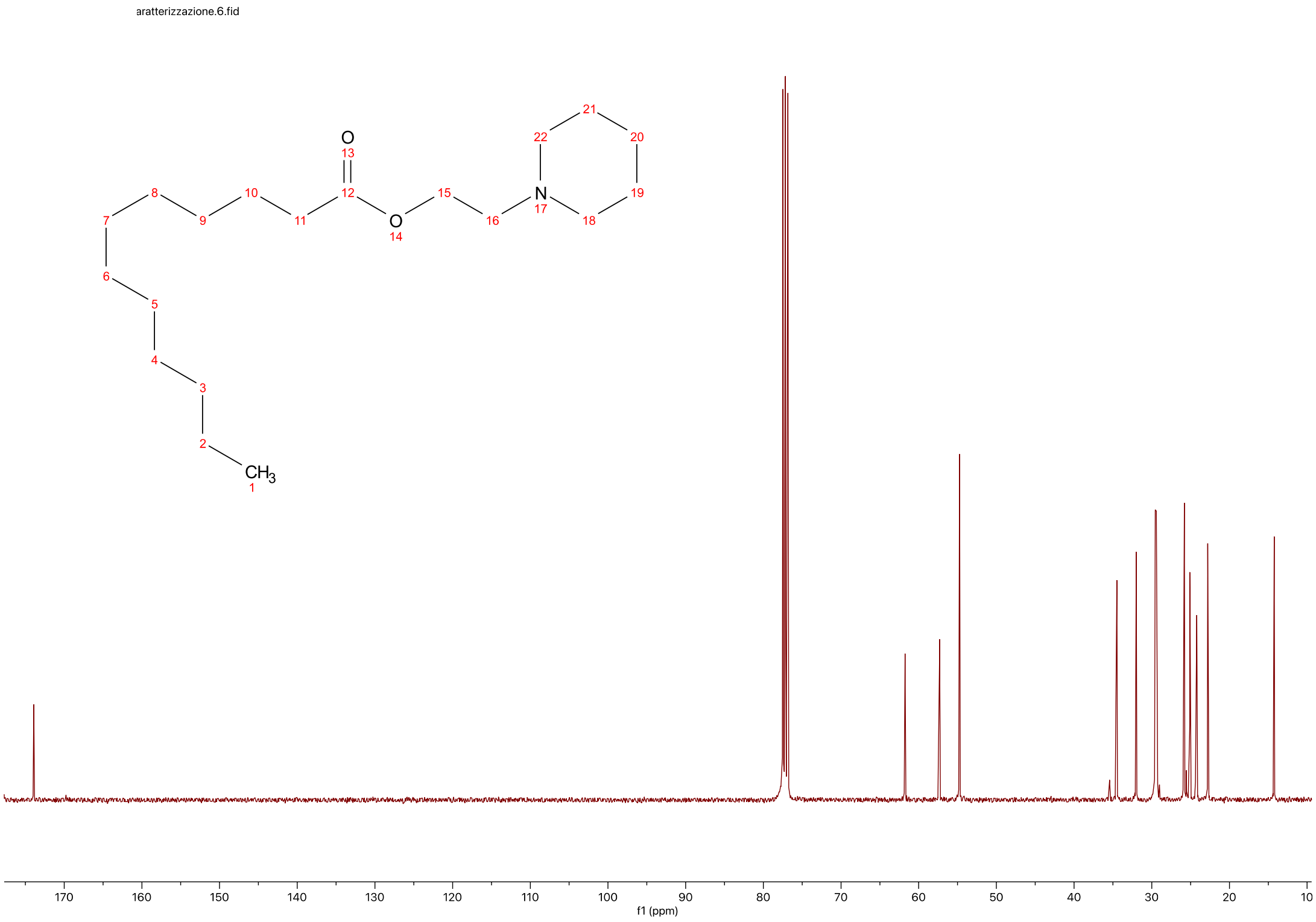

^{13}C NMR (400 MHz, $CDCl_3$) δ (ppm): 174.05 (C12), 61.76 (C15), 57.34 (C16), 54.76 (C18-22), 34.52 (C11), 32.06 (C3), 29.52 (C4-9) 25.31 (C 19-20-21), 24.30 (C10), 22.8 (C2), 14.28 (C1).

IV.9 2-chloro-4,6-(*N*-(3-propyl imidazole)decanamide)-1,3,5-triazine (7)

^{1}H NMR

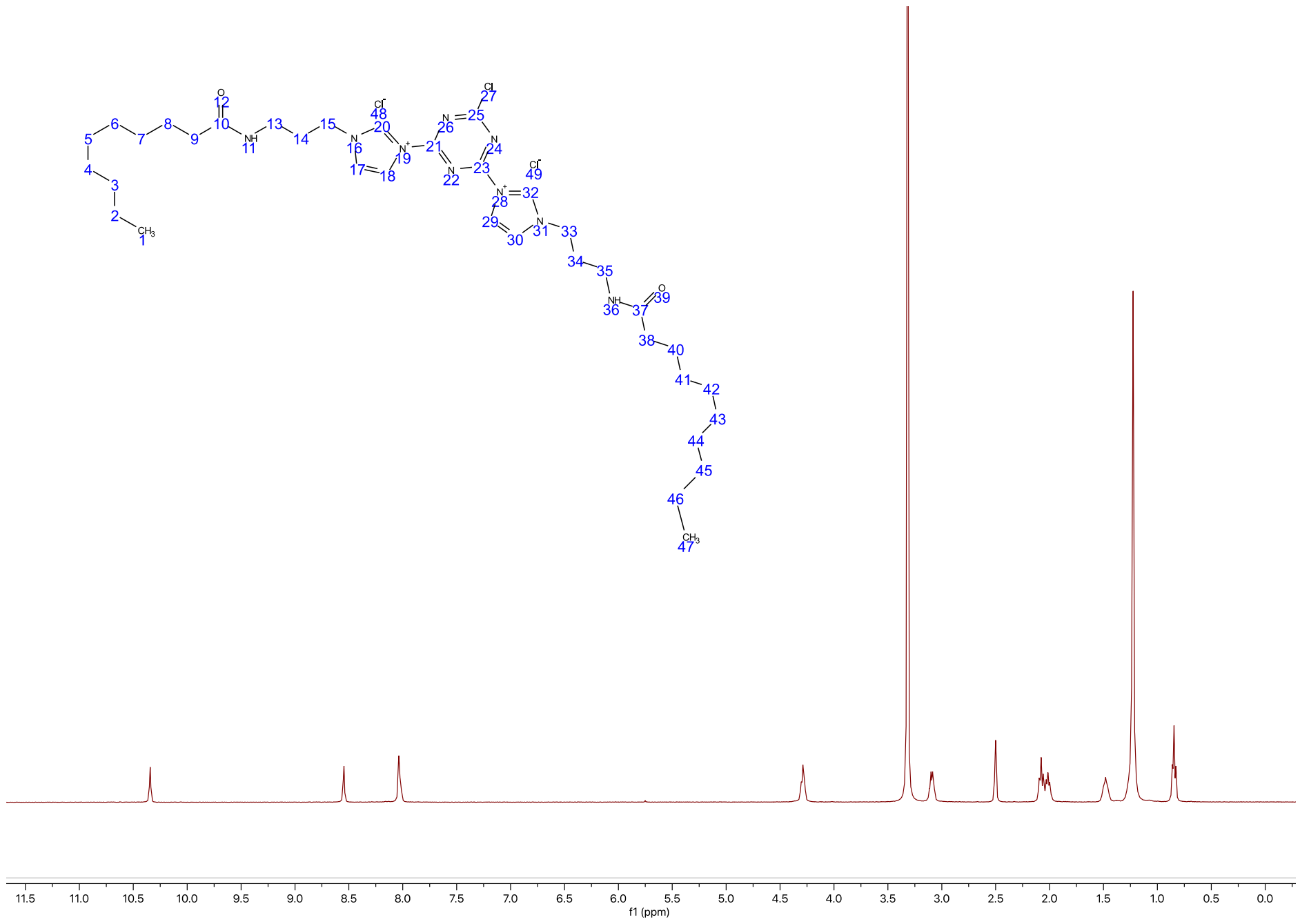

^{1}H NMR (400 MHz, DMSO-d_6) δ (ppm): 10.37 (2H, s, H20 e H32), 8.54 (2H, s, H18 e H30), 8.04 (4H, s, H11, H36, H17 e H29), 4.29 (4H, t, H15 e H33), 3.10 (4H, q, H13 e H35), 2.05 (8H, m, H9, H14, H38 e H34), 1.48 (4H, t, H8 e H40), 1.24 (32H, s, H2-8 e H47-41), 0.87 (6H, t, H1 e H47).

^{13}C NMR

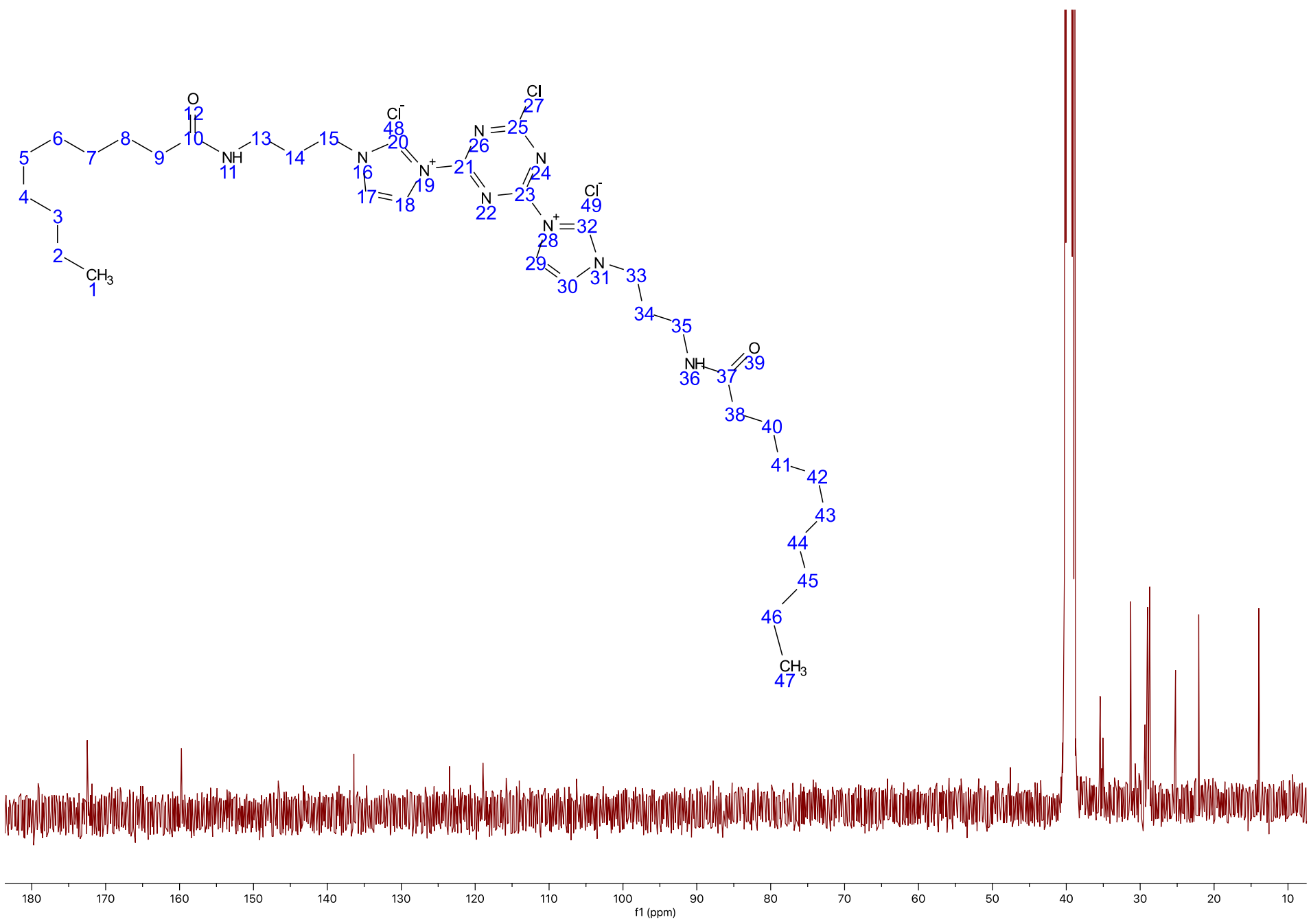

^{13}**C NMR** (400 MHz, DMSO-d$_6$) δ (ppm): 172.53 (C10 e C37), 136.40 (C20-C32), 123.53 (C18 –C30), 119.31 (C17-C29), 47.7 (C15-C33), 35.41 (C9-C14-C38-C34), 35.08 (C13-C35), 31.31 (C7-C41), 28.86 (C2-6, C46-42), 25.24 (C8-C40), 13.94 (C1-C47).

COSY

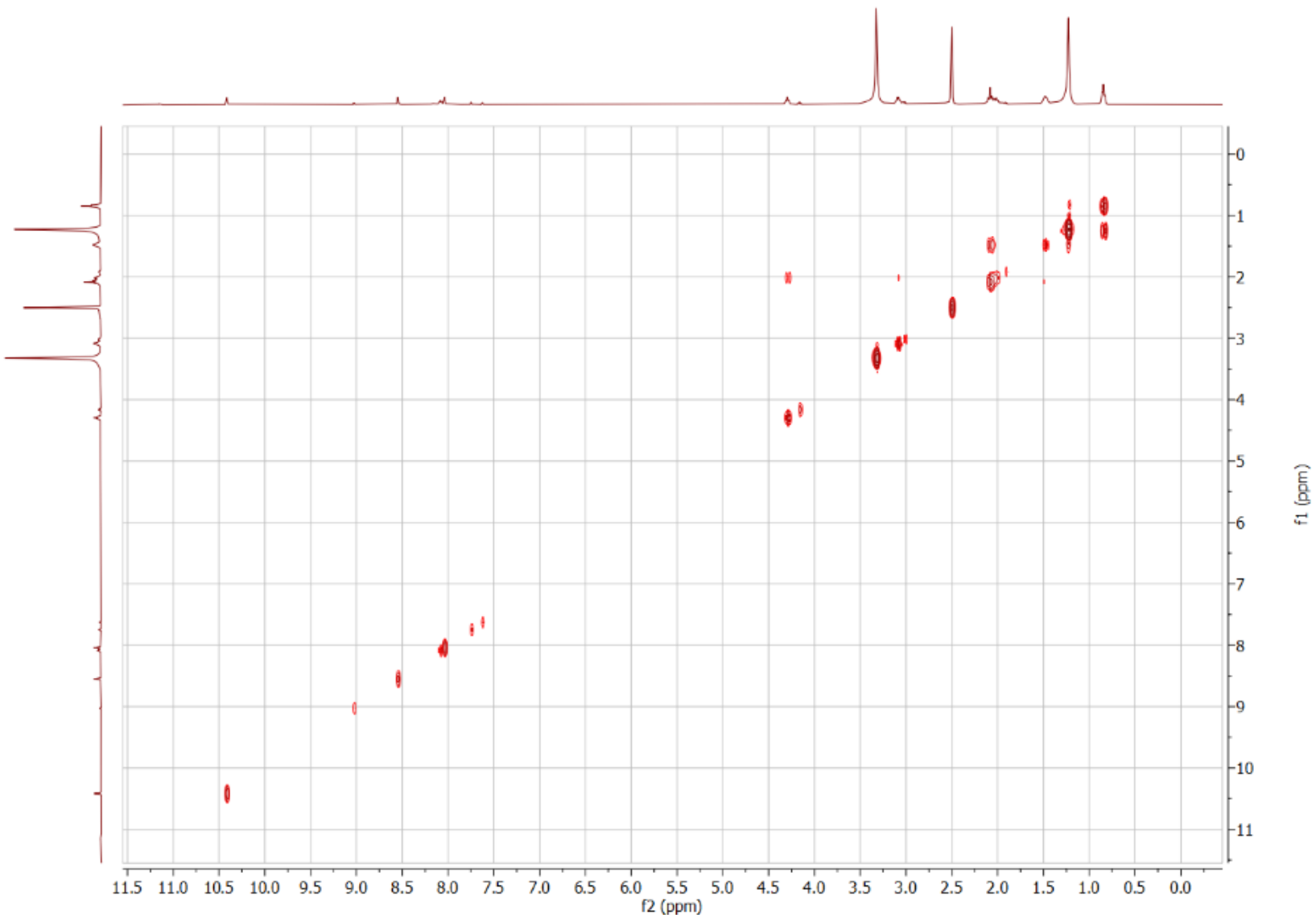

HSQC

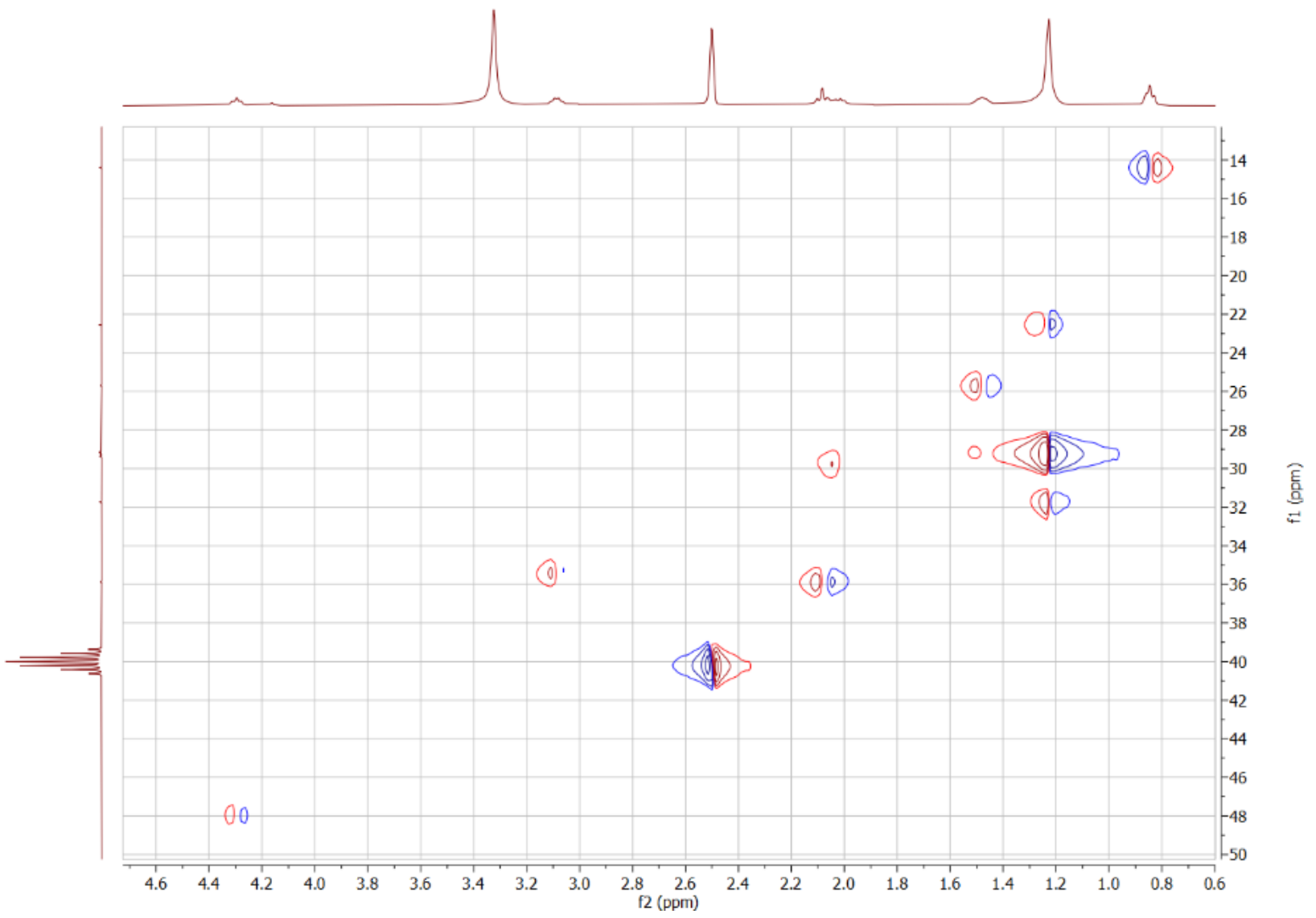

HMQC

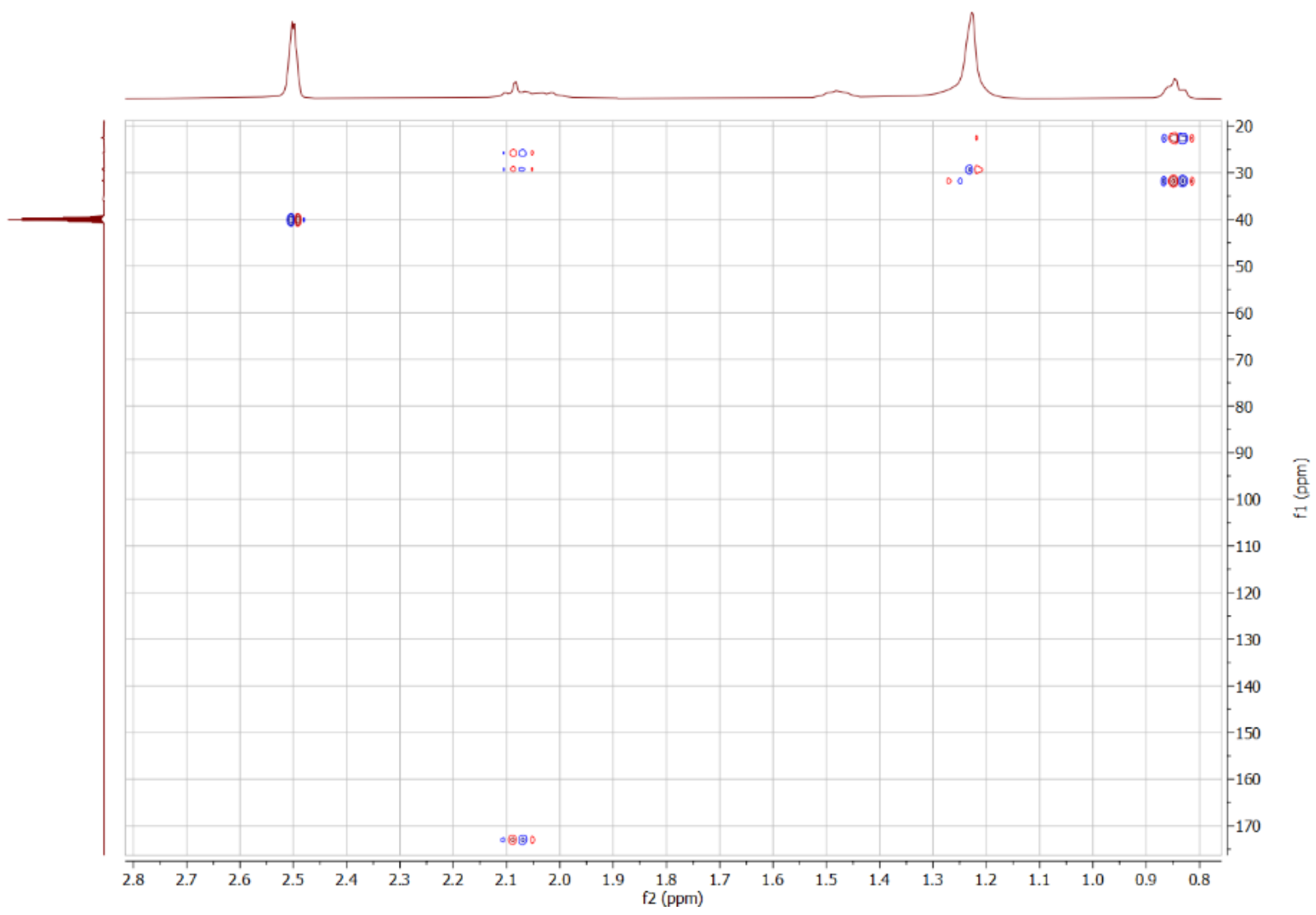

IV.10 2-chloro-4,6-(*N*-(3-propyl imidazole) dodecanamide)-1,3,5-triazine chloride (8)

^{1}H NMR

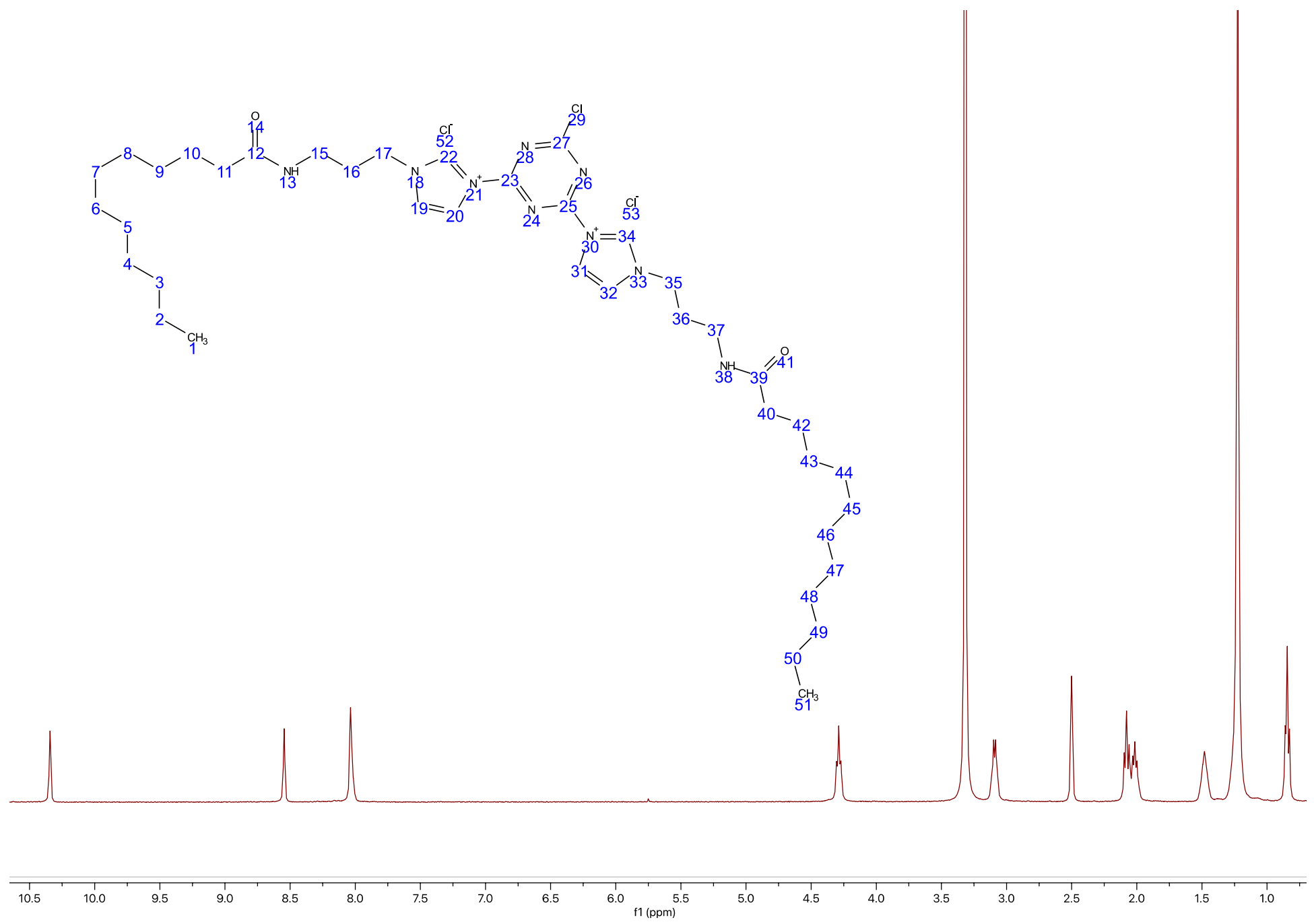

^{1}H NMR (400 MHz, DMSO-d$_6$) δ (ppm): 10.34 (2H, s, H22 e H34), 8.54 (2H, s, H20 e H31), 8.04 (4H, s, H13, H38, H19 e H31), 4.29 (4H, t, H17 e H35), 3.10 (4H, q, H15 e H37), 2.05 (8H, m, H11, H16, H40 e H36), 1.48 (4H, t, H10 e H42), 1.24 (32H, s, H2-9 e H50-43), 0.87 (6H, t, H1 e H51).

^{13}C NMR

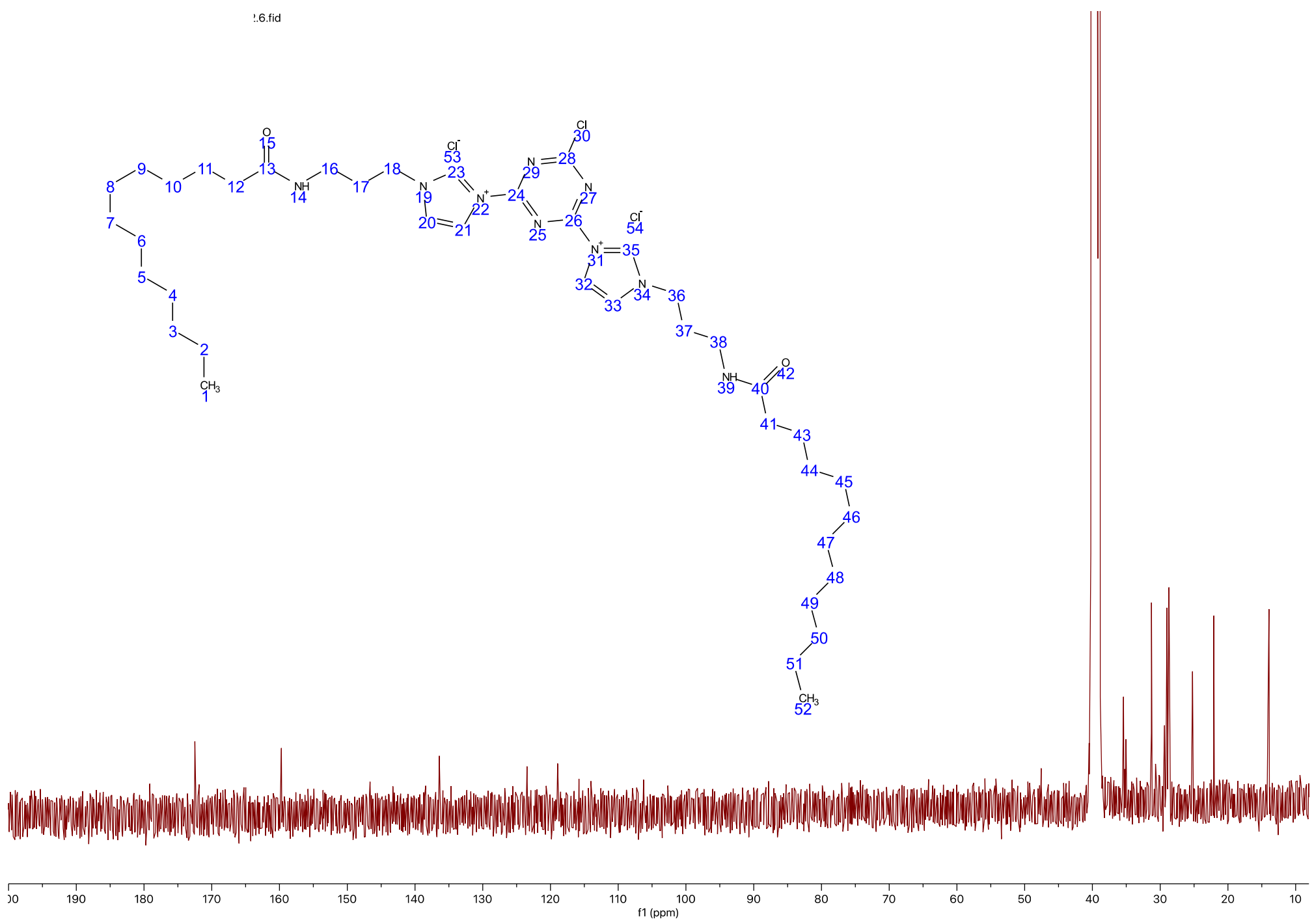

^{13}**C NMR** (400 MHz, DMSO-d_6) δ (ppm): 172.53 (C13 e C40), 136.40 (C23- C35), 123.53 (C21 – C33), 119.31 (C20- C32), 47.7 (C18- C36), 35.41 (C12-C17-C41-C37), 35.08 (C16-C38), 31.31 (C10-C44), 28.86 (C 2-9, C 51-45), 25.24 (C11-C43), 13.94 (C1-C52).

COSY

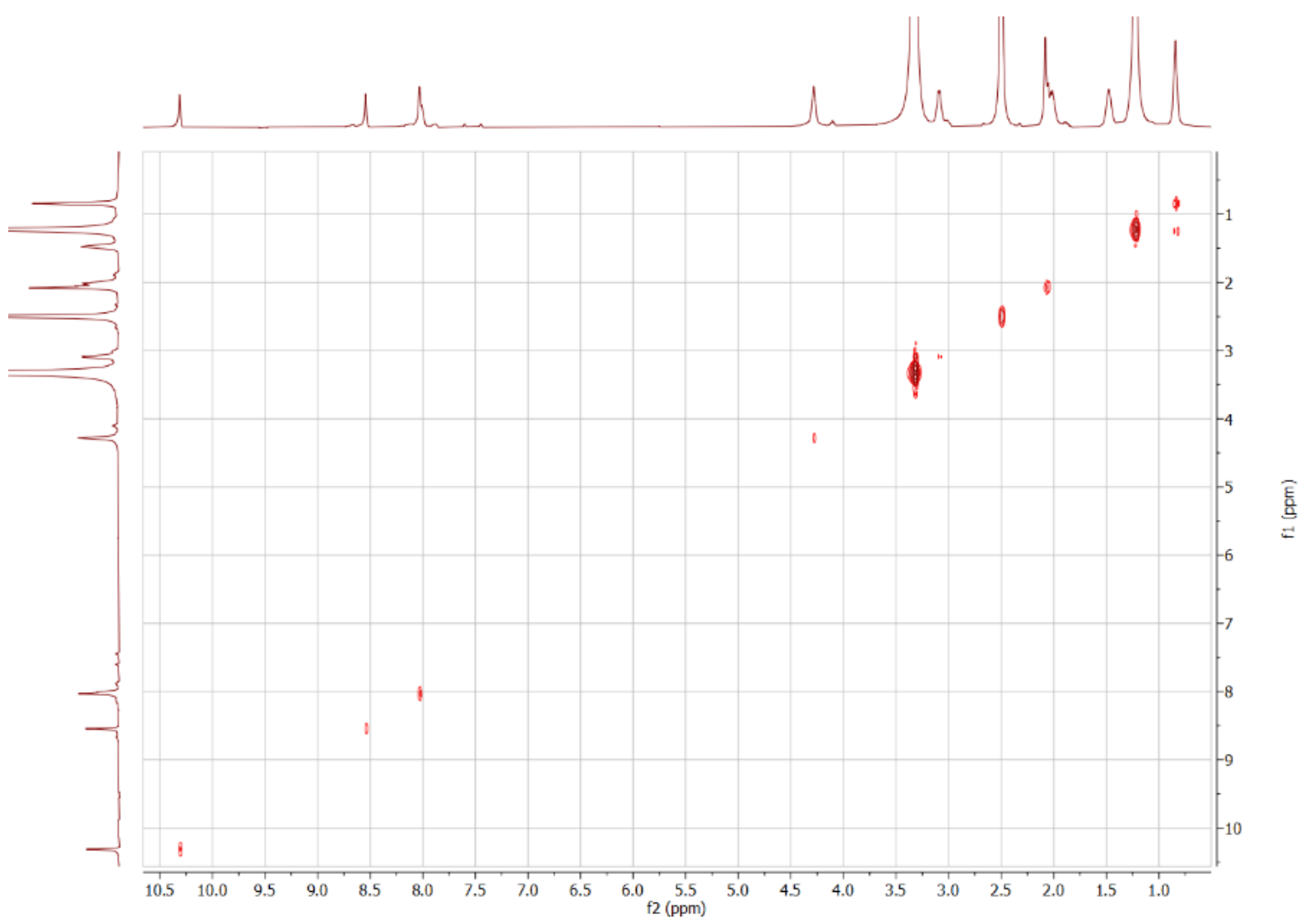

HSQC

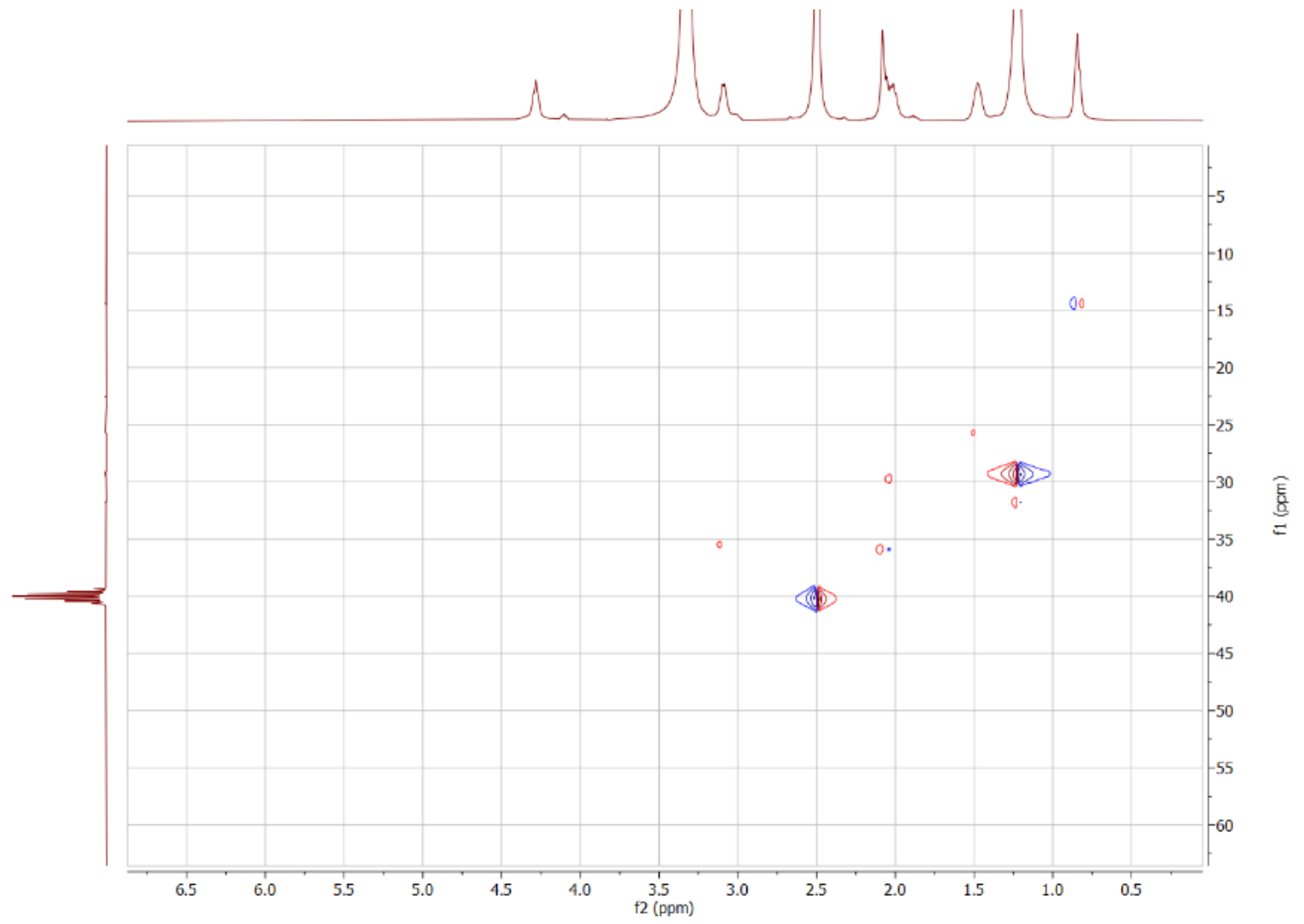

HMQC

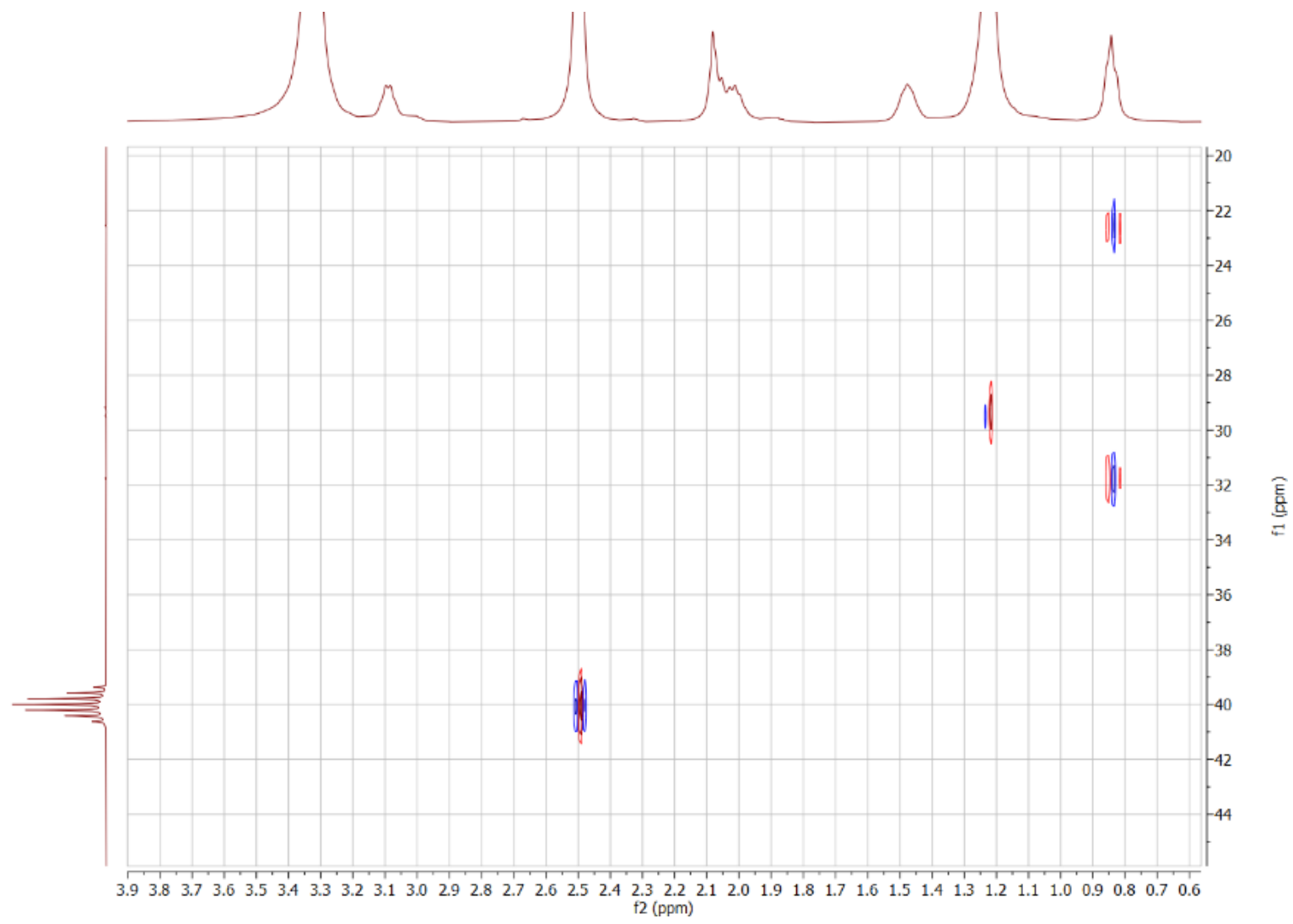

IV.11 2-chloro-4,6-(*N*-(2-ethylmorpholine)decanoate)-1,3,5-triazine chloride (9)

^{1}H NMR

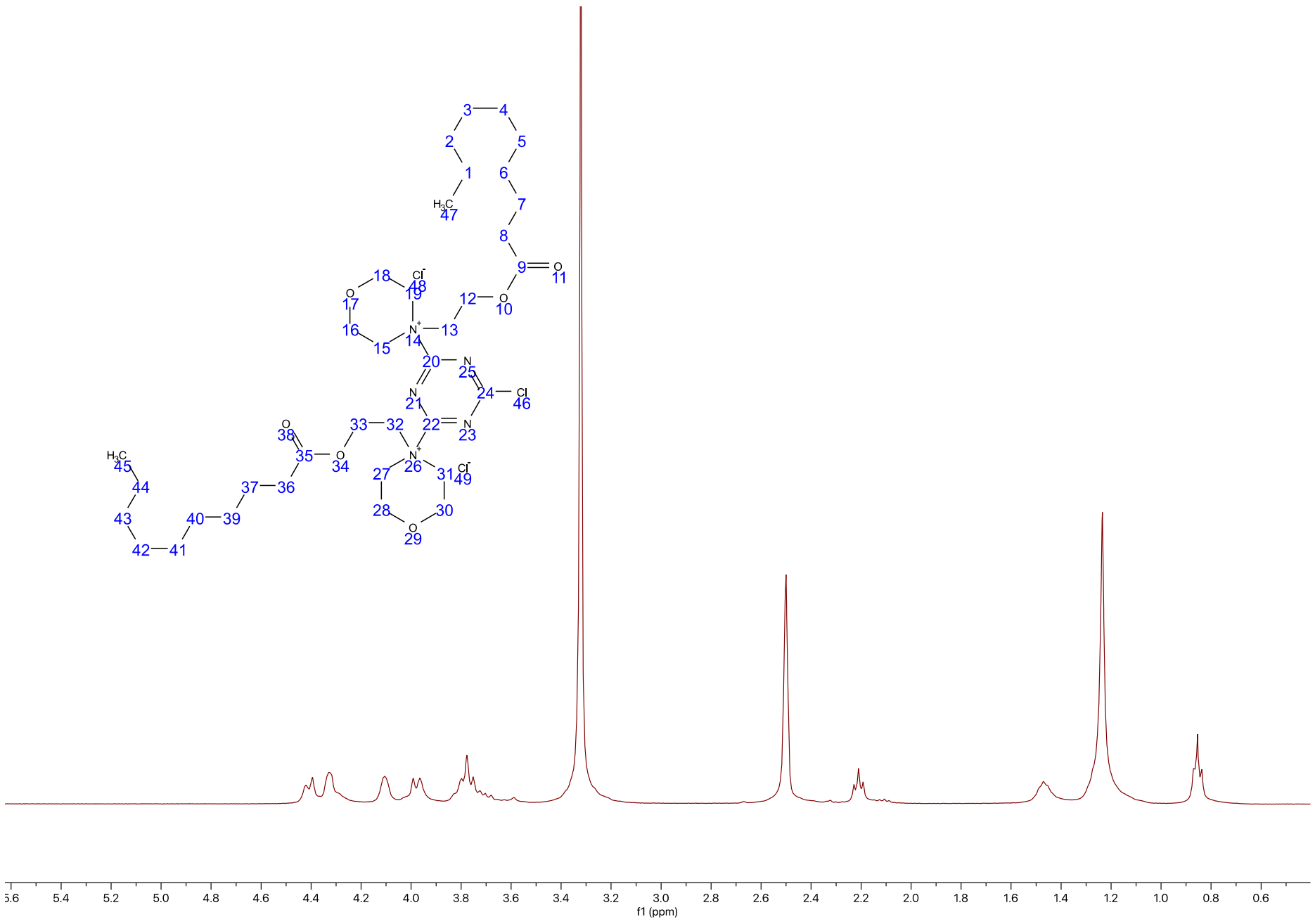

^{1}H NMR (400 MHz, DMSO-d$_6$) δ (ppm): 4.40 (4H, d, H 27-31 e H 15-19), 4.31 (4H, s, H12 e H33), 4.09 (4H, s, H32 e H13), 3.98 (4H, d, H 27-31 e H 15-19), 3.78 (16H, m, H 27-31, H 15-19, H 28-30 e H 16-18), 2.21 (4H, t, H8 e H36), 1.47 (4H, t, H7 e H37), 1.24 (32H, s, H 1-6 e H 44-39), 0.88 (3H, t, H45 e H47).

^{13}C NMR

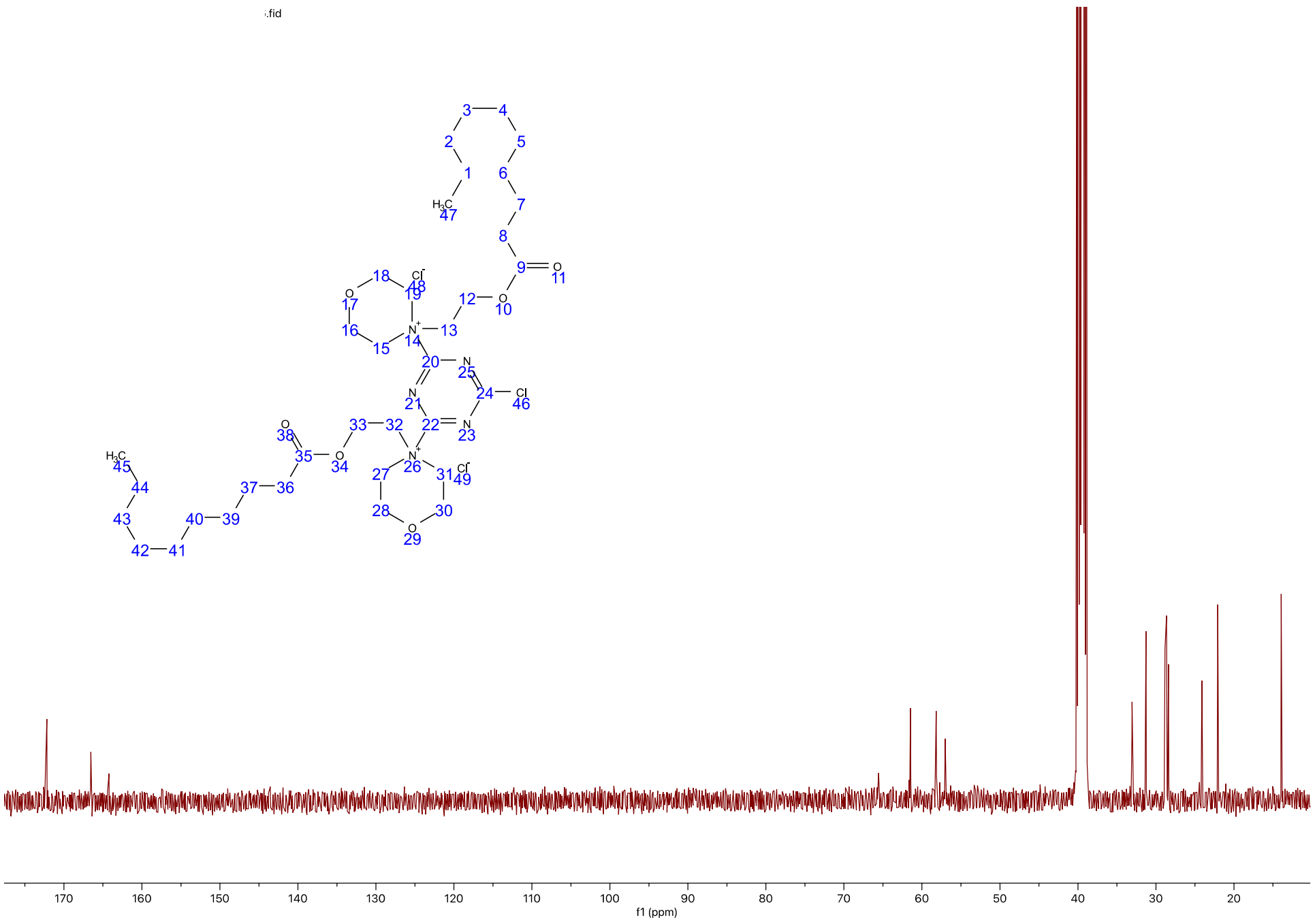

^{13}C NMR (400 MHz, DMSO-d$_6$) δ (ppm): 172.20 (C9 e C35), 165.6 (C20 e C22), 164.19 (C24), 65.45 (C32 e C13), 61.60 (C27-31, C15-19), 58.36 (C 18-16 e C 30-28), 57.14 (C15 e C36), 33.08 (C12 e C33), 31.27 e 28.87 (C1- 8 e C 44-37), 22.08 (C45 e C47).

COSY

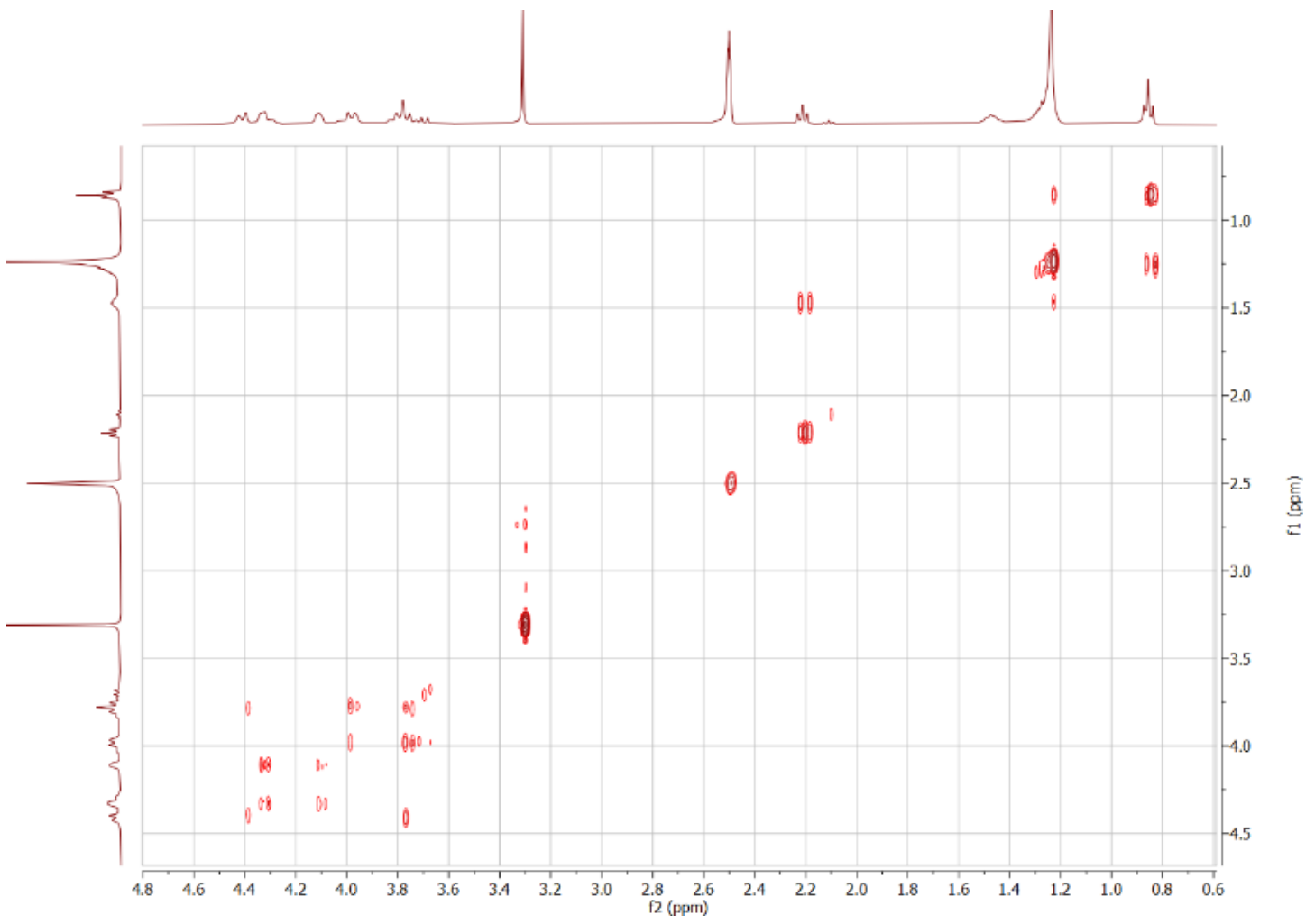

HSQC

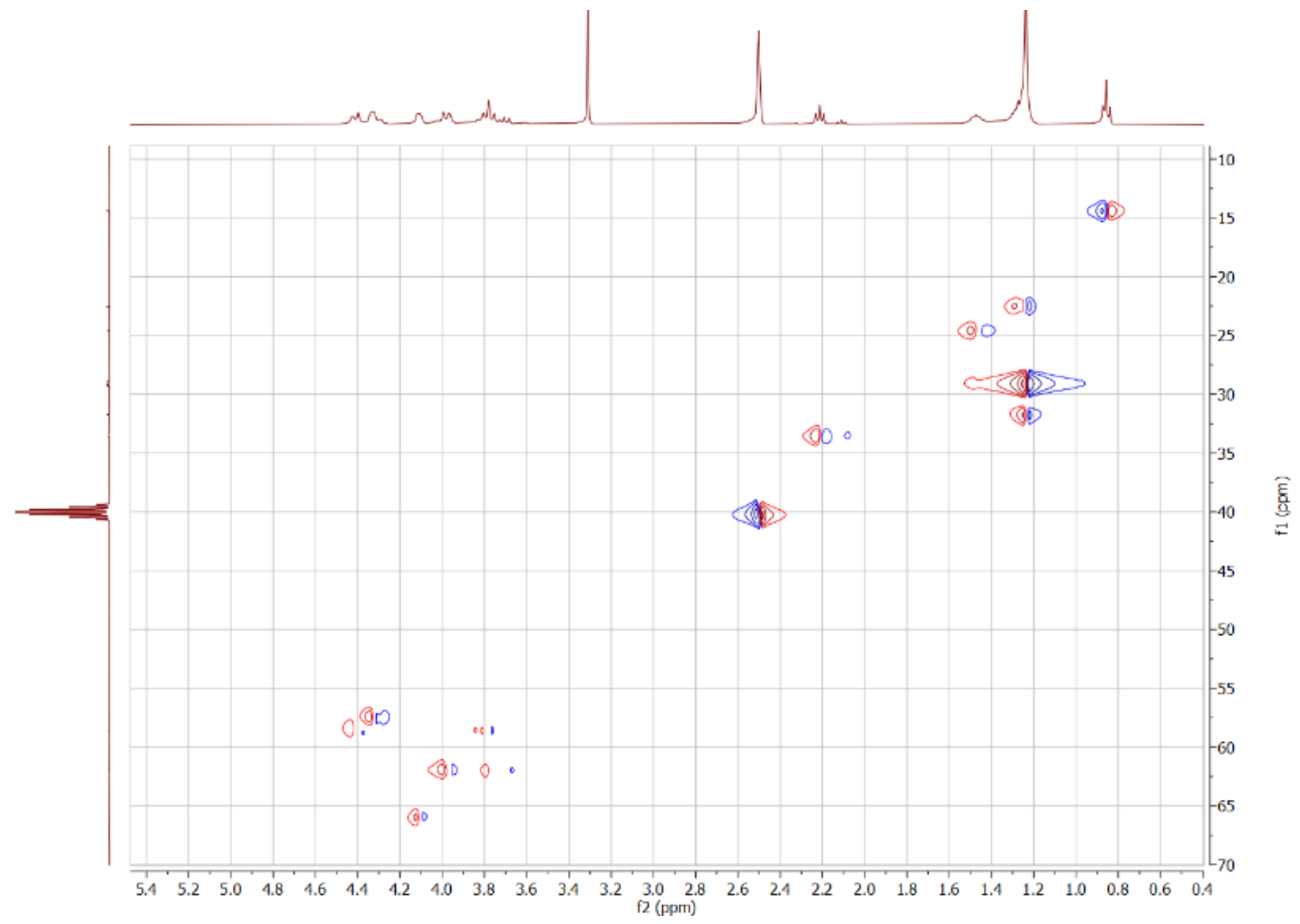

HMQC

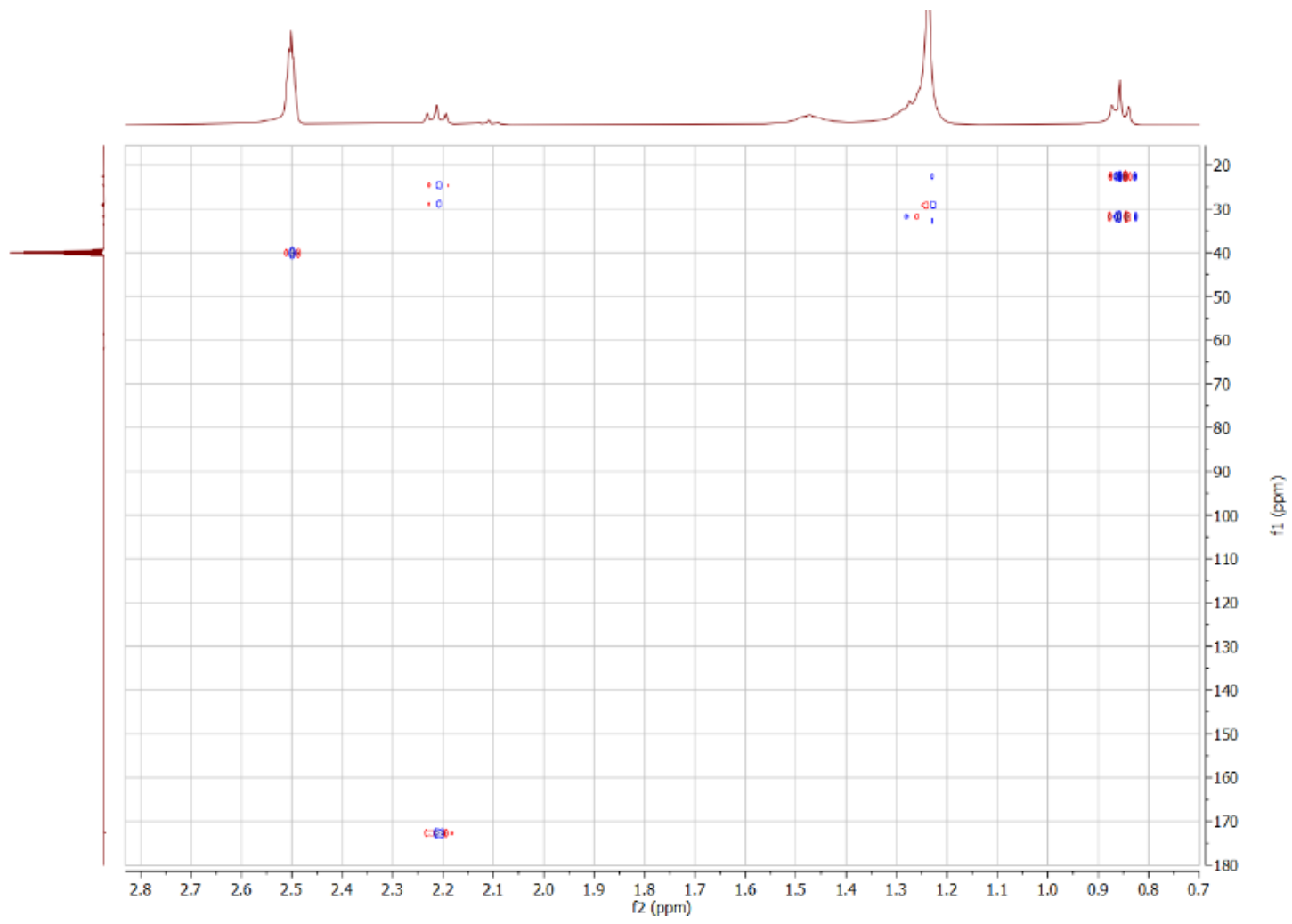

IV.12 2-chloro-4,6-(*N*-(2-ethylmorpholine)dodecanoate)-1,3,5-triazin chloride (10)

^{1}H NMR

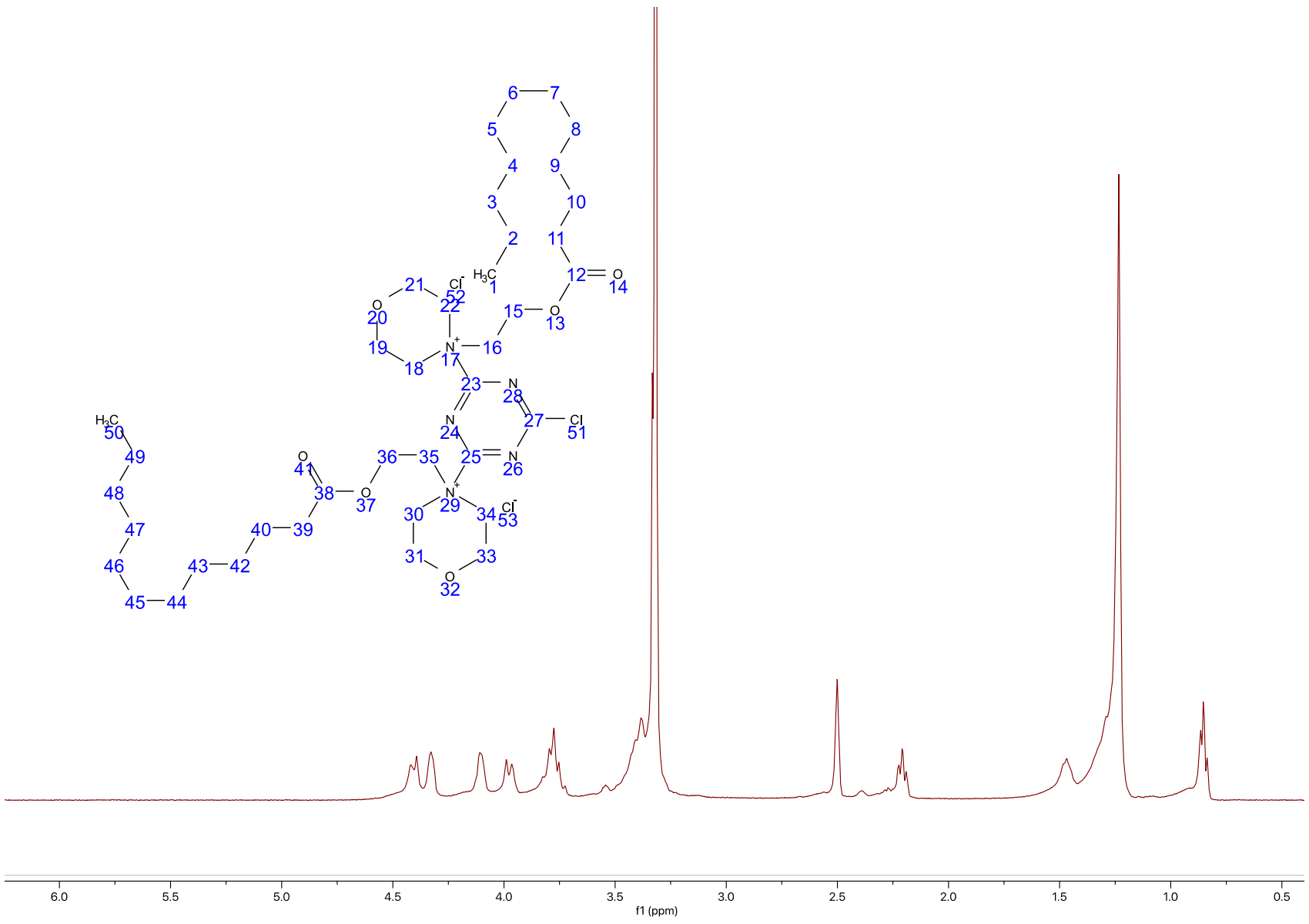

^{1}H NMR (400 MHz, DMSO-d_6) δ (ppm): 4.40 (4H, d, H 30-34 e H 18-22), 4.33 (4H, s, H15 e H36), 4.11 (4H, s, H35 e H16), 3.98 (4H, d, H 30-34 e H 18-22), 3.78 (16H, m, H 30-34, H 18-22, H 21-19 e H 31-33), 2.21 (4H, t, H11 e H39), 1.47 (4H, t, H10 e H40), 1.24 (32H, s, H 2-9 e H 49-42), 0.89 (6H, t, H50 e H1).

^{13}C NMR

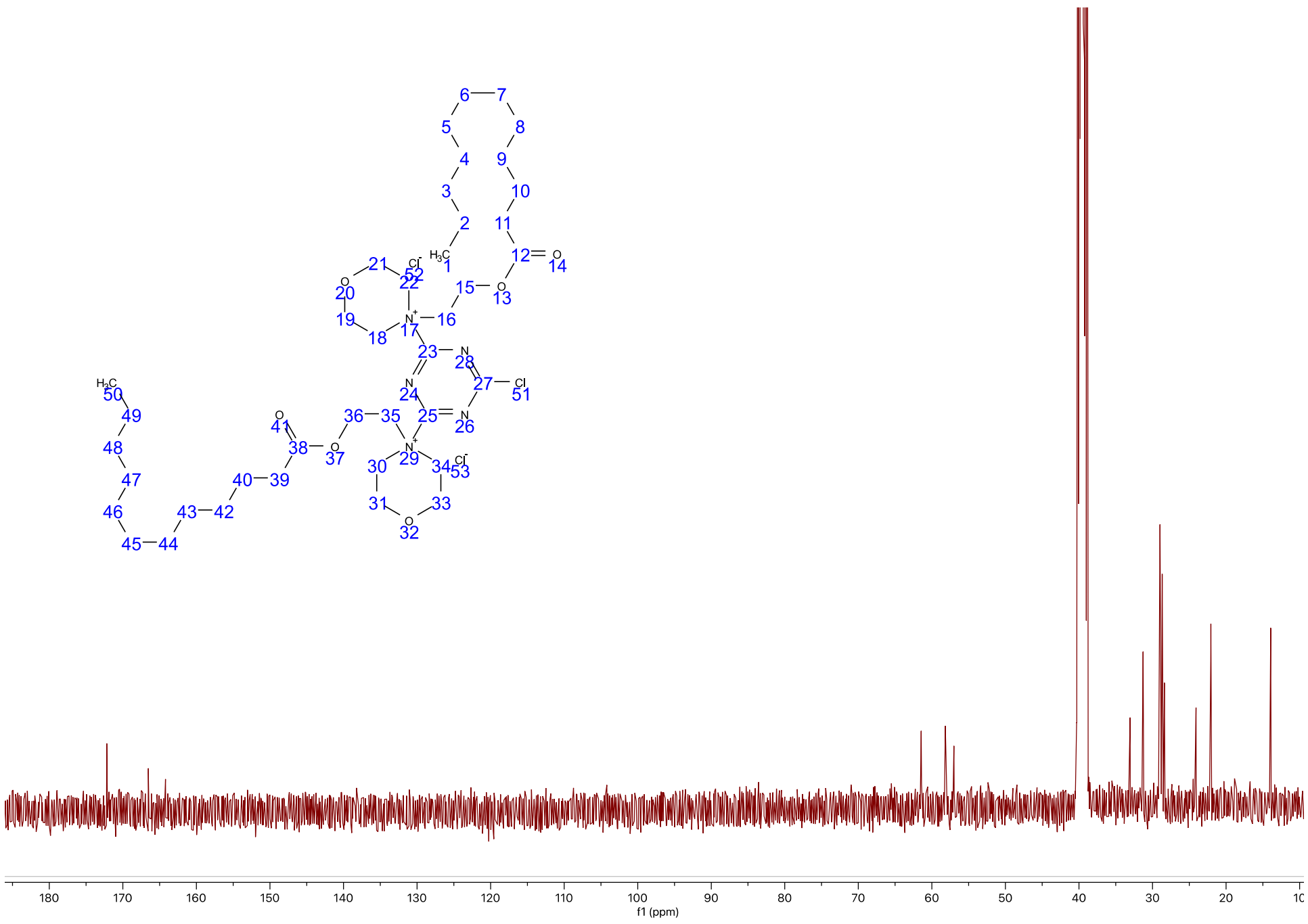

^{13}C NMR (400 MHz, DMSO-d_6) δ (ppm): 172.25 (C12 e C38), 166.6 (C23 e C25), 164.21 (C27), 65.60 (C35 e C16), 61.60 (C30-34, C18-22, C 21-19 e C 31-33), 58.36 (C30-34, C18-22, C 21-19 e C 31-33), 57.14 (C15 e C36), 33.08 (C11 e C39), 31.27 e 28.87 (C 3- 10 e C 48-42), 22.08 (C40 e C10).

COSY

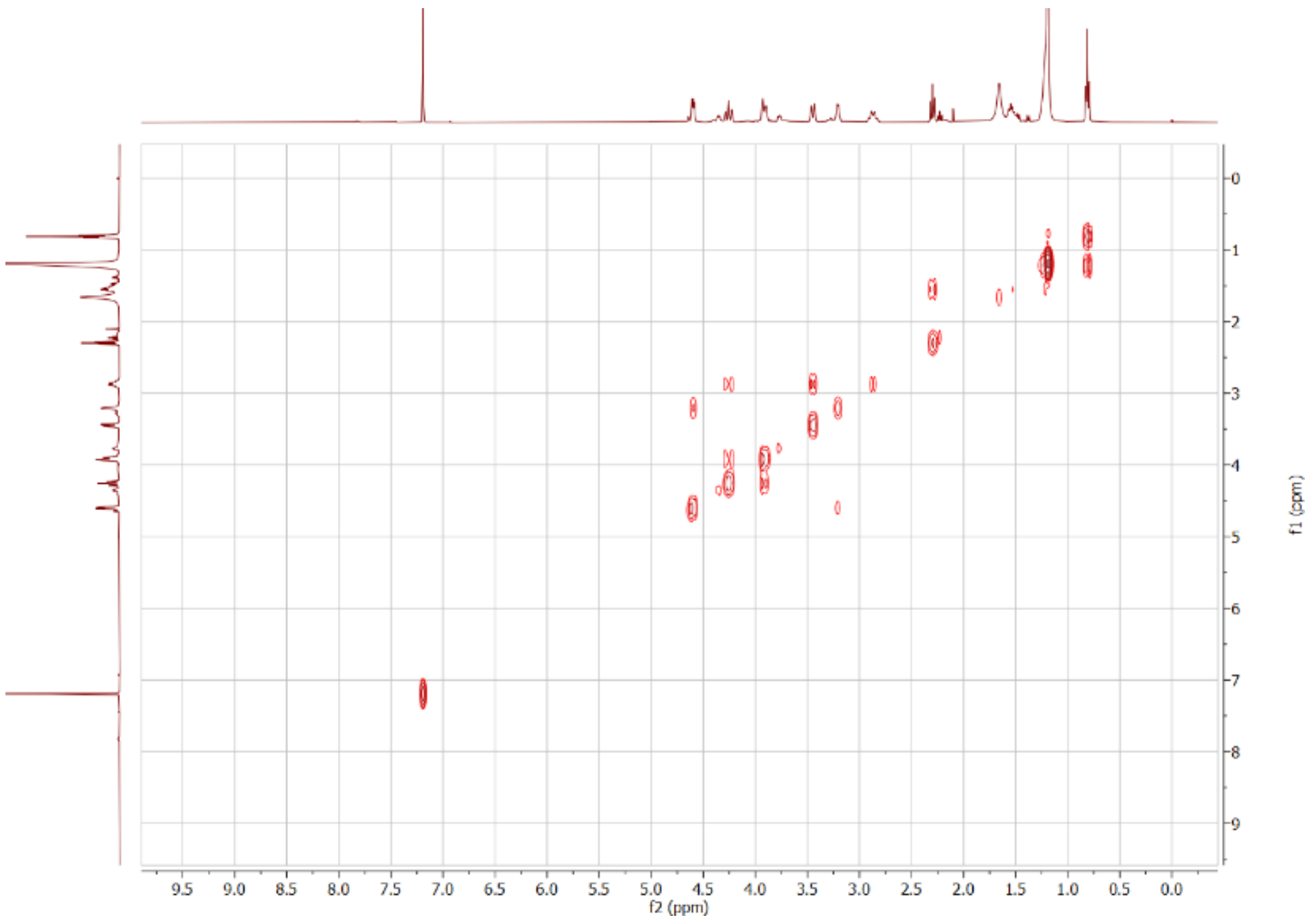

HSQC

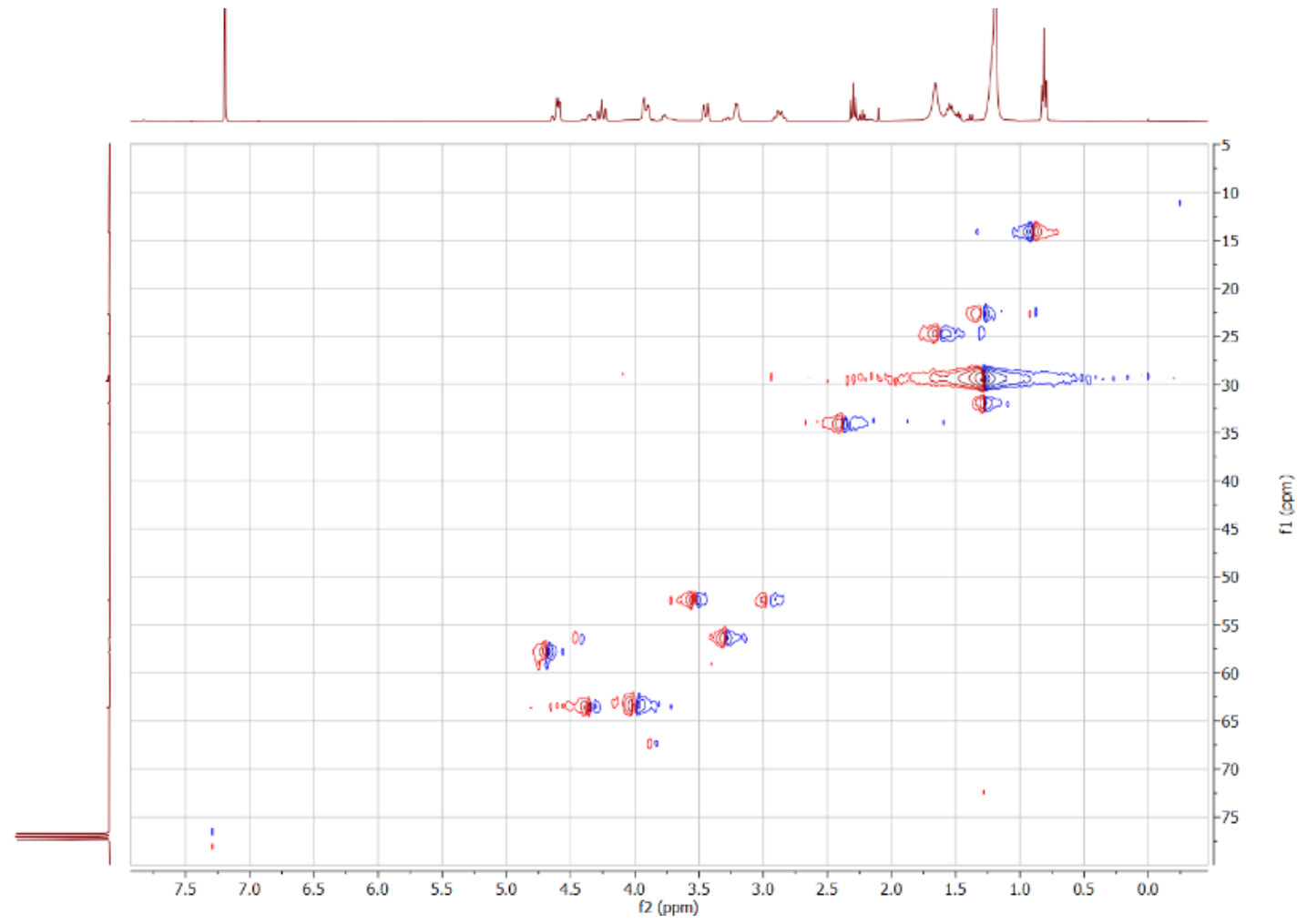

HMQC

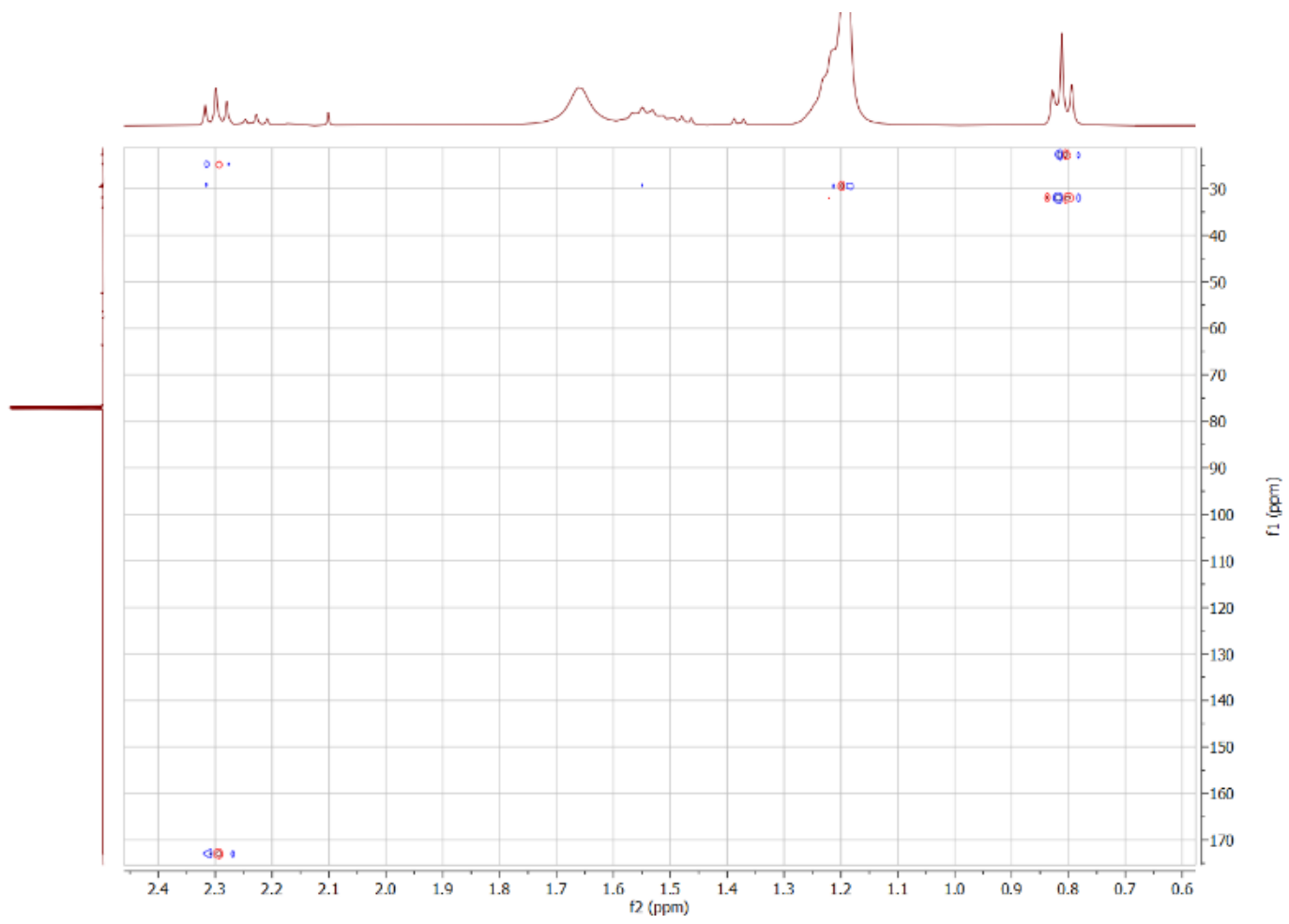

IV.13 2-chloro-4,6-(*N*-(ethylpiperidine)decanoate)-1,3,5-triazin chloride (11)

^{1}H NMR

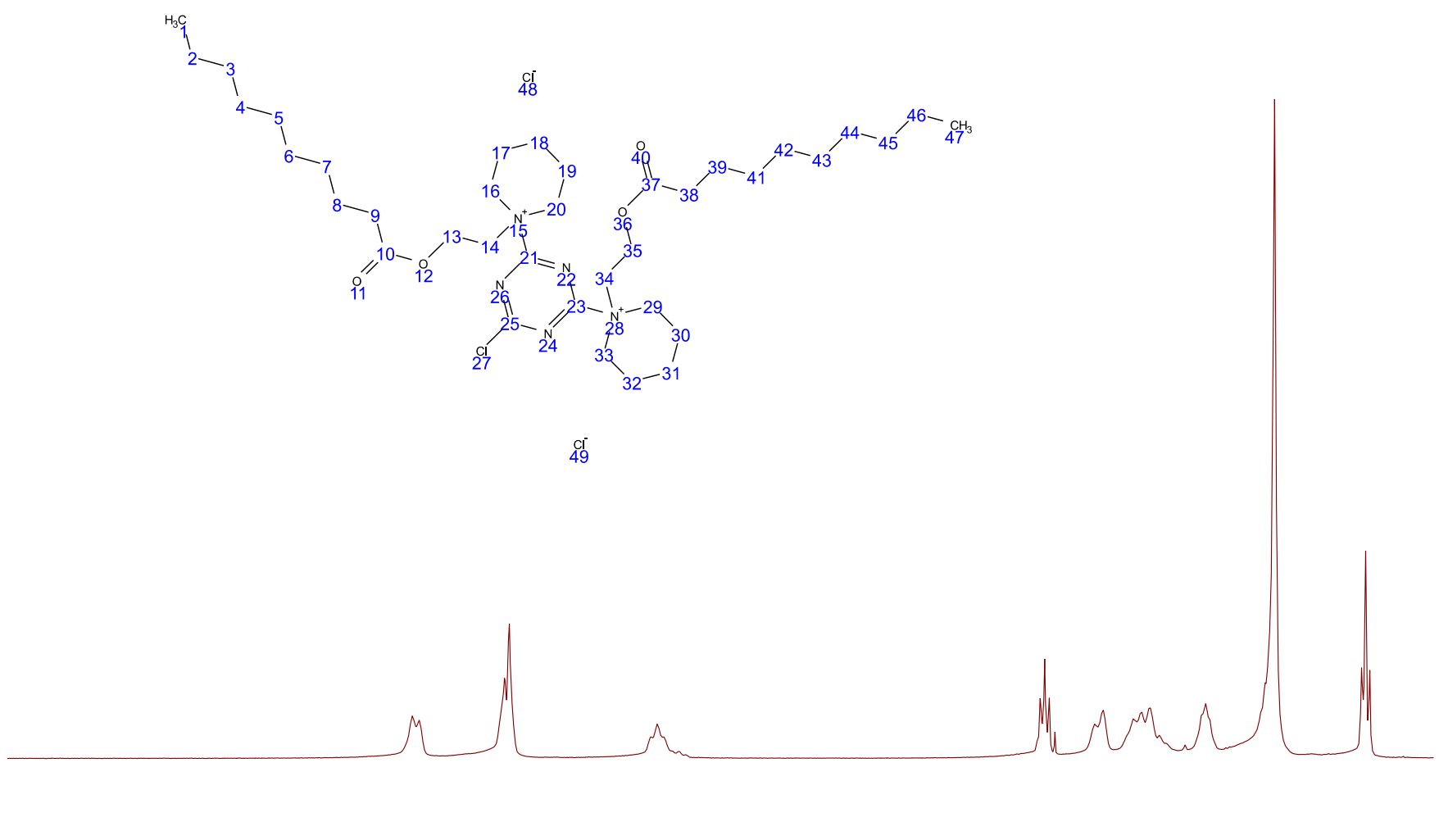

^{1}H NMR (400 MHz, $CDCl_3$) δ (ppm): 4.84 (4H, s, H 16-20 e H 33-29), 4.43 (8H, H 13-14 e H 35-34), 3.82 (4H, t, H 16-20 e H 33-29), 2.21 (4H, t, H9 e H38), 1.89 (4H, H18 e H31), 1.77 (8H, m, H 17-19 e H 32-30), 1.54 (4H, t, H8 e H39), 1.25 (C 2-7 e C 46-41), 0.88 (C1 e C47).

^{13}C NMR

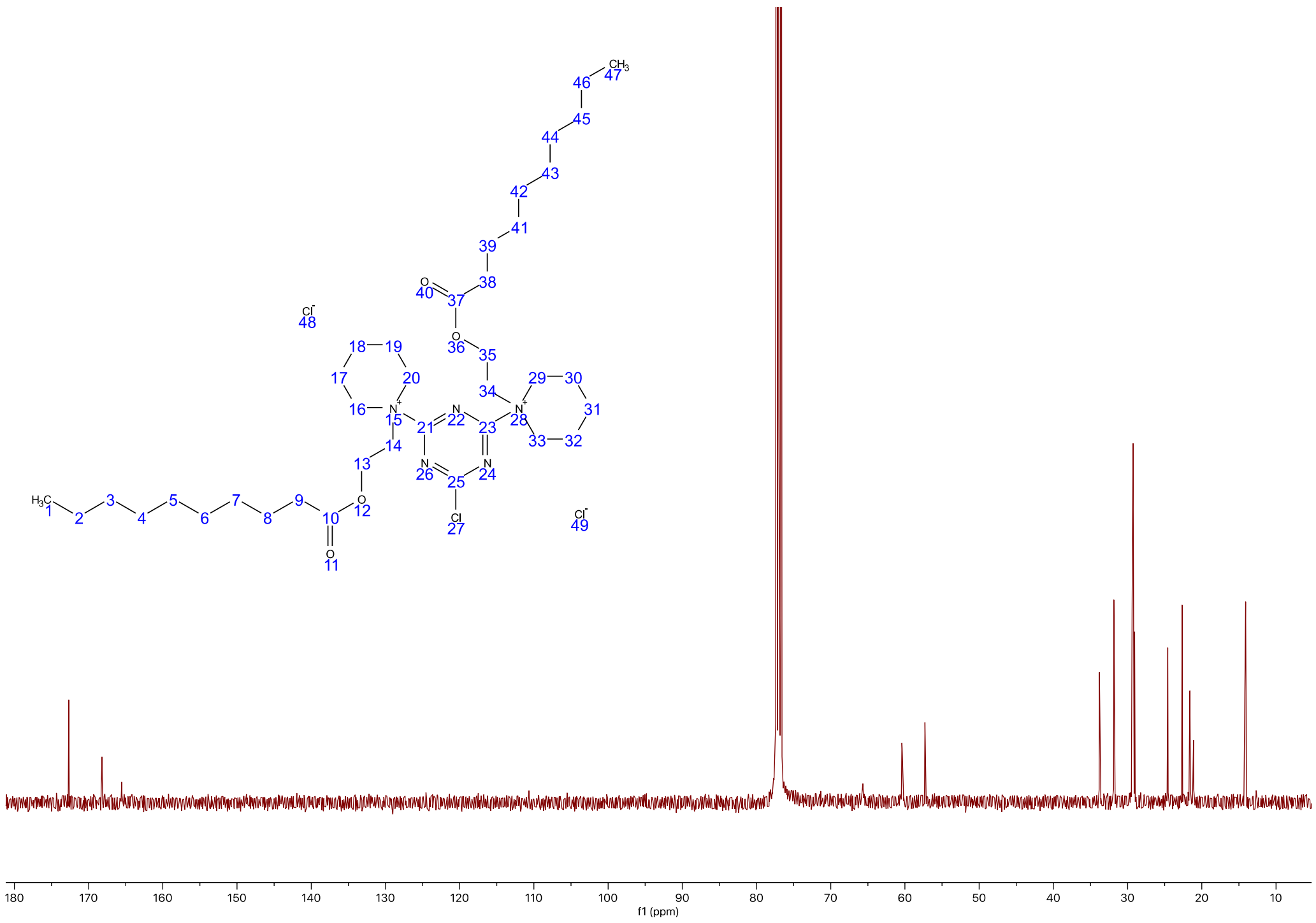

^{13}C NMR (400 MHz, $CDCl_3$) δ (ppm): 171.9 (C10 e C37), 168.15 (C23 e C21), 165.70 (C25), 65.83 (C13 e C35), 60.45 (C20-C16-C29-C33), 57.49 (C14 e C34), 33.95 (C9 e C38), 32.05 (C7 e

C41), 29.40 (C 3-7 e C 47-42), 24.74 (C8 e C39), 22.84 (C2- C46) 21.65 (C18 e C31), 21.30 (C19-C17, C32-C30), 14.17 (C1 e C47).

COSY

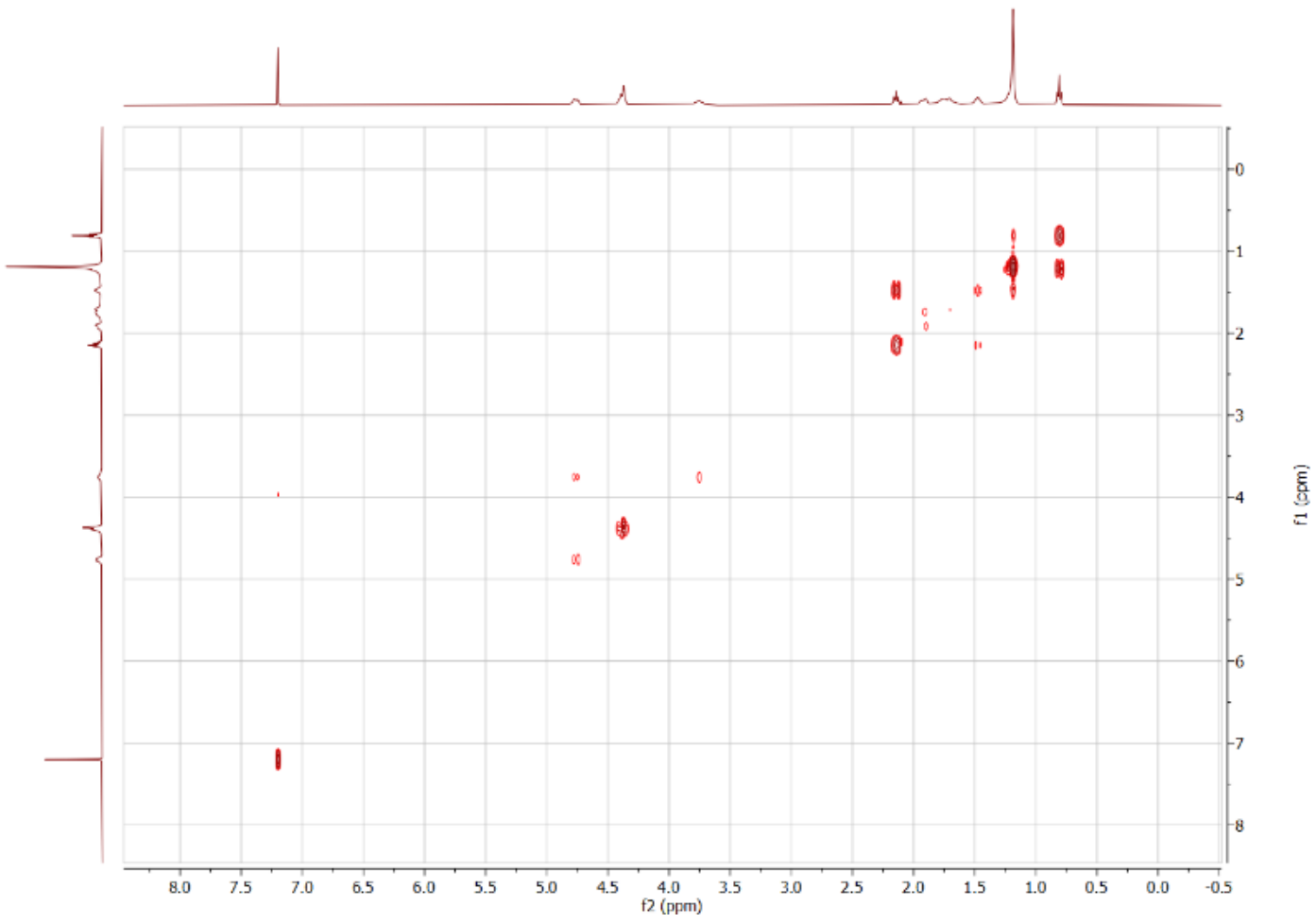

HSQC

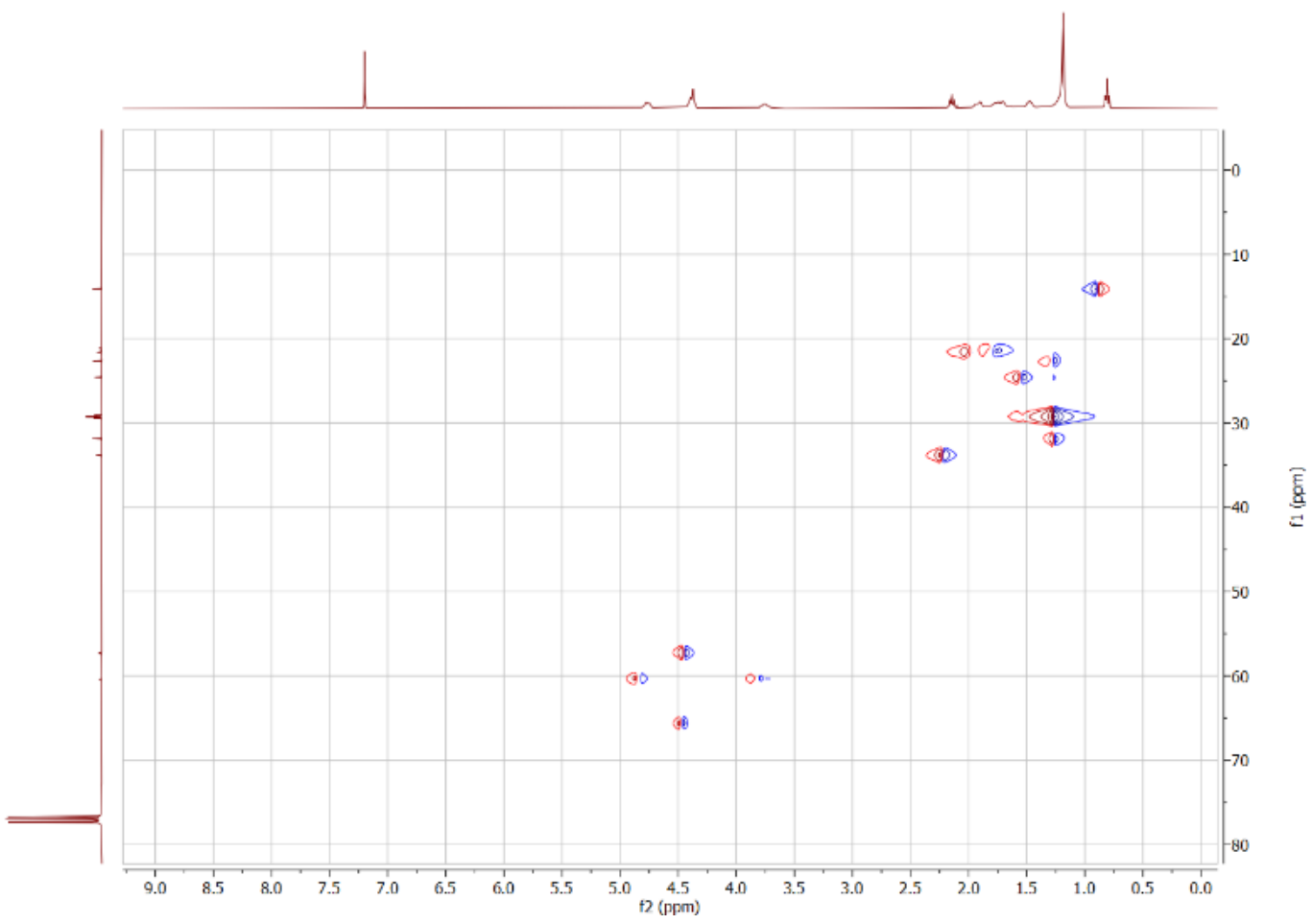

HMQC

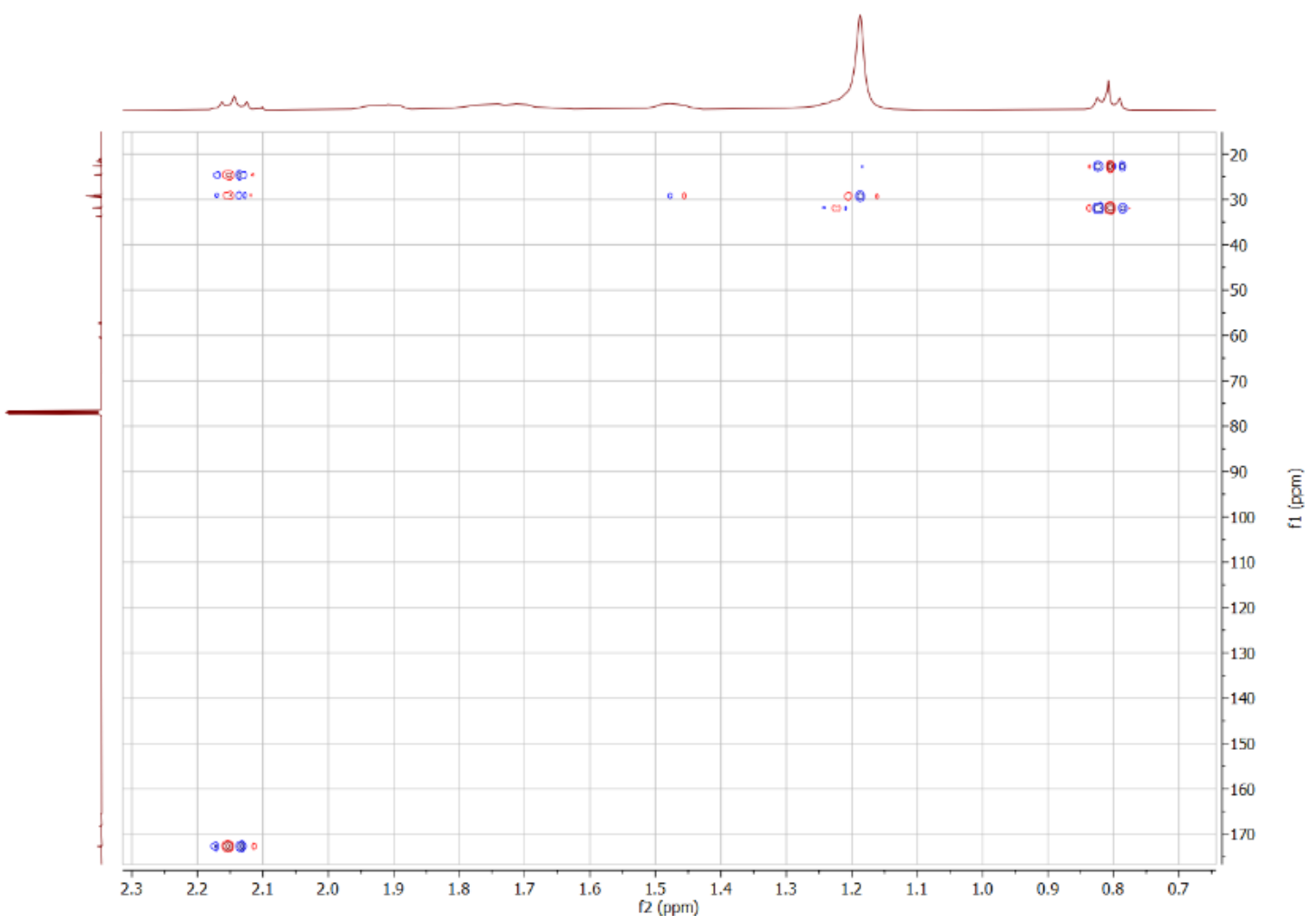

IV.14 2-chloro-4,6-(*N*-(ethylpiperidine)dodecanoate)-1,3,5-triazin chloride (12)

^{1}H NMR

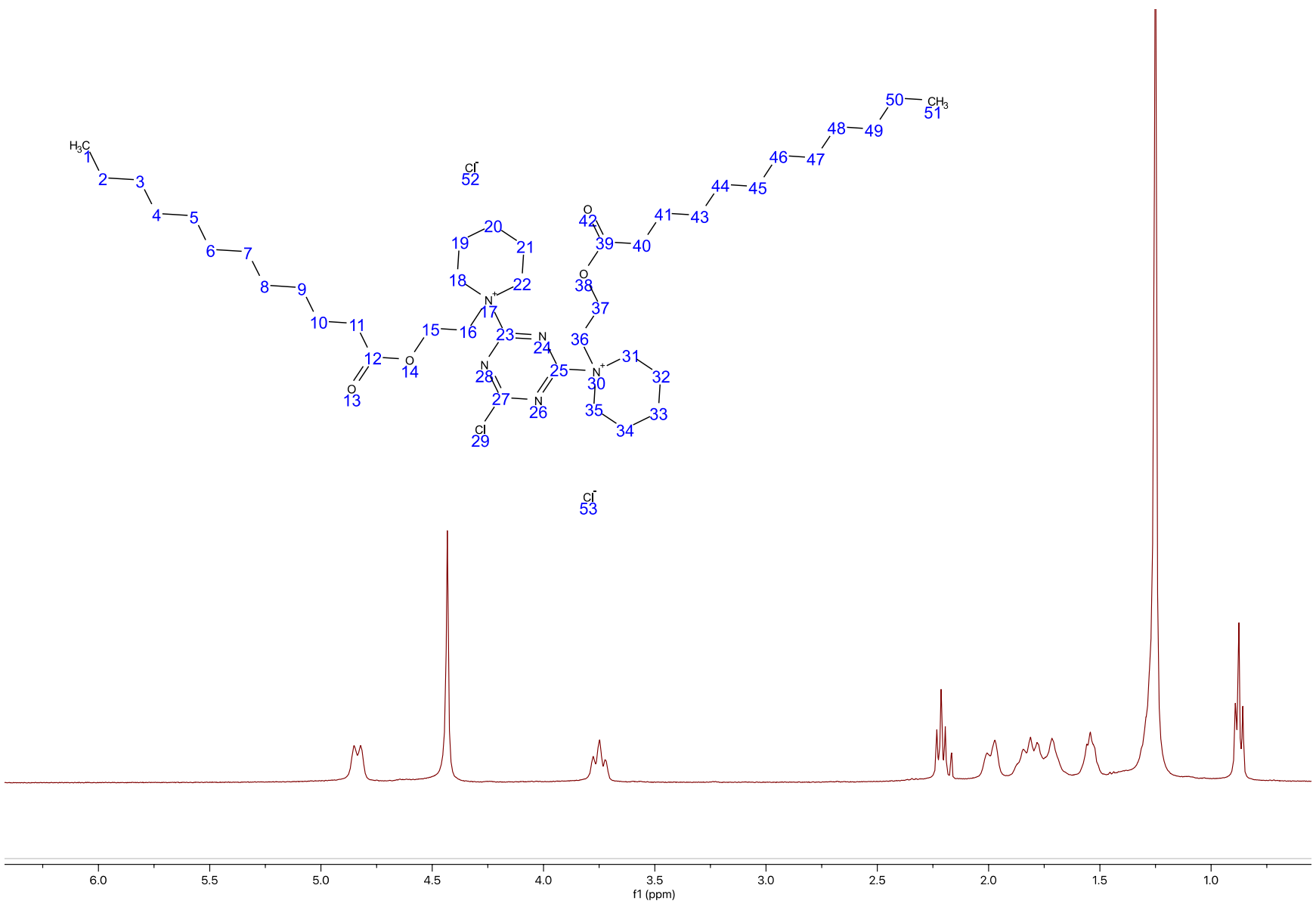

^{1}H NMR (400 MHz, $CDCl_3$) δ (ppm): 4.84 (4H, s, H 18-22 e H 31-35), 4.43 (8H, H 15-16 e H 36-37), 3.82 (4H, t, H 18-22 e H 31-35), 2.21 (4H, t, H11 e H40), 1.89 (4H, H20 e H33), 1.77 (8H, m, H 19-21 e H 34-32), 1.54 (4H, t, H10 e H41), 1.25 (C 2-9 e C 50-43), 0.88 (C1 e C51).

^{13}C NMR

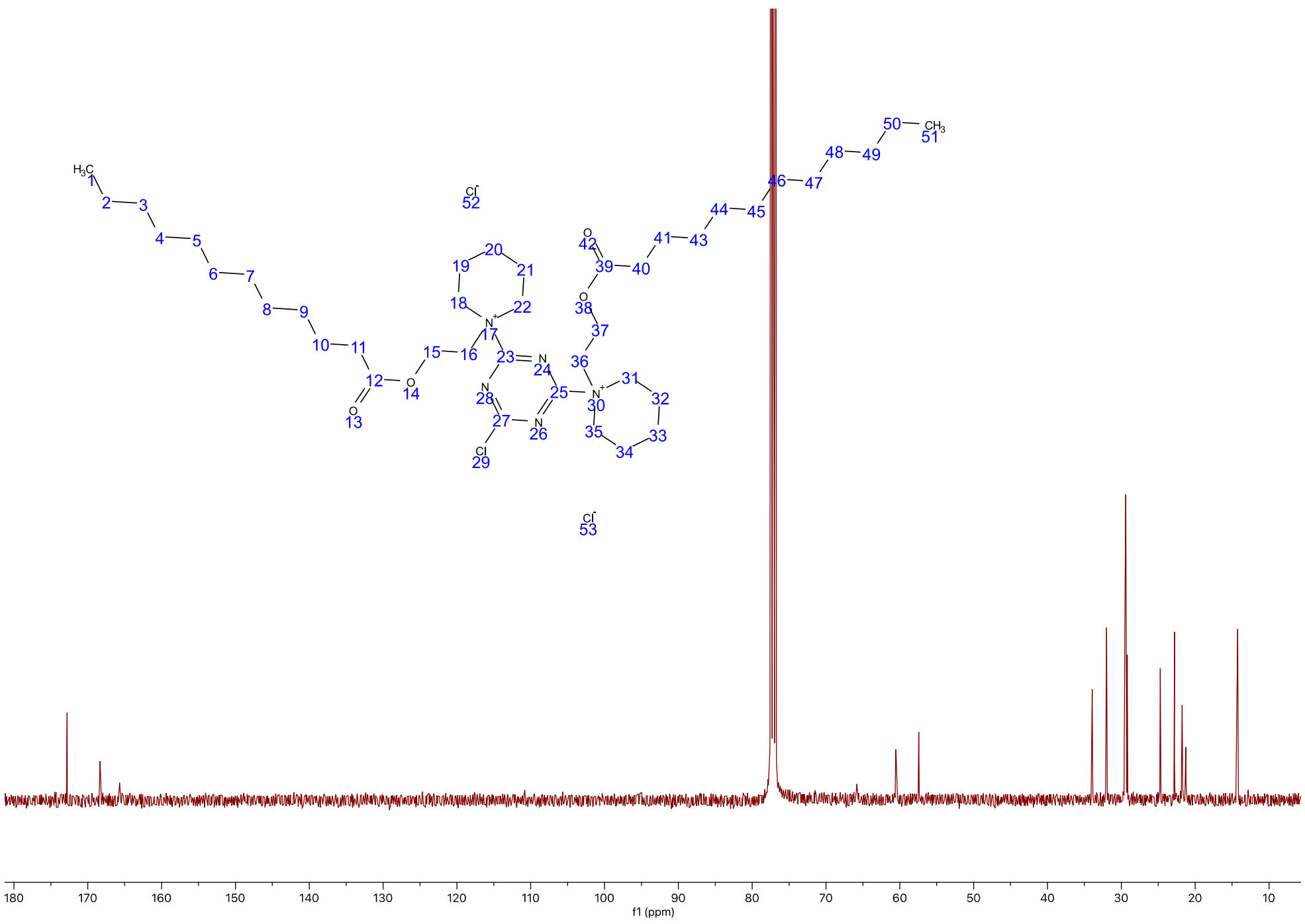

^{13}C NMR (400 MHz, $CDCl_3$) δ (ppm): 172.9 (C12 e C39), 168.36 (C25 e C23), 165.82 (C27), 65.83 (C15 e C37), 60.53 (C18-C22-C31-C35), 57.49 (C16 e C36), 33.95 (C11 e C40), 32.05 (C9 e C43), 29.38 (C 3-8 e C 49-44), 24.74 (C10 e C41), 22.84 (C2- C50) 21.74 (C20 e C33), 21.30 (C21-C19, C32-C34), 14.27 (C1 e C51).

COSY

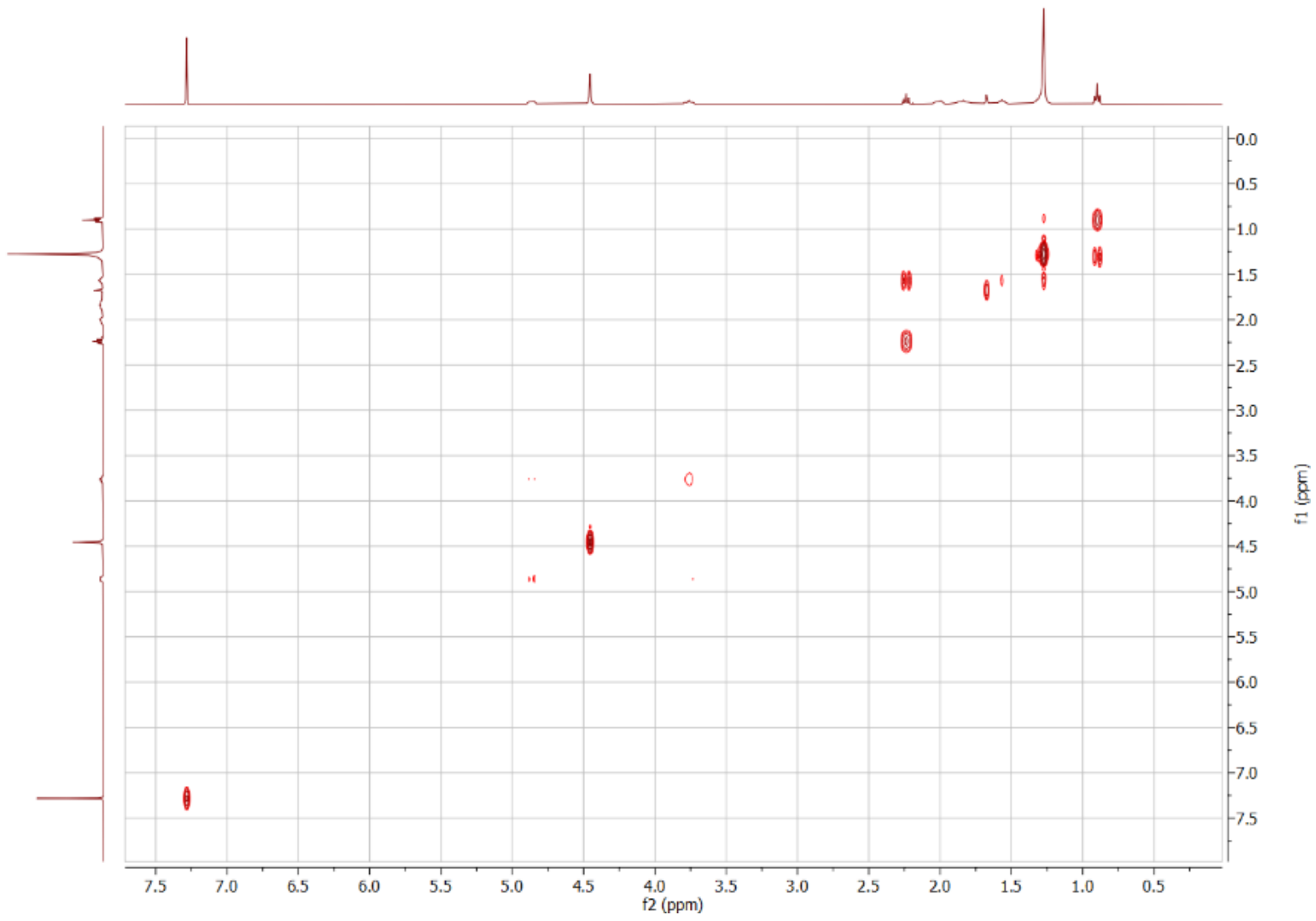

HSQC

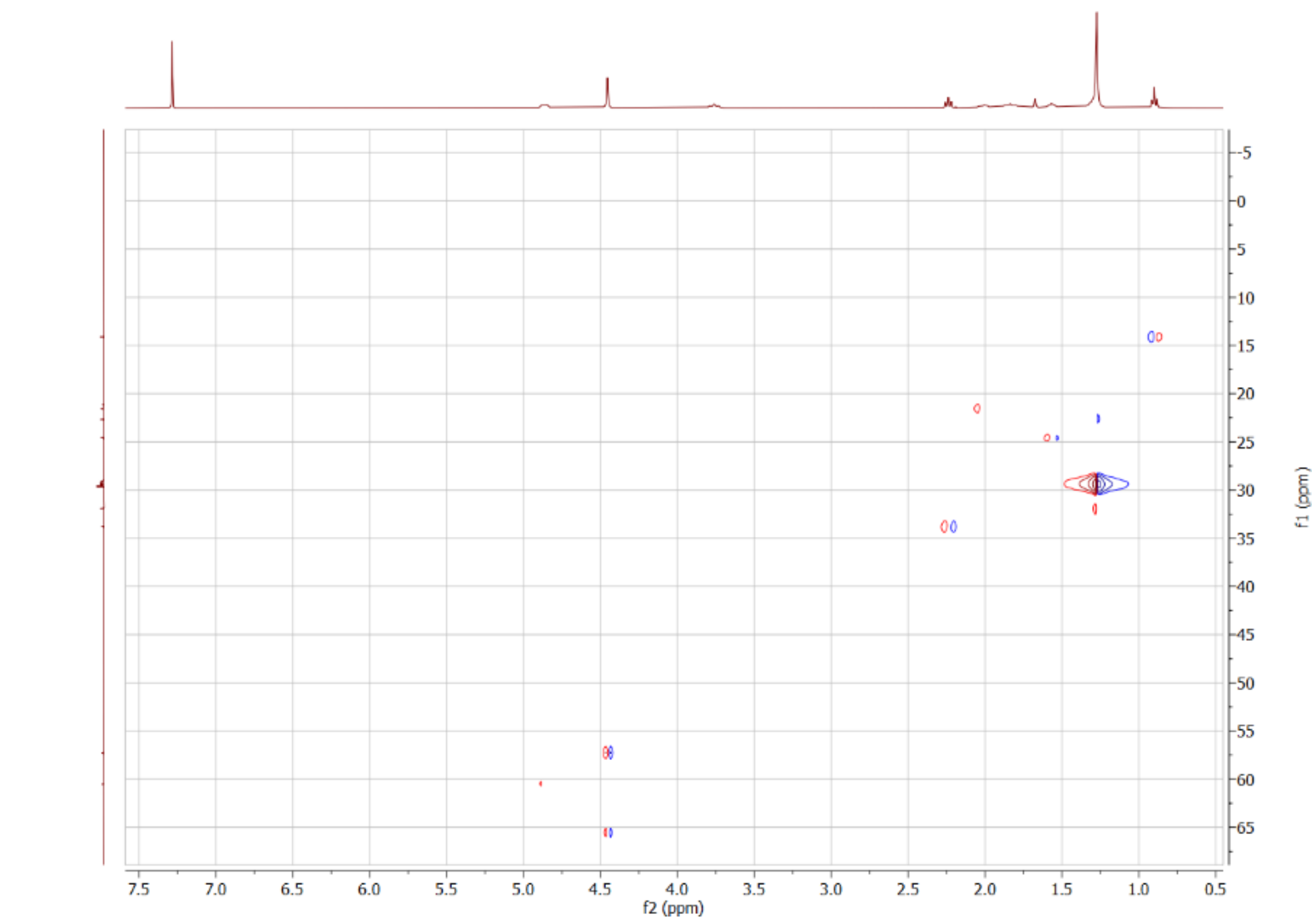

HMQC

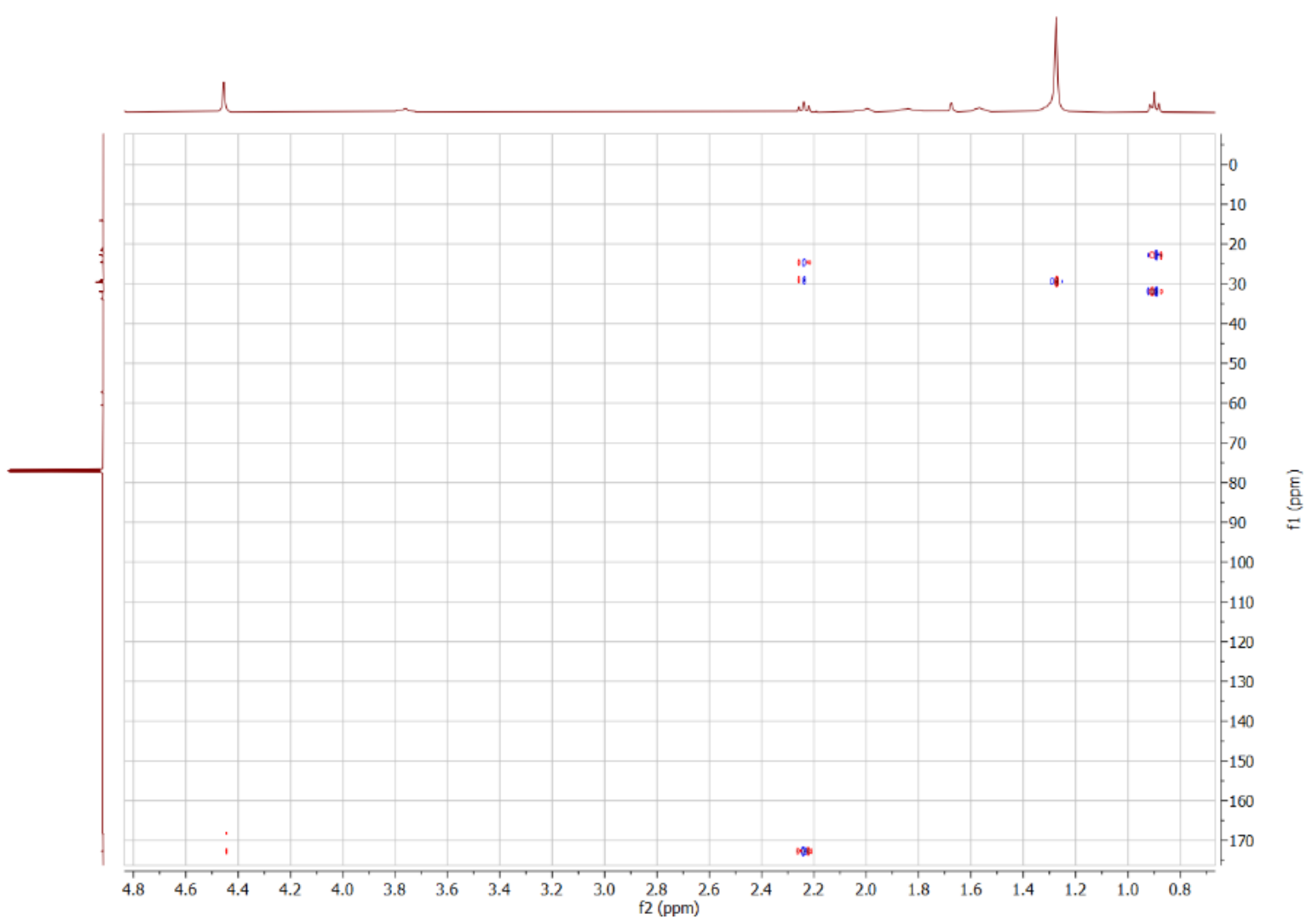

Supplement: Supplementary file 1 [file CMDC-21-e70458-s001.pdf]
